# Positive Feedback-Loop of Telomerase Reverse Transcriptase and 15-Lipoxygenase-2 Promotes Pulmonary Hypertension

**DOI:** 10.1371/journal.pone.0083132

**Published:** 2013-12-23

**Authors:** Tingting Shen, Jun Ma, Lei Zhang, Xiufeng Yu, Mengmeng Liu, Yunlong Hou, Yanyan Wang, Cui Ma, Shuzhen Li, Daling Zhu

**Affiliations:** 1 Department of Biopharmaceutical Sciences, College of Pharmacy, Harbin Medical University (Daqing), Daqing, Heilongjiang Province, China; 2 Biopharmaceutical Key Laboratory of Heilongjiang Province, Harbin Medical University, Harbin, Heilongjiang Province, China; 3 Department of Pharmacology (the State-Province Key Laboratories of Biomedicine-Pharmaceutics of China, Key Laboratory of Cardiovascular Research, Ministry of Education), Harbin Medical University, Harbin, Heilongjiang Province, China; University of Illinois College of Medicine, United States of America

## Abstract

**Objective:**

Pulmonary hypertension (PH) is characterized with pulmonary vasoconstriction and vascular remodeling mediated by 15-lipoxygenase (15-LO)/15-hydroxyeicosatetraenoic acid (15-HETE) according to our previous studies. Meanwhile, telomerase reverse transcriptase (TERT) activity is highly correlated with vascular injury and remodeling, suggesting that TERT may be an essential determinant in the development of PH. The aim of this study was to determine the contribution and molecular mechanisms of TERT in the pathogenesis of PH.

**Approach and Results:**

We measured the right ventricular systolic pressure (RVSP) and ventricular weight, analyzed morphometric change of the pulmonary vessels in the hypoxia or monocrotaline treated rats. Bromodeoxyuridine incorporation, transwell assay and flow cytometry in pulmonary smooth muscle cells were performed to investigate the roles and relationship of TERT and 15-LO/15-HETE in PH. We revealed that the expression of TERT was increased in pulmonary vasculature of patients with PH and in the monocrotaline or hypoxia rat model of PH. The up-regulation of TERT was associated with experimental elevated RVSP and pulmonary vascular remodeling. Coimmunoprecipitation experiments identified TERT as a novel interacting partner of 15-LO-2. TERT and 15-LO-2 augmented protein expression of each other. In addition, the proliferation, migration and cell-cycle transition from G_0_/G_1_ phase to S phase induced by hypoxia were inhibited by TERT knockdown, which were rescued by 15-HETE addition.

**Conclusions:**

These results demonstrate that TERT regulates pulmonary vascular remodeling. TERT and 15-LO-2 form a positive feedback loop and together promote proliferation and migration of pulmonary artery smooth muscle cells, creating a self-amplifying circuit which propels pulmonary hypertension.

## Introduction

Pulmonary hypertension (PH) is a progressive and refractory disease which often occurs in adult and pediatric patients with various pulmonary and cardiac diseases or as a vascular complication of HIV infection [1], [2]. Increased pulmonary vascular resistance leads to reinforced right ventricular systolic pressure (RVSP), right ventricular hypertrophy (RVH), right heart failure and ultimately death [3]. The modern treatment has improved the survival rate of PH from 68% (CI, 61–75%) to 83% (CI, 72–95%) and from 48% (CI, 31–55%) to 58% (CI, 49–69%) at 1- and 3- year, respectively [4], [5], however, there is still poor prognosis and no “cure” for this devastating disease. A pathological hallmark of many forms of PH is vascular remodeling, which results in narrowing and obstruction of pulmonary arterioles. These fundamental structural changes are caused by increased migration and proliferation of smooth muscle cells, adventitial fibroblast proliferation, extracellular matrix deposition, as well as abnormal endothelial cell proliferation [6], [7]. Although many central molecules and molecular pathways have been elucidated, the pathogenesis of PH involves a multi-factorial process and is not fully understood.

Telomerase reverse transcriptase (TERT), which confers the catalytic activity of telomerase [8], [9], is the restricting factor for telomerase activity [10]. TERT and its programmed phosphorylation are highly correlated with the proliferation of vascular smooth muscle cells [11], [12], [13]. Moreover, TERT activity has been detected in vascular injury and remodeling [14], inflamed lungs [15], injured livers [16], and hypertensive blood vessels [17], indicating a potential role of TERT in the pulmonary vascular disease, including PH. Consequently, the role of TERT in the proliferation and migration of pulmonary artery smooth muscle cells (PASMCs) which is associated with hypoxia-induced PH needs to be determined.

A heterogeneous family of lipidperoxidizing enzymes composes lipoxygenases (LOs) which are divided into 5-, 8-, 12-, and 15-LOs [18]. Both 15-LO-1 and 15-LO-2 are expressed in humans in a tissue-specific manner and convert arachidonic acid to 15-hydroxyeicosatetraenoic acid (15-HETE) [19]. Previous studies from our laboratory have shown that chronic hypoxia augments the content of endogenous 15-HETE through increased 15-LO activation [20]. 15-HETE inhibits apoptosis and propels proliferation of pulmonary smooth muscle cells, driving pulmonary vascular remodeling associated with hypoxia-induced PH [21], [22], [23]. This paper further characterized the mechanism how 15-LO/15-HETE regulated pulmonary vascular remodeling induced by hypoxia.

In this study, we suppressed TERT activity with the reverse transcriptase inhibitor azidothymidine (3′-Azido-3′-deoxythymidine; AZT) in rats [24], [25] and silencing the gene of TERT in PASMCs. We found that TERT affected the expression of 15-LO and the production of 15-HETE. Furthermore, we demonstrated a mutual positive regulation between TERT-15-LO/15-HETE pathway *in vivo* and *in vitro* and showed the interaction between TERT and 15-LO. Ultimately, we elucidated that 15-HETE/TERT positive feedback loop mediated the migration, proliferation and cell cycle distribution of PASMCs in hypoxia-induced pulmonary vascular remodeling.

## Materials and Methods

### Ethics Statement

Written informed consent was obtained from all subjects. The work was approved by the Harbin Medical University Ethical Committee for Use of Human Samples.

All experimental procedures in animals followed the guidelines of, and were approved by the Institutional Animal Care and Use Committee, and were conducted in compliance with the NIH guide for the Care and Use of Laboratory Animals. The protocol was approved by the Committee on the Ethics of Animal Experiments of the Harbin Medical University (Permit Number: 2010-0006). All surgery was performed under sodium pentobarbital anesthesia, and all efforts were made to minimize suffering.

### Human Lung Samples

Control lung tissues were from lobectomy for right lower lung lobe spherical lesions. HPH lung specimens were obtained from PH patients, two human lung specimens of them were obtained from lung transplantation (PH) patients with idiopathic pulmonary artery hypertension (IPAH), one was obtained from autopsy of patients with IPAH, two were obtained from autopsy of patients with pulmonary artery hypertension secondary to chronic obstructive pulmonary disease (COPD). The human lung specimens were separately collected within two years in the Harbin Medical University Fourth Affiliated Hospital.

### Animals and Treatments

Adult male Wistar rats (average weight of 200 g) from the Harbin Medical University Experimental Animal Center were randomly assigned to groups of control, hypoxia, monocrotaline (MCT, Sigma, 60 mg/kg, single subcutaneously) injection as described previously [20]. Rats upon hypoxia were treated with nordihydroguaiaretic acid (NDGA, Cayman, CAS 500-38-9, 650 mg/kg body weight, orally once daily, for 11 days) [26], [27] or azidothymidine (AZT, Sigma, A2169, 20 mg/kg body weight, intraperitoneally once daily, for 14 days) [25] from 2 days before hypoxia till they were anesthetized. Rats injected with MCT were treated with AZT 7 days after MCT administration (the 8th day).

### Hemodynamic Analysis and Ventricular Weight Measurement

At the end of the treatment protocol, the animals were anesthetized with pentobarbital intraperitoneally. They were artificially ventilated with 10 ml per kg body weight and a frequency of 60 s^−1^ (SAR830A/P, IITC) after tracheostomy. Inspiratory oxygen (FIO_2_) was set at 0.5, and a positive end-expiratory pressure of 1.5 cm H_2_O was used throughout. Anesthesia was maintained by inhalation of isoflurane. The left carotid artery was cannulated for systemic arterial pressure (SAP) monitoring, and a Millar 847 (Millar Instruments Inc, Houston, Tex) catheter was inserted through the right jugular vein for measurement of right ventricular systolic pressure (RVSP) with PowerLab (ADI Instruments, Colorado Springs, Colo) monitoring equipment. Cardiac output (CO) was measured by thermodilution technique (Cardiotherm 500-X, Hugo-Sachs Electronic, Harvard Apparatus GmbH). CO was averaged from three consecutive determinations by the software of LabChart 7.0 and indexed to the weight of the animal to obtain cardiac index. For ventricular weight measurement, hearts were excised and atria were removed. The right ventricle (RV) free wall was dissected, and each chamber was weighed. The ratio of RV weight to left ventricular (LV) weight plus septum (RV/LV+S) was used as an index of RV hypertrophy. After exsanguination, the lung was fixed for histology in 4% paraformaldehyde.

### Morphometric Analysis of the PA

The left lung tissues were sliced into tissue blocks, and immersed in 4% paraformaldehyde for overnight fixation [21]. The fixed tissues were dehydrated, cleared, and embedded in paraffin waxes. The paraffin blocks were cut into 5 µm thick sections. Some sections were stained with hematoxylin and eosin (H&E) and the others were stained with Masson trichrome. For immunohistochemistry, 5-µm paraffin-embedded tissue sections were deparaffinized and rehydrated in graduated alcohol. Then the tissue sections were treated in a 0.1 mol/L of sodium citrate buffer and heated for 20 min for antigen retrieval. After they were cooled down, the endogenous peroxidase activity was blocked, and then the sections were incubated with TERT (Abcam, ab32020, 1∶50), 15-LO-2 (Cayman, Catalog No. 10004454, 1∶200), and OPN (Santa Cruz, sc-73631, 1∶5) antibodies. Parallel controls were run with PBS. After an overnight incubation, the sections were washed three times with PBS and then subjected to the secondary antibodies (1∶200) for the IgGs of the appropriate species. The sections were visualized with 3, 3-diaminobenzidine (DAB) and counterstained using hematoxylin. Brown and yellow colors indicated positive stains. The total area of collagen and the positive staining area of TERT, 15-LO-2 and OPN in the vascular walls were quantified on high-resolution images of individual vessel using a color-recognition algorithm in Image Pro Plus 6.0.

Meanwhile, the right lung tissues were fixed in 4% paraformaldehyde for 6 hours, transferred to 10%, 20%, 30% sucrose in 0.1 mol/L phosphate buffer (pH 7.4) for 12 h each for cryoprotection, and stored at 4°C. Lung tissue was frozen in Tissue-Tek OCT compound (Sakura Finetechnical Co) at −20°C. Then, 7-µm sections were cut using a cryostat. The cryosections were blocked with 10% normal goat serum/PBS for 30 min. TERT antibody (Abcam, ab32020, 1∶50) or α-SMA (Santa Cruz, sc-130617, 1∶200) were incubated at 4°C overnight. After washing three times with PBS, sections were incubated with Alexa Fluor 488 conjugated goat anti-rabbit antibody (Invitrogen, A-11070, 1∶2000) or Alexa Fluor 546 conjugated goat anti-mouse antibody (Invitrogen, A-11018, 1∶2000) for 2 h at room temperature and incubated with DAPI (Boster, AR1176) for 15 minutes in dark. Sections were washed three times with PBS and then examined with a microscope (Olympus, Japan), and images were recorded by digital photomicrography (Olympus, Japan).

### Cell Culture

PASMCs were dispersed from adult male Wistar rats (average weight of 200 g) from the Harbin Medical University Experimental Animal Center according to our previously published protocol [27]. Cells were cultured in 20% fetal bovine serum (FBS)-DMEM (Gibco) in a 37°C, 5% CO_2_ humidified incubator. Cell viability determined by Trypan Blue exclusion was consistently greater than 98%. The purity of PASMCs in the primary cultures was determined by specific monoclonal antibodies raised against α-smooth muscle actin. Before each experiment, the proliferation in PASMC was induced by serum deprivation incubated in DMEM without serum for 24 h. Then some cells were treated with siRNA plus 15-HETE (1 mM) in 5% FBS-DMEM, the others were exposed to hypoxia in the absence or presence of CDC (5 µM), NDGA (30 µM). The cells cultured in complete medium were used as control. CDC, NDGA and 15-HETE at the indicated concentrations were replaced every 24 h with new medium.

### Cell Cycle and DNA Analysis

The CycleTEST™ PLUS DNA Reagent Kit obtained from BD Biosciences (Bedford, MA, USA) was used for examining whether the cell cycle was regulated by TERT. PASMCs were treated in groups as indicated and then harvested with trypsin and fixed using 70% ethanol. The ethanol was removed, and the cells were incubated in 200 µl PBS. The cells were stained according to manufacturer’s protocol. DNA fluorescence was measured and flow cytometry was performed using BD FACS Calibur Flow Cytometer (Bedford, MA). For each sample, 2×10^4^ events were accumulated in a histogram. The proportions of cells in the different phases of the cell cycle were calculated from each histogram.

### Immunofluorescence Study and Microscopic Observation

PASMCs were cultured on a cover glass (15 mm diameter). After treatment, cells were fixed with 4% paraformaldehyde in PBS at room temperature for 15 min, permeabilized with 0.5% Triton X-100 for 10 min, blocked with 3% normal bovine serum in PBS at 37°C for 30 min, followed by incubating with anti-TERT primary antibody (Abcam, ab32020, 1∶50) in PBS at 4°C overnight. After washing three times with PBS, the cells were incubated with Alexa Fluor 488 conjugated goat anti-rabbit antibody (Invitrogen, A-11070, 1∶2000) diluted with PBS at 37°C for 2 h and DAPI protected from light. Slides were then examined with a microscope (Olympus, Japan), and images were recorded by digital photomicrography (Olympus, Japan), pulmonary sections were evaluated under a Zeiss Confocal Microscope (model LSM-510; Carl Zeiss Microimaging, Inc., Thornwood, NY).

### siRNA Design and Transfections

To silence the expression of TERT, and 15-LO-2 protein, PASMCs were transfected with small interfering RNA, which was designed and synthesized by GenePharma. Non-targeted control siRNA (siNC) was used as negative control. The sense sequence of siRNA against TERT, 15-LO-2 and non-targeted control sequence were listed below with accession numbers: TERT (NM_053423.1): 5′-CGAGUGACAGCUACAGGAU-3′; 15-LO-2 (NM_153301.2): 5′-GCAAUGAAGAACGCCAAAUTT-3′; NC control: 5′-CCUACGCCACCAAUUUCGU-3′. Briefly, the PASMCs were cultured till 50%–70% confluence and then 1.5 µg siRNA and 7.5 µl X-tremeGene siRNA Transfection Reagent were separately diluted in 100 µl serum-free Opti-MEM-1 medium and were mixed together 5 min later. The mixture (siRNA/Transfection Reagent) was incubated at room temperature for 20 min and added directly onto the cells. Cells were quiescenced for 24 h and used as required.

### MTT Assay

PASMCs were cultured at a density of 5,000 cells/well in a 96-well culture plate and then treated with siRNA, 15-HETE in DMEM with 5% FBS. At the end of incubation at 37°C, the cells were incubated for 4 h in a medium containing 0.5% 3-[4, 5-dimethylthiazol-2-yl]2, 5-diphenyl-tetrazolium bromide (MTT, Invitrogen, M-6494), the yellow mitochondrial dye. The amount of blue formazan dye formed from MTT is proportional to the number of survival cells. The reaction was terminated by adding 150 µl DMSO and incubating for 10 min. Absorbance at 540 nm was recorded by an ELISA plate reader.

### Bromodeoxyuridine Incorporation

PASMCs were plated in 96-well plates at the density of 5,000 cells/well, and then subjected to growth arrest for 24 h before treatments with different agents in 5% FBS DMEM. We measured BrdU incorporation according to the Millipore BrdU proliferation assay kit (Catalog *No*. 2750, Billerica, MA, USA) instructions. Briefly, after treatment, the cells were incubated with 15 µM BrdU labeling solution per well for 24 h at 37°C, and then incubated for 30 min in FixDenat solution at room temperature. Flicking off the FixDenat solution thoroughly, we added anti-BrdU-monoclonal solution 200 µl/well for 1 h at room temperature. Each well was rinsed three times with 200 µl washing solution and incubated for 30 min in 100 µl substrate solution. After adding 100 µl stop solution, the absorbance at 450 nm of the samples was recorded in an ELISA reader.

### Western Blot Analysis and Immuprecipitation

Pulmonary arteries from rats (normoxia, hypoxia and hypoxia with NDGA) were homogenized in a hand-held micro-tissue grinder in ice-cold storage buffer (Tris 50 mM, pH 7.4, NaCl 150 mM, Triton X-100 1%, EDTA 1 mM, and PMSF 2 mM). The homogenates were sonicated on ice and then centrifuged at 16,099 g for 10 min at 4°C. The supernatants were collected and stored at −80°C until used in Western blot analysis.

After treatments in 6-well culture clusters for 24 h, the cells were lysed in a lysis buffer (Tris 50 mM, pH 7.4, NaCl 150 mM, Triton X-100 1%, EDTA 1 mM, and PMSF 2 mM) containing phosphatase inhibitor and incubated for 30 min on ice. The lysates were then sonicated and centrifuged at 16,099 g for 10 min, and the insoluble fraction was discarded. The supernatants were collected and stored at −80°C until used in Western blot analysis.

Protein concentrations were determined by the Bradford assay using bovine serum albumin (BSA) as standard. Pulmonary artery homogenates containing 50 µg of protein and cells protein samples containing 20 µg of protein were separated by SDS–PAGE as previously described. After electrophoresis, proteins were transferred to nitrocellulose sheets. These membranes were blocked in 5% milk and incubated with TERT (Santa Cruz,sc-7212, 1∶400), pCNA (Santa Cruz, sc-25280, 1∶1000), cyclin A (Santa Cruz, sc-596, 1∶500), cylin D_1_ (Cell Signal, Catalog No. 2978S, 1∶400), OPN (Santa Cruz, sc-20788, 1∶400), 15-LO-2 (Cayman, Catalog No. 10004454, 1∶400), β-actin (Santa Cruz, sc-47778, 1∶2000) and secondary antibodies as described. These proteins were visualized with enhanced chemiluminescence reagents (SuperSignal, Pierce).

For immunoprecipitation of 15-LO-2, 100 µg cell lysate were incubated with 2 µg mouse monoclonal 15-LO-2 antibody (Santa Cruz Biotechnology, sc-376871) and thereafter, were incubated with Protein A/G-Sepharose beads (Santa Cruz Biotechnology). Proteins were separated by 10% SDS/PAGE and electrotransferred into nitrocellulose filters. Membranes were incubated with rabbit-anti TERT antibodies overnight at 4°C. Membranes were incubated with HRP conjugated anti-rabbit secondary antibody for 1 h at room temperature, and protein bands were visualized with enhanced chemiluminescence reagents (SuperSignal, Pierce).

### Measurement of 15-HETE Level

To examine whether silencing the gene of TERT can inhibit the generation of endogenous 15-HETE in PASMCs, the 15(S)-HETE EIA Kit (Catalog No. 534721, Cayman) was utilized for the detection of the amount of 15(S)-HETE. Pulmonary artery smooth muscle cells were lysed in a lysis buffer (Tris 50 mM, pH 7.4, NaCl 150 mM, Triton X-100 1%, EDTA 1 mM, and PMSF 2 mM) and incubated for 30 min on ice. Then the amount of endogenous 15-HETE was measured by 15(S)-HETE EIA Kit. The protein concentrations were determined by Bradford protein assay. The results were analyzed with Cayman Chemical Company Enzyme Immunoassay (EIA) Tools.

### RT-PCR

PASMC RNA was extracted using Trizol reagent and then reverse-transcribed using SuperScript TM First-Strand Synthesis System followed by amplification in a DNA thermal cycler (PxG20809, Thermo Electron Co.), using Taq polymerase. The gene-specific primers were designed from coding regions and the nucleotide sequences of primers were as follows: TERT (GenBank accession no NM_001192974.1), sense: 5′-TGTTCCTGTTCTGGCTAATGG-3′, anti-sense: 5′-GATGTTTGGTCCGCTCGTAG-3′, 390 bp); β-actin (GenBank accession no. NM_031144.2): sense: 5′-TTGTAACCAACTGGGACGATATGG-3′, anti-sense: 5′- GATCTTGATCTTCATGGTGCTAGG-3′. The thermal cycles were 40 cycles of 94°C for 30 s, 51°C for 30 s, and 72°C for 30 s (for TERT) and 35 cycles of 94°C for 30 s, 51°C for 30 s, and 72°C for 30 s (for β-actin). The amplified products were visualized on a 1.2% agarose gel stained by ethidium bromide by UV trans-illumination. Quantitative analysis was carried out using Quantity One 1-D software (Bio-rad).

### Migration Assays

For scratch-wounding cell migration assay, the confluent PASMCs cultured in 6-well plates were wounded by pipette tips, given rise to one acellular 1-mm-wide lane per well, and the ablated cells were washed out with PBS. After that, cells were treated with vehicle or chemicals of interest in 5% FBS DMEM. Wounded areas were photographed at time zero. After 6 h, 24 h of incubation, photos were taken from the same areas as those recorded at time zero.

For modified Boyden chamber migration assays (Corning, Catalog No. 3422, USA), cells were subcultured once, before seeding into the apical (upper) chamber of the transwells. The lower chamber contained the experimental reagents in 10% FBS-DMEM, migration was measured using a modified Boyden chamber with 8-µm-pore polycarbonate filter. Briefly, 8×10^4^ PASMCs were suspended in DMEM without FBS in the upper chamber of a 24-well Boyden chamber apparatus with serum stimuli in the lower chamber for 24 hours, and then the inserts were removed. The non-migrating cells in the upper chamber were removed with a cotton swab. To stain the cells embedded in the bottom membrane, the inserts were submerged in 4% formaldehyde solution for 10 minutes followed by incubation in 0.4% crystal violet in 10% ethanol for 5 minutes. The number of migrated cells was measured by counting the number of stained nuclei per high-power field under a microscope (Olympus, Japan). Each sample was counted randomly in 9 separate locations in the center of the membrane and the smooth muscle cell migration activity was reported as number of cells migrated per field of view. Experiments were performed at least three times in quadruplicate.

### Ultra Performance Liquid Chromatography (UPLC)

The contents of 15-HETE in rat lung tissues which contain pulmonary arteries were analyzed by UPLC according to the published method [20], [28]. Briefly, the tissues were homogenized within ethyl acetate which was acidified to pH 3.0 with formic acid and centrifuged at 15,000 rpm for 20 min at 4°C. The supernatants were collected, dried down under nitrogen, reconstituted in 0.8 ml of 20% acetonitrile: distilled water (pH 3.0), and applied to a Strata-X polymeric SPE column that had been preconditioned with 5 ml of 100% ethyl alcohol and 5 ml of 25% ethyl alcohol followed by distilled water and 25% ethyl alcohol. Thereafter, the eicosanoids were eluted from the column with ethyl acetate containing 5 ml of 0.0002% butylated hydroxytoluene. Endogenous 15-HETE was separated on a Waters Acquity™ UPLC BEH C18 column (2.1 mm×50 mm, 1.7 µm) which was maintained at 30°C. The mobile phase consisted of 0.05% aqueous formic acid (B) and methanol/acetonitrile(1∶4, v/v)(A) at a flow rate of 0.25 ml/min. The mobile phase gradient was ran from 30% A to 100% A for 7 minutes, returned to 30% A for 1.5 minutes, and was held at 30% A for 1.5 minutes for reequilibration. A TOF mass spectrometer equipped with an electrospray ionization (ESI) interface (Waters, LCT premier XE) was used in detecting the negative ionization of 15-HETE.

### Statistical Analysis

The data are presented as mean±SEM. Statistical analysis was performed with Student t test or one-way ANOVA followed by a Dunnett test where appropriate. P<0.05 was considered statistically significant.

## Results

### TERT Expression was Increased in PH Patients and TERT Inhibition Alleviated Pulmonary Vascular Remodeling (PVR) in Rats

**Figure 1:**
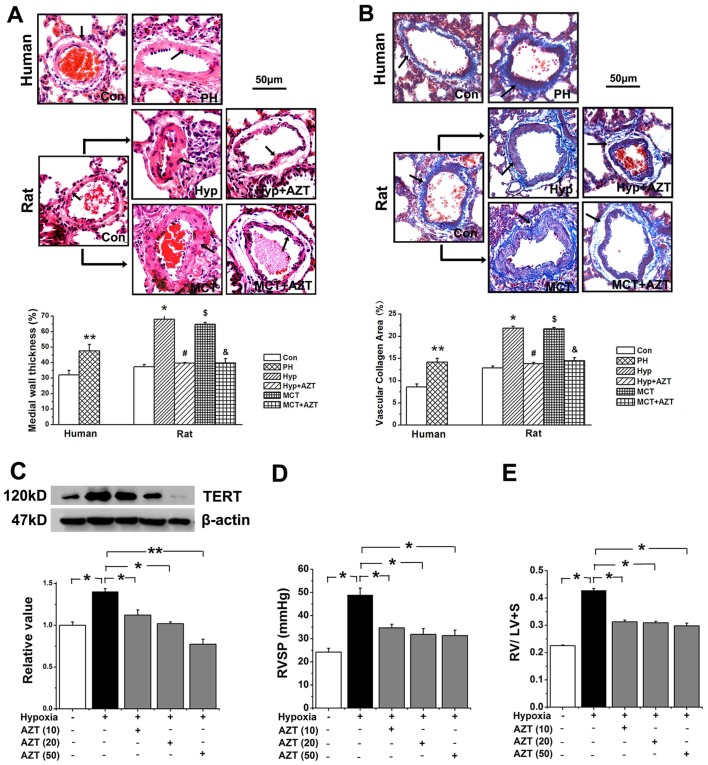
TERT inhibition by AZT alleviated PVR in rats. **A**, Hematoxylin-eosin (H&E) staining. **B**, Masson staining. The pulmonary vascular thickness (**A**), the deposition of collagen (**B**) were increased in the pulmonary vascular wall in PH patients (n = 5). The pulmonary vascular remodeling induced by hypoxia or MCT injection were inhibited by AZT administration (n = 6). Data show quantitative analyses of positive staining per vascular area (adventitia+media+intima+lumen). Con, control; Hyp, hypoxia; MCT, monocrotaline, A, azidothymidine. (**P<0.05 vs. control patients, *P<0.05 vs. normoxia, #P<0.05 vs. hypoxia, $P<0.05 vs. normoxia, &P<0.05 vs. MCT). The PAs in each group were obtained from three independent rats. **C**, The expression of TERT in pulmonary vessels of rats was inhibited in the dose-dependent effect. AZT (10) indicates 10 mg azidothymidine per kilogram body weight of rat; AZT (20), 20 mg/kg; AZT (50), 50 mg/kg. **D**, Right ventricular systemic pressure (RVSP) was inhibited by AZT in all three doses. **E**, Ratio of right ventricle to left ventricle plus septum was suppressed by AZT in all three upper doses. *P<0.05, **P<0.01; n = 6. All values are denoted as mean ± SEM.

In PH patients, vascular walls of the pulmonary arterioles were markedly thickened as observed with H&E staining (Figure 1A). Additionally, Masson staining showed that the area of collagen deposition in the adventitia was significantly increased in human PH patients than that of normal donors (Figure 1B). These phenotypes reproduced the pathological changes observed in hypoxia or MCT rats (Figure 1A, B). Nevertheless, AZT protected rats from upper mentioned morphometric changes induced by hypoxia or MCT.

**Figure 2:**
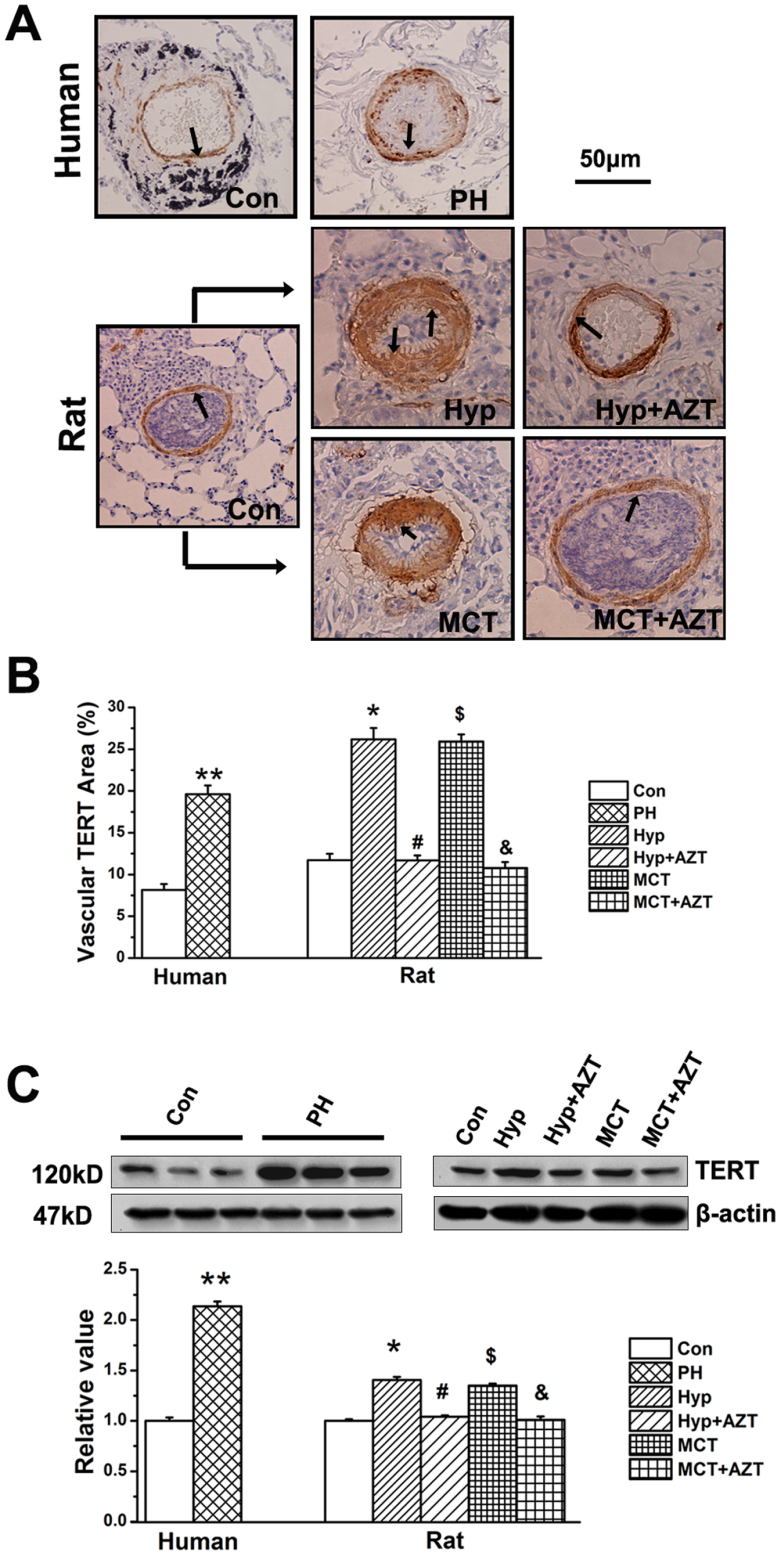
TERT expression increased in PH patients. **A**, TERT protein staining. TERT protein level was increased in the pulmonary vascular wall in PH patients (n = 5). In the animal experiments (n = 6), hypoxia or MCT administration significantly increased the expression of TERT protein compared with control rats, which were reversible by administration of azidothymidine (AZT, 20 mg/kg body weight). **B**, Quantitative analyses of positive staining per vascular area (adventitia+media+intima+lumen). **C**, TERT protein in lung tissues. Con, control; Hyp, hypoxia; MCT, monocrotaline, AZT, azidothymidine. (**P<0.05 vs. control patients, *P<0.05 vs. normoxia, #P<0.05 vs. hypoxia, $P<0.05 vs. normoxia, &P<0.05 vs. MCT). The PAs in each group were obtained from three independent rats and all values are denoted as mean ± SEM.

Moreover, the pulmonary vascular TERT protein was significantly increased in human PH patients, as well as in hypoxia or MCT treated rats (Figure 2A, 2B, 2C). Consistent with the previous results in the hippocamp [24] or liver [25], we proved that azidothymidine (AZT) could reduce the protein expression of TERT in pulmonary vessels (Figure 1C, 2A, 2B, 2C).

### TERT Inhibition Prevented Hypoxia-PH and MCT-PH

**Figure 3:**
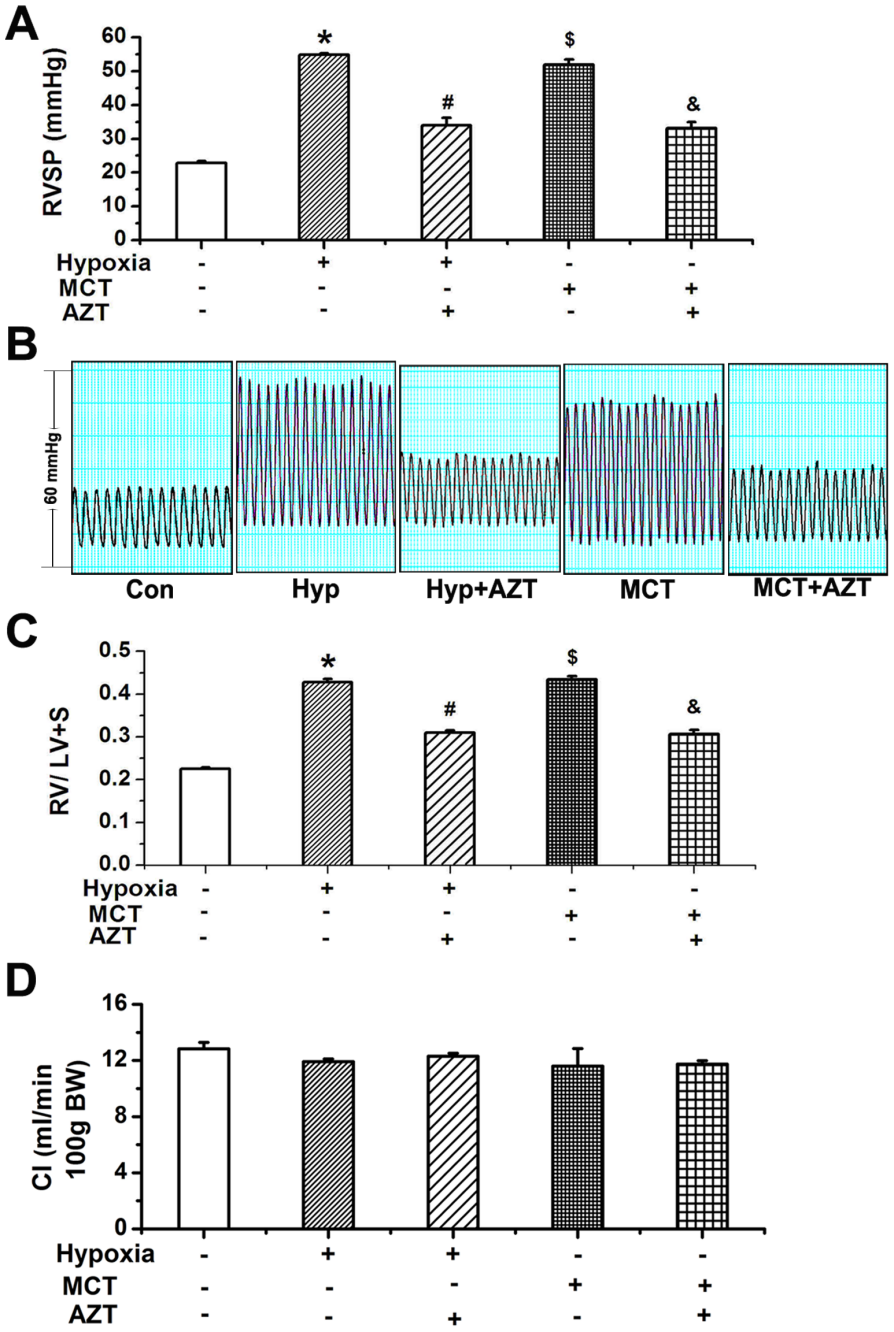
TERT inhibition reversed PH induced by hypoxia or MCT in rats. **A**, Right ventricular systemic pressures (RVSP). **B**, Representative tracing of RVSP. **C**, Ratio of right ventricle to left ventricle plus septum. **D**, Cardiac index (cardiac output per 100 g body weight). Hypoxia or MCT significantly increased RVSP and aggravated the ratio of right ventricle compared with normoxic rats, which was partially reversible by administration of AZT (TERT inhibitor, 20 mg/kg body weight). Con, control; Hyp, hypoxia; MCT, monocrotaline, A, azidothymidine. *P<0.05 vs. normoxia, #P<0.05 vs. hypoxia, $P<0.05 vs. normoxia, &P<0.05 vs. MCT; n = 5. All values are denoted as mean ± SEM.

To determine whether TERT is involved in the pathogenesis of PH, we then tested the TERT inhibitor AZT in experimental rat models of PH. Hypoxia or MCT injection significantly elevated right ventricular systolic pressure (RVSP) (Figure 3A, B). Consistently, the RV/LV+S ratio was significantly increased in the hypoxia group and MCT group compared with that in the sham-control rats (Figure 3C). However, the TERT inhibitor AZT significantly reversed the augment of RVSP and RV/LV+S ratio induced by hypoxia or MCT (Figure 1D, 1E, 3B, 3C). These results implicate a role of TERT in the development of pulmonary hypertension in experimental rat models.

### The Expression of 15-LO-2 was Regulated by TERT *in vivo* and *in vitro*

**Figure 4:**
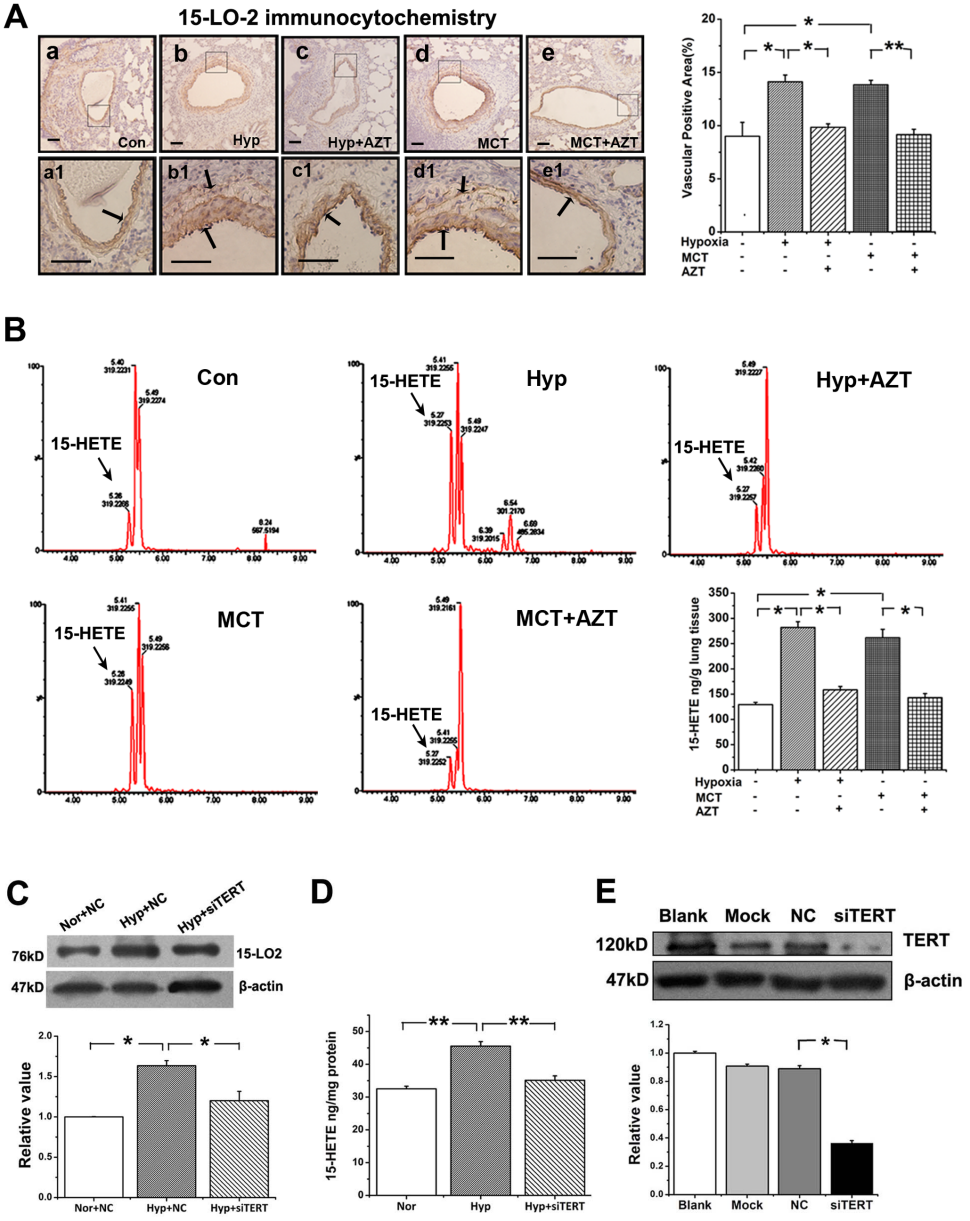
Expression of 15-LO-2 protein and 15-HETE production in PAs and PASMCs. **A**, 15-LO-2 protein expression determined by immunocytochemistry. Framed areas in a–e are shown at high magnification in a1-e1, respectively. Scale bars are 50 µm. Quantitative analyses of positive staining per vascular area (adventitia+media+intima+lumen) showed that hypoxia or MCT injection induced the expression of 15-LO-2, which was deduced by AZT administration (20 mg/kg body weight). **B**, Measurement and identification of endogenous 15-HETE levels by UPLC. UPLC showed that hypoxia or MCT injection induced the generation of endogenous 15-HETE in lung tissues, but administration of AZT to rats decreased the level of endogenous 15-HETE. Con, control; Hyp, Hypoxia; Nor, Normoxia; MCT, monocrotaline; AZT, azidothymidine. **C**, 15-LO2 expression in PASMCs was suppressed by the siRNA of TERT. **D**, Endogenous 15-HETE production in PASMCs was inhibited by knockdown the gene of TERT. Norm indicates normoxia; NC, negative control transfection. (*P<0.05, n = 3). **E**, TERT protein expression was down-regulated after transfecting the TERT siRNA sequence into PASMCs. “Mock” indicates only treating cells with transfection reagent; “NC”, the negative control sequence. (*P<0.05, **P<0.01; n = 6). All of the values are denoted as means ± SEM.

To understand how TERT regulates the pathogenesis of PH, we next measured the expression of 15-LO with TERT inhibition. We semi-quantified the expression of 15-LO-2 in the lung tissue sections of rats at protein levels using histochemistry. Amazingly, TERT inhibition decreased the activation of 15-LO-2 induced by hypoxia or MCT (Figure 4A). Furthermore, we found that the endogenous 15-HETE level was increased in rat lung tissues under hypoxia or with MCT administration, which could be reduced by administration of AZT (Figure 4B).

To demonstrate the role of TERT in the hypoxia-induced 15-LO pathway in PASMCs, we knocked down the gene of TERT (the efficiency of transfection was shown in Figure 4E) and found that the expression of 15-LO-2 and the production of 15-HETE were reduced (Figure 4C, 4D). These results indicate a role of TERT in regulating the 15-LO-2/15-HETE pathways during pulmonary vascular remodeling.

### 15-LO-2/15-HETE had a Positive Feedback on TERT Expression in PAs and PASMCs of Rats

**Figure 5:**
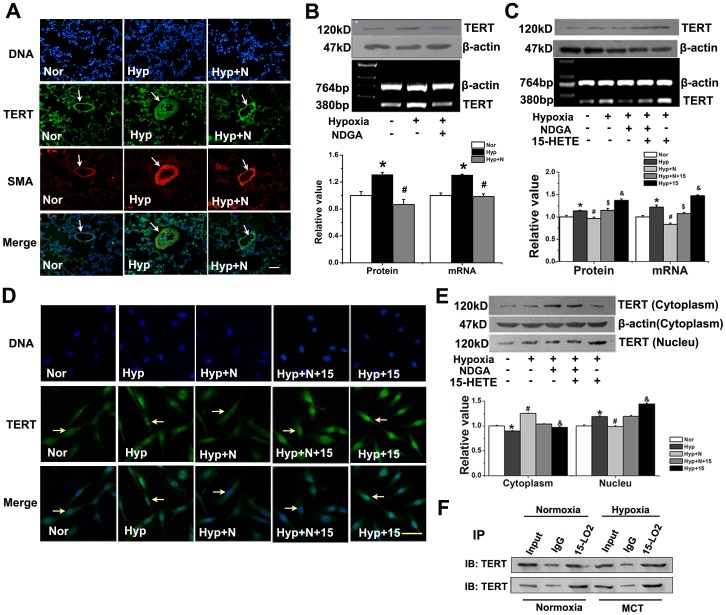
Translocation of TERT protein from cytoplasm to nucleus upon hypoxia and interaction of TERT and 15-LO-2. **A**, Immunofluenrescence of PAs from Normoxia, Hypoxia, Hypoxia with NDGA treated rats. Lung sections were stained with (49,69-diamidino-2-phenylindole (DAPI), blue), TERT (green), α-smooth muscle actin (SMA; red), and Merged (yellow). Scale bars are 50 µm. **B**, The protein and mRNA of TERT was quantified by Western Blot and RT-PCR in rat lung homogenates from normoxia, hypoxia and hypoxia with NDGA rats. **C**, TERT protein and mRNA was examined in PASMCs. **D**, PASMCs were fixed and stained with anti-TERT antibody (green) and DNA counterstaining DAPI (blue) and merged. Scale bars are 20 µm. **E**, Distribution of TERT protein. Nuclear extracts (Nucleu, 20 µg), and cytoplasmic extracts (Cytoplasm, 100 µg) of PASMCs were prepared, and TERT protein was detected by Western Blot. **F**, Co-IP of TERT and 15-LO-2 by in PASMCs. 5% input samples, mouse IgG used as negative control and immunocomplexes obtained with an anti-LO-2 antibody were analyzed by immunoblot (IB) with anti-TERT antibody. *P<0.05 vs. normoxia, #P<0.05 vs. hypoxia, $P<0.05 vs. hypoxia with NDGA or CDC, &P<0.05 vs. hypoxia; n = 5. Nor, normoxia; Hyp, hypoxia; N, NDGA. All values are denoted as means ± SEM.

To determine whether 15-LO-2/15-HETE, in turn, affects the expression of TERT, we used the 15-HETE inhibitor NDGA in rats. In small pulmonary arteries from control lungs, little staining for TERT or SMA in vascular walls was observed by confocal microscopy. In contrast, strong staining for TERT and SMA was observed in small hypertrophied pulmonary arteries from hypoxic rats, which was suppressed by the exogenous 15-HETE inhibitor NDGA (Figure 5A).

We then examined the mRNA and protein levels of TERT after NDGA administration. NDGA suppressed hypoxia-induced TERT expression in PA at both mRNA and protein levels (Figure 5B), suggesting that the hypoxia-induced TERT expression in PA relies on 15-LO-2. Meanwhile, we found that after blocking 15-HETE pathway with NDGA, the activation of hypoxia-induced TERT was suppressed, which can be recovered by adding exogenous 15-HETE (Figure 5C). These results suggest that 15-LO-2/15-HETE positively regulates the expression of TERT induced by hypoxia *in vivo* and *in vitro*. Together with the reciprocal regulation of 15-LO-2 by TERT (Figure 4), this data provides a positive feedback loop between TERT and 15-LO-2.

### 15-HETE Augmented Nuclear Translocation of Hypoxia-induced TERT in PASMCs

Recent reports have implicated that the accumulation of TERT in the nucleus is important in the process of vascular smooth muscle cell (VSMC) proliferation [12]. To ascertain whether 15-HETE mediated the nuclear translocation of TERT, we quantified the amounts of TERT protein in both nuclear and cytoplasmic fractions. Hypoxia significantly mobilized TERT to the nucleus, whereas treatment with NDGA decreased this translocation, which was reversed by the addition of exogenous 15-HETE (Figure 5E). We observed similar phenotypes with TERT immunofluorescence (Figure 5D). These results show that the translocation of TERT from cytoplasm to nucleus upon hypoxia was regulated by 15-LO/15-HETE.

### TERT Interacts with 15-LO-2

Since the TERT inhibitor suppressed the up-regulation of 15-LO-2 and vice versa, we hypothesized that TERT and 15-LO-2 formed a biochemical complex. Indeed, TERT was co-immunoprecipitated with 15-LO-2 (Figure 5F), supporting a biological interaction between TERT and 15-LO-2.

### 15-HETE Compensates the Loss of TERT during PASMC Migration

**Figure 6:**
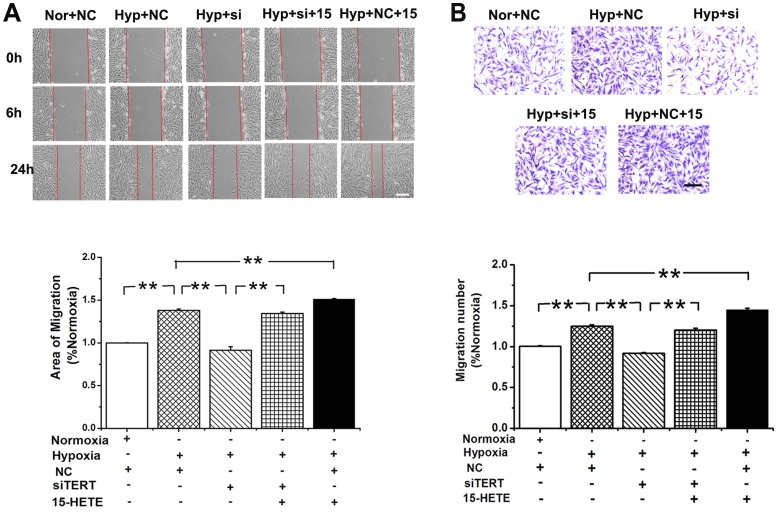
15-HETE rescues TERT depletion caused PASMCs migration defects. **A**, Scratch-wounding cell migration assay. The migration induced by hypoxia was blocked by silencing TERT, which is recovered by exogenous 15-HETE. **B**, Transwell assays coincident with the results from scratch test. Cells were plated in the upper chamber of the filters. At 24 hours after plating, cells that had migrated to the underside of the filters were fixed and stained with crystal violet. Relative cell migration was determined by the number of migrated cells. Scale bars are 50 µm. (*P<0.05; **P<0.01, n = 3). All of the values are denoted as mean ± SEM. Nor, normoxia; Hyp, hypoxia; NC, negative control; si, si-TERT; 15, 15-HETE.

To elucidate whether TERT is involved in PASMC migration, we knocked down TERT with siRNA. In the scratch-wound assay, the migration induced by hypoxia was significantly inhibited in TERT depleted PASMCs, which was reversed by the addition of exogenous 15-HETE (Figure 6A). In addition, we examined the effects of TERT and 15-HETE during PASMC migration to serum in a modified Boyden chamber. Accordingly, the migration of PASMCs induced by hypoxia was significantly abolished by silencing TERT, which could also be reversed by adding exogenous 15-HETE (Figure 6B). These results demonstrate that the 15-HETE/TERT positive feedback loop regulates the migration of PASMCs under hypoxic conditions.

### The Proliferation of PASMCs Restrained by TERT Knock down was Reversed by 15-HETE

**Figure 7:**
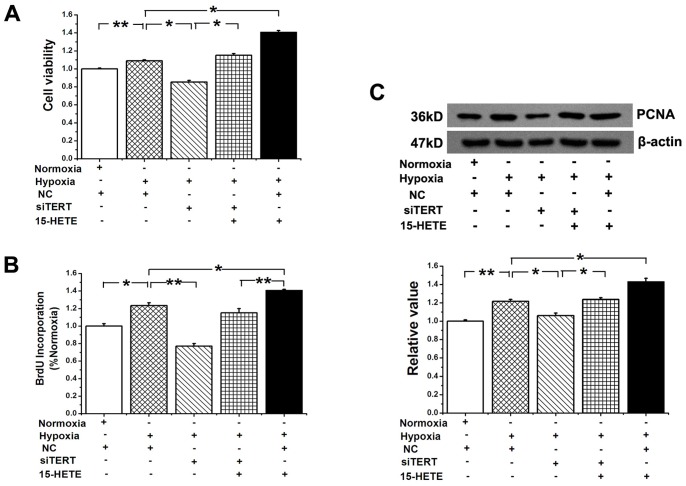
The proliferation of PASMCs restrained by siRNA of TERT was reversed by 15-HETE. **A**, MTT assay showed that the cell viability in hypoxia-induced PASMCs growth. **B**, 5-Bromodeoxyuridine (BrdU) incorporation study demonstrated that hypoxia-induced DNA synthesis was blocked by silencing TERT, which is rescued by exogenous 15-HETE. **C**, PCNA protein expression was determined after knocking down TERT. (*P<0.05, **P<0.01, n = 3). All of the values are denoted as mean ± SEM. Mock indicates only treating cells with transfection reagent. NC, negative control sequence.

To determine the effect of TERT on PASMC proliferation, cell viability was examined by the MTT assay. Silencing the gene of TERT, decreased cell viability induced by hypoxia, which was significantly rescued by exogenous 15-HETE (Figure 7A). We observed similar phenotypes in cell proliferation by using BrdU incorporation assay (Figure 7B) and detecting the expression of the proliferating cell nuclear antigen (PCNA) in PASMCs (Figure 7C). These results indicate that the 15-HETE/TERT positive feedback loop regulates the proliferation of PASMCs under hypoxia.

### PASMC Trans-differentiation is Mediated by TERT and 15-HETE

**Figure 8:**
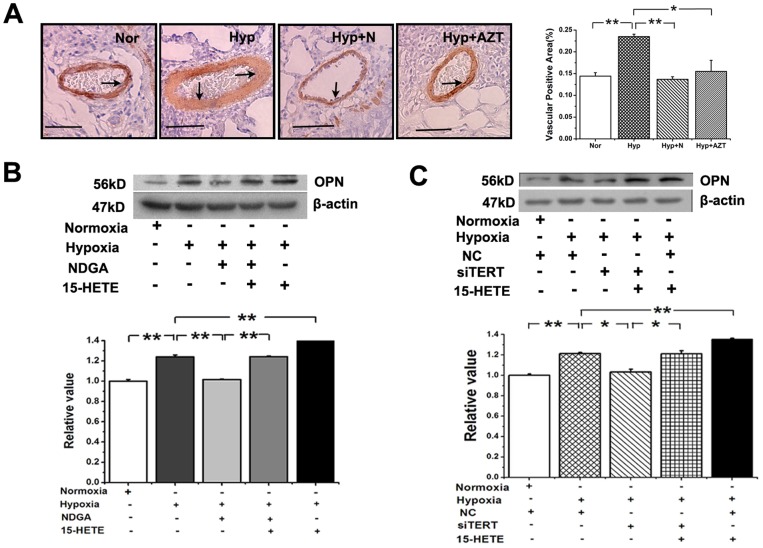
Osteopontin (OPN) expression in PAs and PASMCs. **A**, OPN expression in PAs from normoxia, hypoxia, hypoxia with NDGA and hypoxia with AZT. Scale bars are 50 µm. Quantitative analyses of positive staining per vascular area (adventitia+media+intima+lumen). **B**, OPN expression in PASMCs. Hypoxia induced expression was suppressed by 15-HETE inhibitor NDGA and enhanced by exogenous 15-HETE. **C**, OPN expression induced by hypoxia was restrained by siRNA of TERT and reversed by exogenous 15-HETE. (*P<0.05; **P<0.01, n = 3). All of the values are denoted as mean ± SEM. Nor, normoxia; Hyp, hypoxia; AZT, azidothymidine; N, NDGA; NC, negative control.

To ascertain whether 15-HETE or TERT participates in the phenotypic modulation of PASMCs, we treated the rats with NDGA or AZT and measured the expression of OPN in the pulmonary vessels by histochemistry. Osteopontin (OPN) expression was upregulated by hypoxia, which was inhibited by either the 15-HETE inhibitor or the TERT inhibitor (Figure 8A). As for PASMCs, the expression of OPN was decreased after inhibiting endogenous 15-HETE with NDGA while recovered to the similar level of hypoxic group by addition of exogenous 15-HETE (Figure 8B). Furthermore, TERT knock-down attenuated the expression of OPN which was rescued by 15-HETE (Figure 8C). These results suggested that the 15-HETE/TERT positive feedback loop regulates the induction of OPN under hypoxia.

### TERT Participated in the Cell-cycle Transition from G_0_/G_1_ Phase to S Phase in PASMCs, which was Forward Catalyzed by 15-HETE

**Figure 9:**
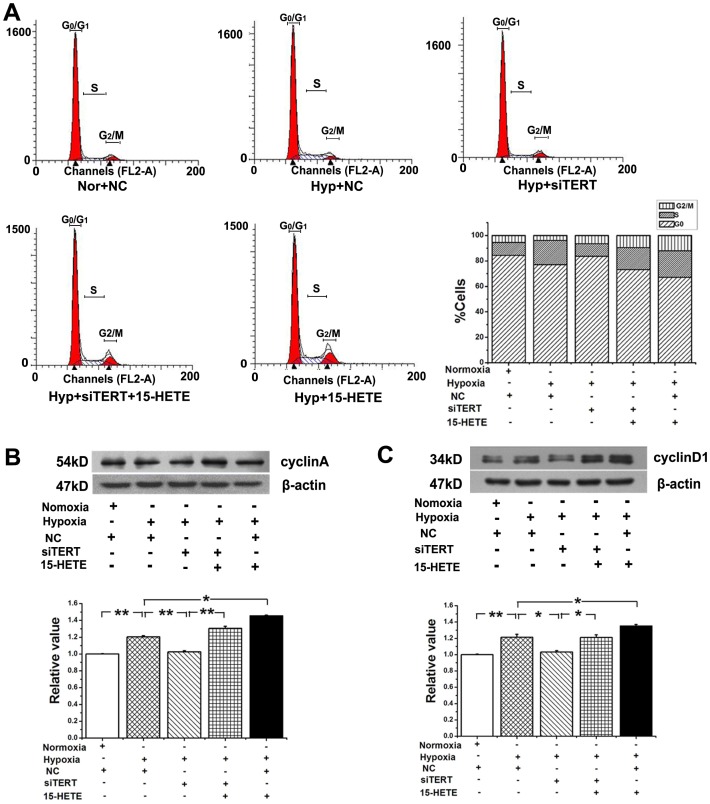
TERT participated in the cell-cycle distribution of PASMCs. **A**, The number of cells in each phase of the cell cycle was examined by FACS analysis (n = 2). The results showed that inhibiting TERT reduced the percentage of cells in S phase accompanied with a concomitant increase of cells at G_0_/G_1_ phase. The expression of cyclin A (**B**) and cylinD1 (**C**) of PASMCs was inhibited by siRNA of TERT, which was enhanced by 15-HETE. (*P<0.05, **P<0.01, n = 3). Nor, normoxia; Hyp, hypoxia. All of the values are denoted as mean ± SEM.

Hypoxia induced cell proliferation is demonstrated by an increased percentage of cells entering S-phase. The amount of S-phase cells was significantly decreased and the percentage of G_0_/G_1_-phase cells was increased in TERT siRNA-treated hypoxic PASMCs. Compared with cells treated with the control siRNA, the percentage of cells in S phase was decreased by 9.12%, accompanied with a concomitant increase of cells in the G_0_/G_1_ phase from 77.08% to 83.66% (Figure 9A). Since cyclin A and cyclin D1 play important roles in both the S and G_2_/M phases [29], we analyzed the posttranslational levels of cyclin A and cyclin D1 in PASMCs. A significant reduction in the expression of cyclin A and cyclin D1 was observed after knocking down TERT, but the effects were reversed by exogenous 15-HETE administration (Figure 9B, 9C). These data showed that the 15-HETE/TERT positive feedback was involved in the cell cycle control of PASMCs.

## Discussion

To our knowledge, we have demonstrated that the expression of telomerase reverse transcriptase (TERT) is enhanced in the pulmonary vasculature of pulmonary hypertension (PH) patients and experimental models of PH for the first time. Meanwhile, we have proved that TERT inhibition by the reverse transcriptase inhibitor azidothymidine (AZT) attenuates the increased right ventricular systolic pressure (RVSP), the thickening of medial walls and the accumulation of collagen in adventitia, which are characteristic in pulmonary vascular remodeling (PVR) induced by hypoxia or monocrotaline (MCT). Mechanistically, 15-LO-2 and TERT form a biological complex. Importantly, we postulate that TERT and 15-LO-2/15-HETE form a positive feedback loop that regulates PVR and cell-cycle transition from G_0_/G_1_ phase to S phase under hypoxia, providing a basic mechanism underlying the development of PH.

Previous researches show that TERT is expressed at low levels or not detectable in normal somatic cells and tissues while its expression is up-regulated in most carcinoma cells and highly proliferative organs [14], [15], [30], [31], [32]. We find that TERT is highly expressed in the pulmonary vascular walls in both human PH patients and experimental rat PH models, the expression of TERT induced by hypoxia or MCT administration in lung vessels is related to the elevated RVSP and aggravated PVR associated with PH. Moreover, the data here show that TERT inhibition by administrating a chemical inhibitor prevents and reverses the experimental PH, consistent with the previous report that TERT null mice develop less severe hypoxia-induced PH than WT mice [33].

Our studies indicate that: 1, TERT inhibition leads to the down-regulation of 15-LO/15-HETE (Figure 4); 2, 15-LO/15-HETE inhibition results in the reduced expression of TERT (Figure 5A, 5B, 5C); 3, exogenous 15-HETE induces the increased activity of TERT (Figure 5C); and 4, there is an interaction between the protein of TERT and 15-LO-2 (Figure 5F). Therefore, the relationship between TERT and 15-LO-2/15-HETE is interoperable and mutually simulative. We think that the mutual promotion of TERT and 15-LO-2 only occurs during the pathological changes of PH because their activities were increased in the PH patients or PH rat models [22]. We suggest TERT and 15-LO-2 as the potential targets for rational drugs design or computer drug design for PH therapy.

The significant implication of our results is that TERT-dependent increase in 15-LO-2/15-HETE under hypoxic condition, together with the ability of 15-LO-2/15-HETE to stimulate TERT activation, creates a positive-feedback loop. This loop would promote both TERT activation and 15-HETE production, thus generating progressive PVR. Similar positive feedback loop may be present in other perpetual, progressive vascular remodeling events such as during the development of chronic obstructive pulmonary diseases (COPD). The significance of TERT in COPD is also supported by the observation that the process of COPD perpetuates in TERT null mice [34].

TERT activity is tightly regulated by protein translocation from the cytoplasm into the nucleus [35]. One conjecture is that TERT is imported in an inactive form and subsequently assembled into an active enzyme [36], by p23 and hsp90 [37], whereas the underlying mechanism of the translocation of TERT is not clear. A previous study reported that hypoxia induced the reduction of 15-LO in the cytosol [20]. Our data showed that TERT in the cytoplasm was also decreased when exposed to hypoxia while it was increased in the nucleus (Figure 5E). It is possible that 15-LO-2 and TERT form a complex (Figure 5F) and are translocated together. In addition, the decreased TERT protein level in the cytoplasm is inhibited by the 15-LO inhibitor NDGA (Figure 5E). Consistently, the TERT protein translocation into the nucleus upon hypoxia was inhibited by NDGA, which was rescued by 15-HETE (Figure 5D, 5E). Although we have proved the interaction between TERT and 15-LO-2, the detailed characterization of this interaction is still lacking. Whether it is a direct or indirect interaction, or whether there is participation of other signal pathways, will be a focus of our future studies.

Vascular smooth muscle cells (VSMCs) trans-differentiation, or their switch from a contractile/quiescent to a synthetic/secretory/migratory state, is known to play an important role in the pathological vascular remodeling [38]. Synthetic VSMCs secrete large quantities of extracellular matrix proteins, such as osteopontin (OPN), an endogenous modulator of the constitutively activated phenotype of pulmonary adventitial fibroblasts in hypoxic PH [39]. OPN increases during hypoxia-induced proliferation of cultured PASMCs [40]. It is noteworthy in our study that 15-HETE augments the hypoxia induced OPN expression, which is inhibited by silencing TERT. Thus, our research provides a new piece of evidence in the trans-differentiation of PASMCs during pulmonary circulation.

The existing evidence supports a link between the telomerase activity and the progression of cell cycle through the DNA replication phase (S phase) [41]. The telomerase dependent cell cycle regulation could occur at the level of subcellular trafficking. TERT moves from separate sites to telomeres specifically during S phase [42], [43]. Previous studies report that cyclin D1 overexpression is associated with high telomerase levels [44], and cell-cycle progression from the first gap phase (G_1_ phase) to the S phase in mammalian cells is regulated by the accumulation of cyclin A and cyclin D1 [45]. We find that 15-HETE and TERT promotes the cell transition from G_0_/G_1_ phase to S phase and increases the hypoxia induced expression of cyclin A and cyclin D1. Our results indicate that the circuit of TERT-15-LO-2/15-HETE plays a key role of cell cycle regulation in the PASMCs.

**Figure 10:**
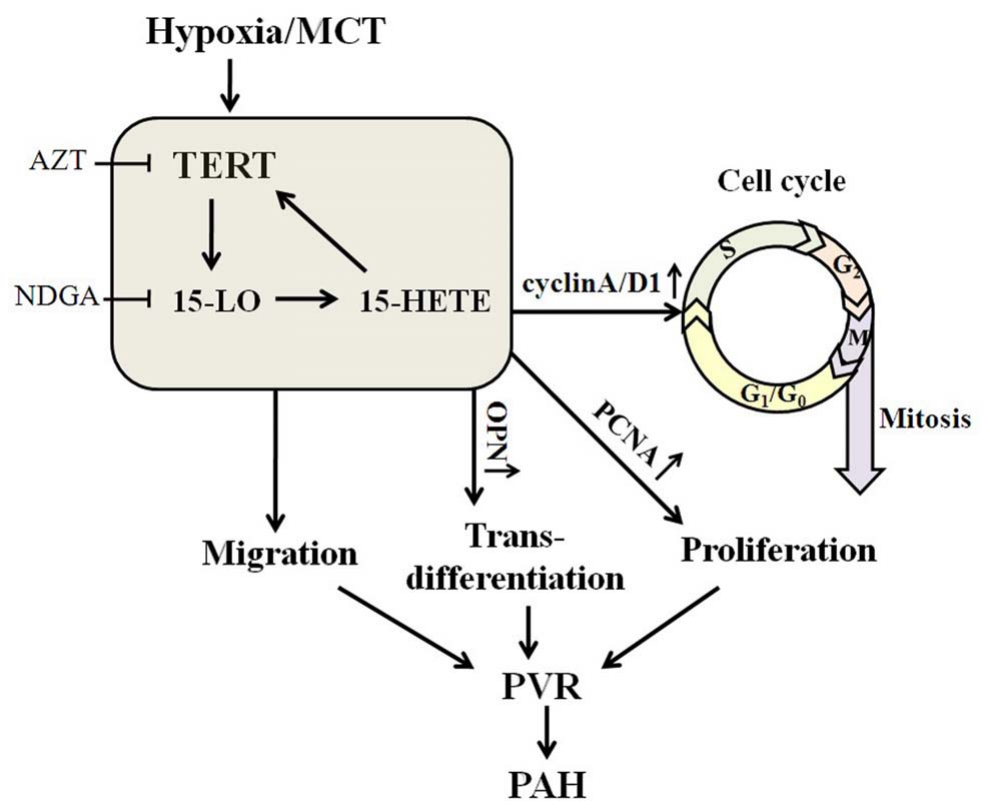
Scheme of the possible cascade of event which involved TERT and 15-LO-2/15-HETE. TERT inhibition by AZT results in the down-regulation of 15-LO/15-HETE, 15-LO/15-HETE inhibition by NDGA leads to the reduced expression of TERT, moreover, exogenous 15-HETE induces the increased activity of TERT and there is an interaction between the protein of TERT and 15-LO-2. The positive-feedback loop between TERT and 15-LO/15-HETE and their mutual effect is engaged in the progression of PASMCs proliferation, migration, trans-differentiation and cell cycle distribution, and participated in pulmonary vascular remodeling associated with PH.

In conclusion, we have discovered a positive-feedback loop between TERT and 15-LO/15-HETE, which is engaged in the progression of PASMCs proliferation, migration, trans-differentiation and cell cycle distribution, and implicated in pulmonary vascular remodeling associated with PH (Figure 10). Strategies that inhibit vascular TERT and 15-LO-2 expression of the lung may antagonize the loop and ameliorate the morbidity and mortality in patients with pulmonary hypertension.
