# Correction: Positive Feedback-Loop of Telomerase Reverse Transcriptase and 15-Lipoxygenase-2 Promotes Pulmonary Hypertension

**DOI:** 10.1371/journal.pone.0308871

**Published:** 2024-08-08

**Authors:** Tingting Shen, Jun Ma, Lei Zhang, Xiufeng Yu, Mengmeng Liu, Yunlong Hou, Yanyan Wang, Cui Ma, Shuzhen Li, Daling Zhu

After publication of this article [1], concerns were raised about Fig 2. Specifically, in Fig 2A, the Control and MCT+AZT panels appear to show the same field of view.

The first and corresponding authors stated that this duplication was the result of a figure preparation error. An updated version of Fig 2 is provided here in which the images have been corrected.

The replacement images provided for Fig 2 show demonstrably less purple staining than the originally published panels. The authors clarified that, during preparation of the original images, they had adjusted the saturation with the intention of making the brown stain more visible. In the updated Fig 2, they have presented the original, unadjusted images.

During editorial follow-up, additional concerns were raised regarding the cytological quantification of TERT. In response, the first and corresponding authors provided the following explanations:

- The TERT-positive foci were quantified over the cross-sectional area of the same part in the lung vessels, and ImageJ was used to quantitatively detect the ratio of TERT-positive area to vascular cross-sectional area.
- The original contrasting conditions were used in the quantification. One contrast was used for all measurements in the same group.
- The arrows in Fig 2A point to the TERT-positive stain. The authors performed an edge trimmer to cut tissue sections of the same thickness (8μm) for immunohistochemical staining and then used a microscope to photograph the cross-section of the tissue. Accordingly, they counted the staining intensity per unit area, not the staining intensity per unit volume.
- Data quantification was carried out in ImageJ.
- The control and experimental datasets were normalized by subtracting the background color from each image.

Underlying image data supporting Fig 2A are available in S1 File. The original individual-level quantitative data from which Fig 2B was generated are available in S2 File.

A member of the *PLOS ONE* Editorial Board reviewed the updated Fig 2, the underlying data, and the additional methodological details provided by the authors, and was satisfied that the raw data support the published results.

The authors apologize for the errors in the published article.

**Fig 2:**
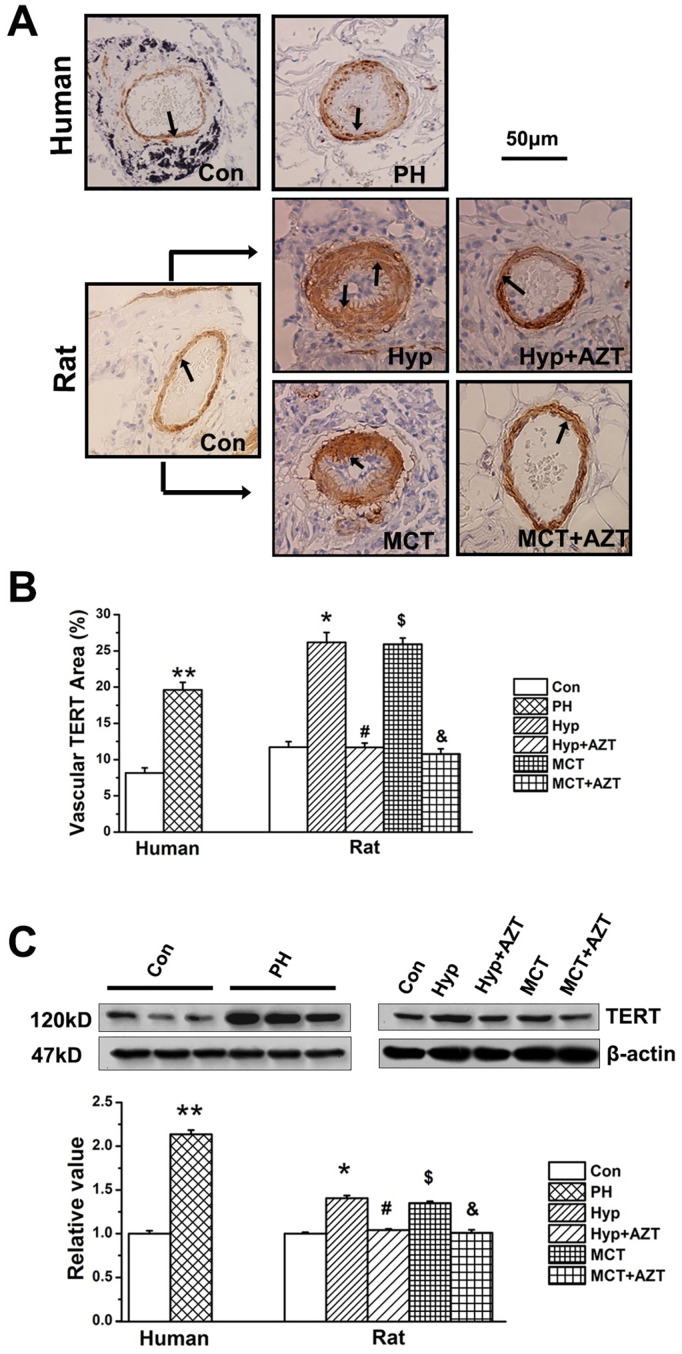
TERT expression increased in PH patients. **A**, TERT protein staining. TERT protein level was increased in the pulmonary vascular wall in PH patients (n = 5). In the animal experiments (n = 6), hypoxia or MCT administration significantly increased the expression of TERT protein compared with control rats, which were reversible by administration of azidothymidine (AZT, 20 mg/kg body weight). **B**, Quantitative analyses of positive staining per vascular area (adventitia+media+intima+lumen). **C**, TERT protein in lung tissues. Con, control; Hyp, hypoxia; MCT, monocrotaline, AZT, azidothymidine. (**P<0.05 vs. control patients, *P<0.05 vs. normoxia, #P<0.05 vs. hypoxia, $P<0.05 vs. normoxia, &P<0.05 vs. MCT). The PAs in each group were obtained from three independent rats and all values are denoted as mean ± SEM.

## Supporting information

S1 File Underlying image data supporting Fig 2A. (ZIP)

S2 File Individual-level quantitative data supporting Fig 2B. (XLSX)
