# Supplementary figures and images for: Correction: Positive Feedback-Loop of Telomerase Reverse Transcriptase and 15-Lipoxygenase-2 Promotes Pulmonary Hypertension
Source: PLoS One. 2024 Aug 8;19(8):e0308871. doi: 10.1371/journal.pone.0308871 (PMC11309466; doi:10.1371/journal.pone.0308871)

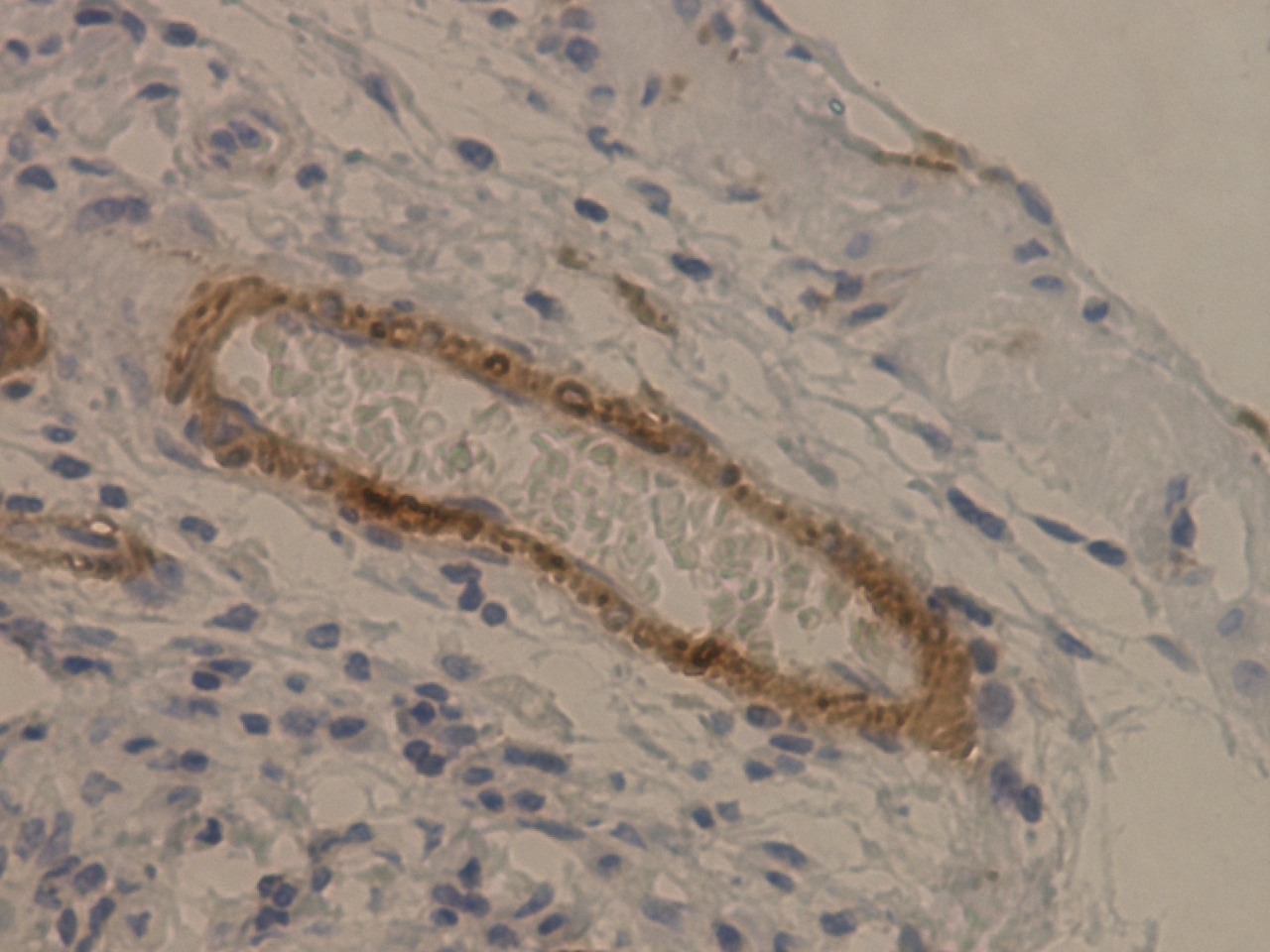

Supplement: S1 File — (ZIP) [file pone.0308871.s001.zip › TERT in Fig 2A/Rat-M+A6.tif]

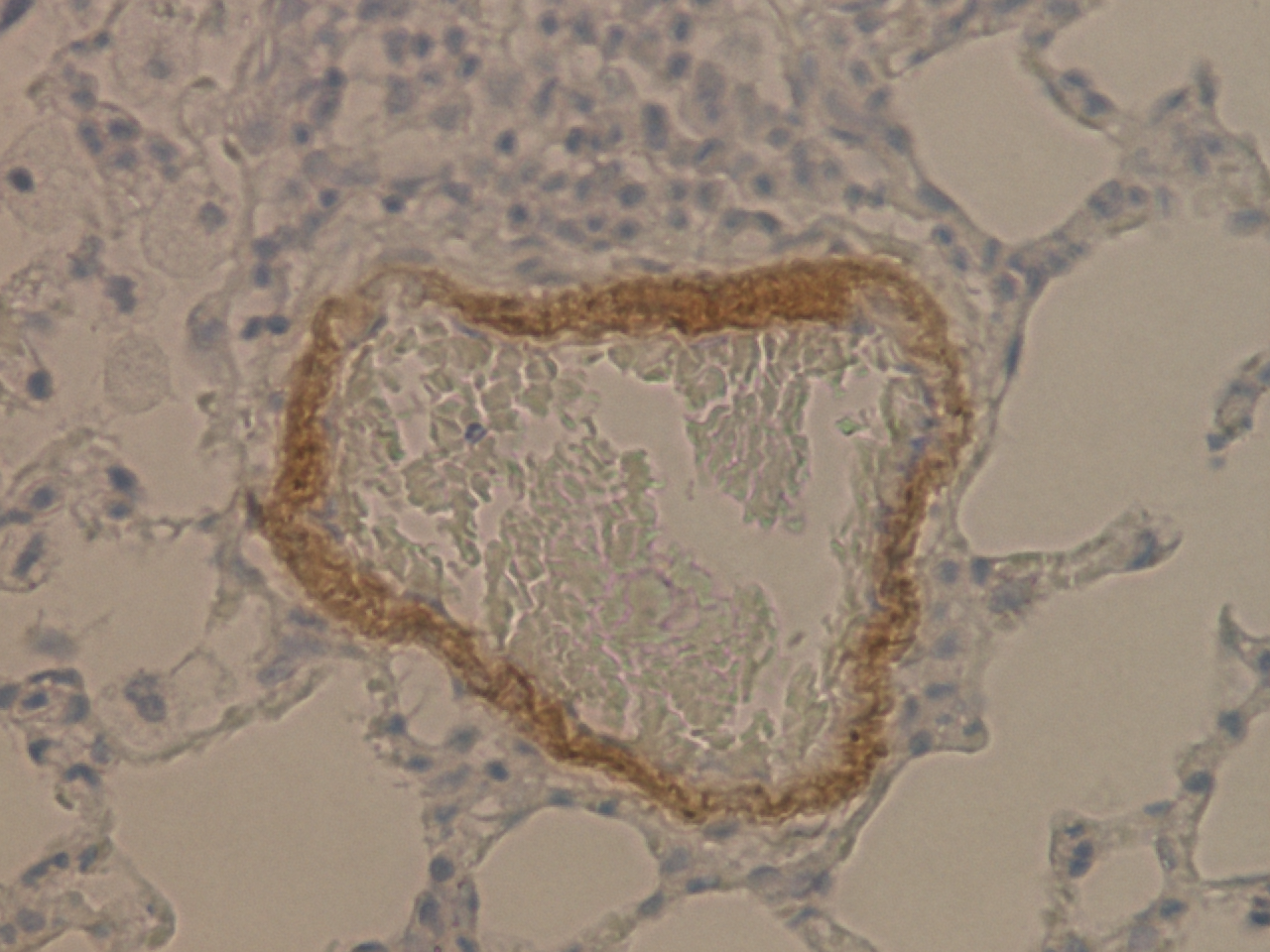

Supplement: S1 File — (ZIP) [file pone.0308871.s001.zip › TERT in Fig 2A/Rat-M+A4.tif]

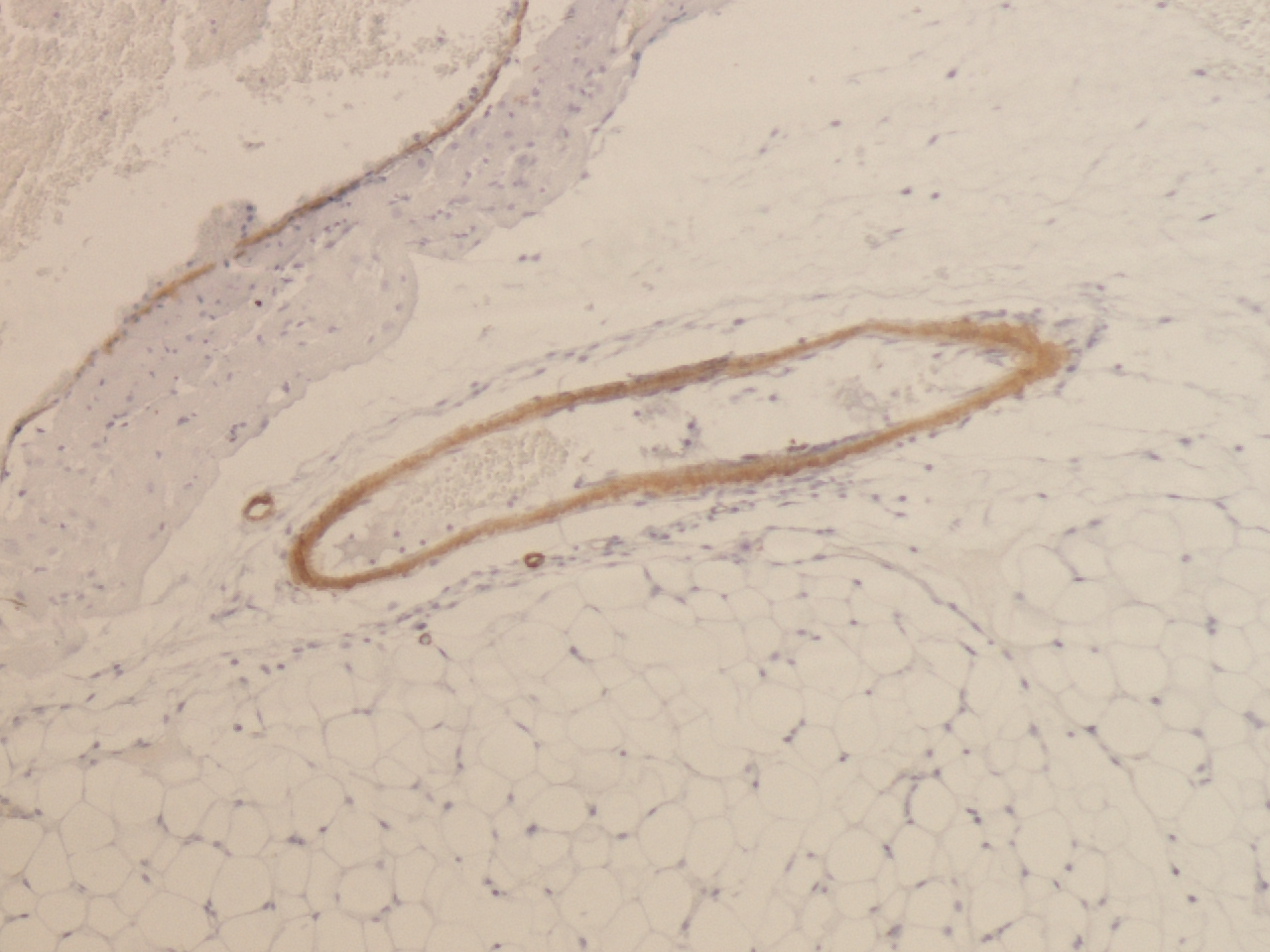

Supplement: S1 File — (ZIP) [file pone.0308871.s001.zip › TERT in Fig 2A/Rat-M+A5.tif]

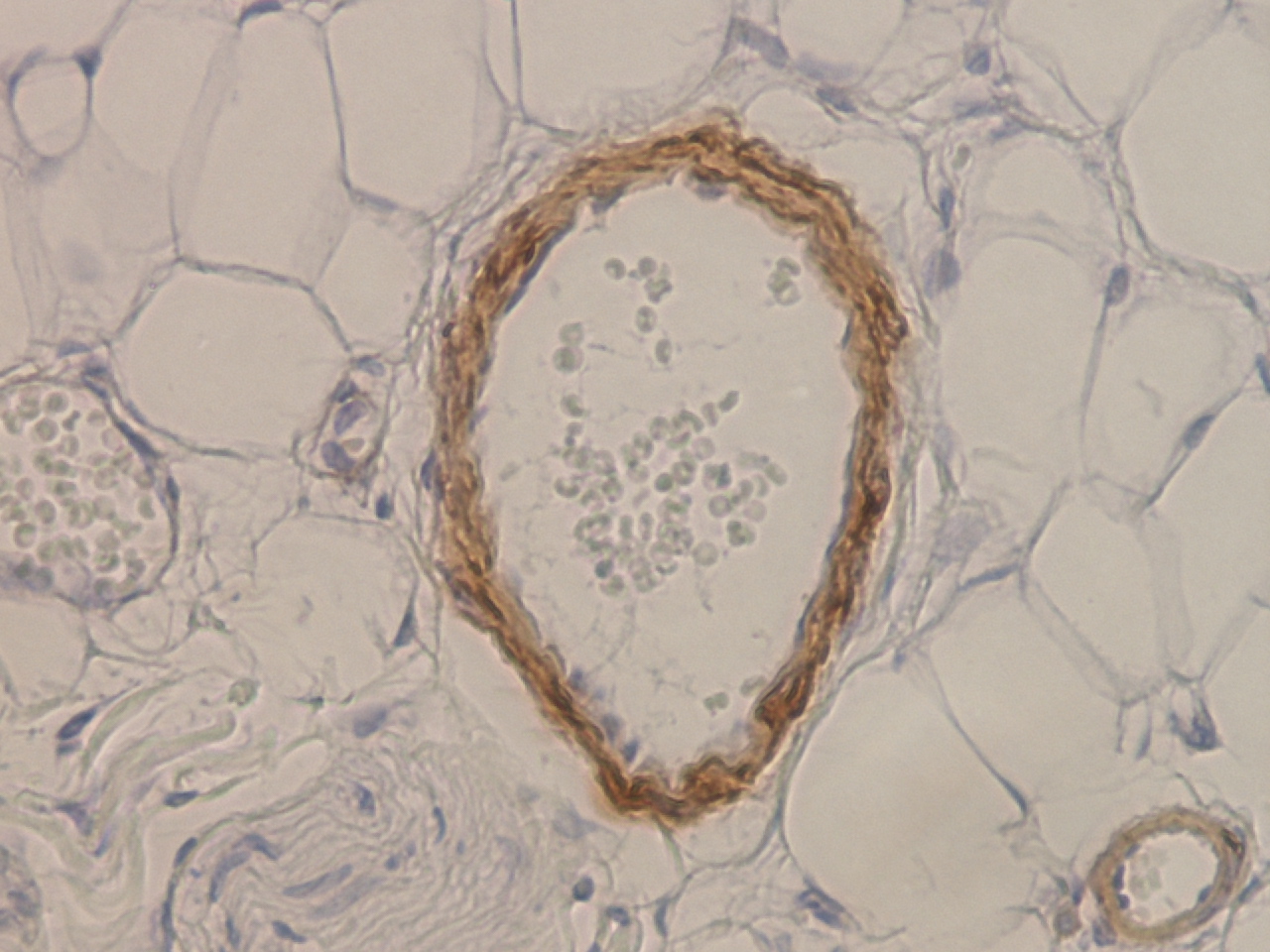

Supplement: S1 File — (ZIP) [file pone.0308871.s001.zip › TERT in Fig 2A/Rat-M+A1.tif]

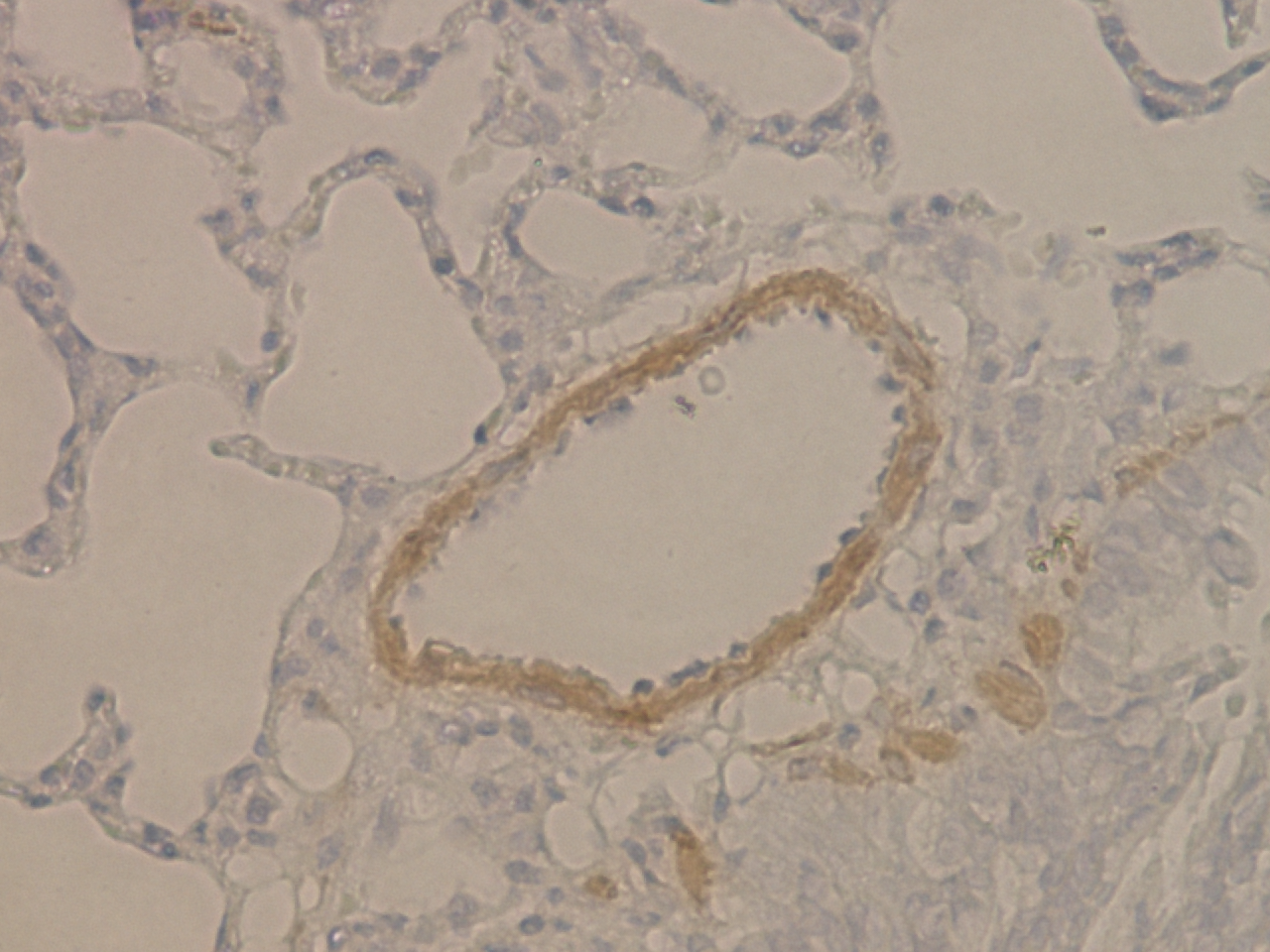

Supplement: S1 File — (ZIP) [file pone.0308871.s001.zip › TERT in Fig 2A/Rat-M+A2.tif]

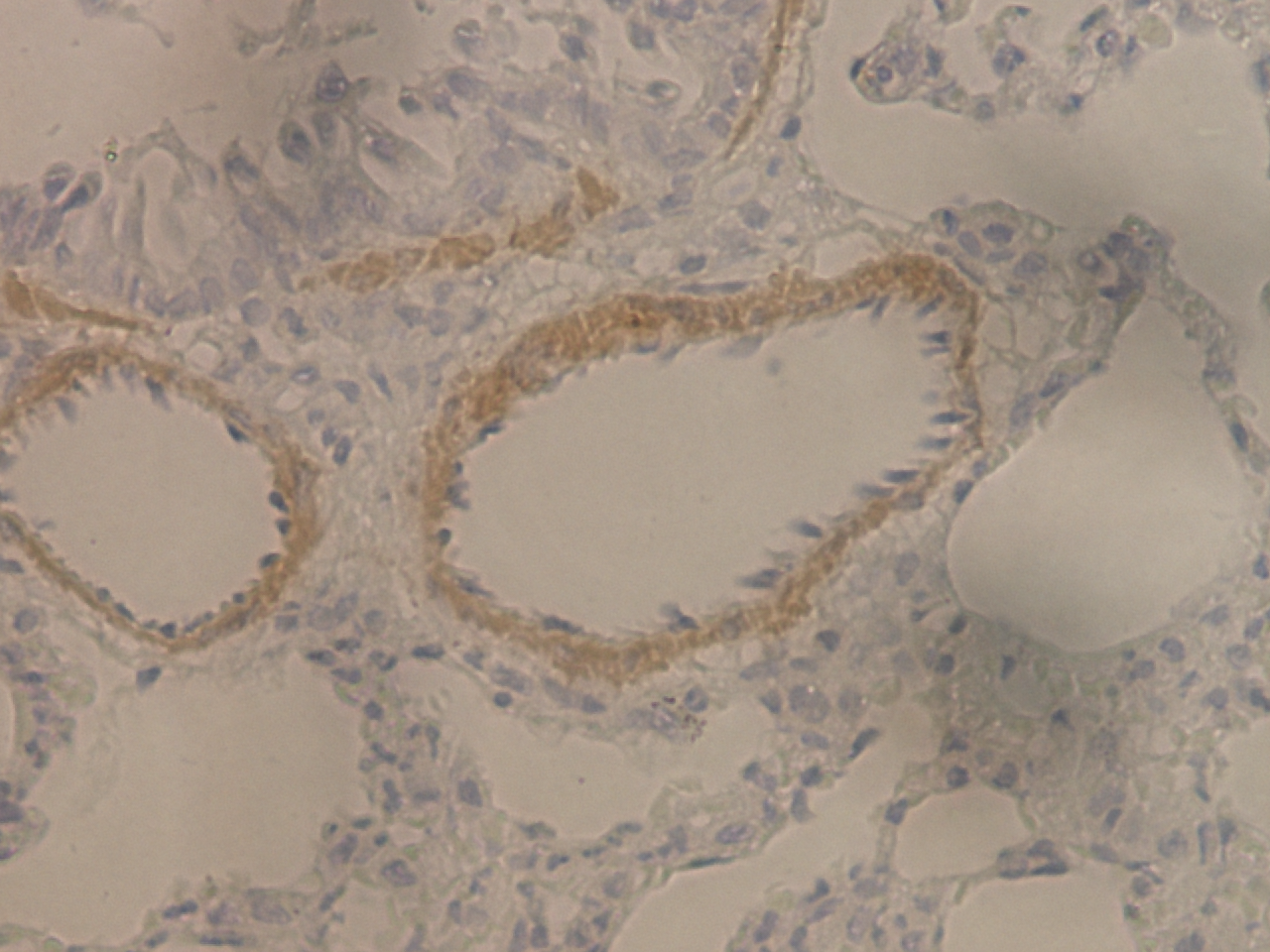

Supplement: S1 File — (ZIP) [file pone.0308871.s001.zip › TERT in Fig 2A/Rat-M+A3.tif]

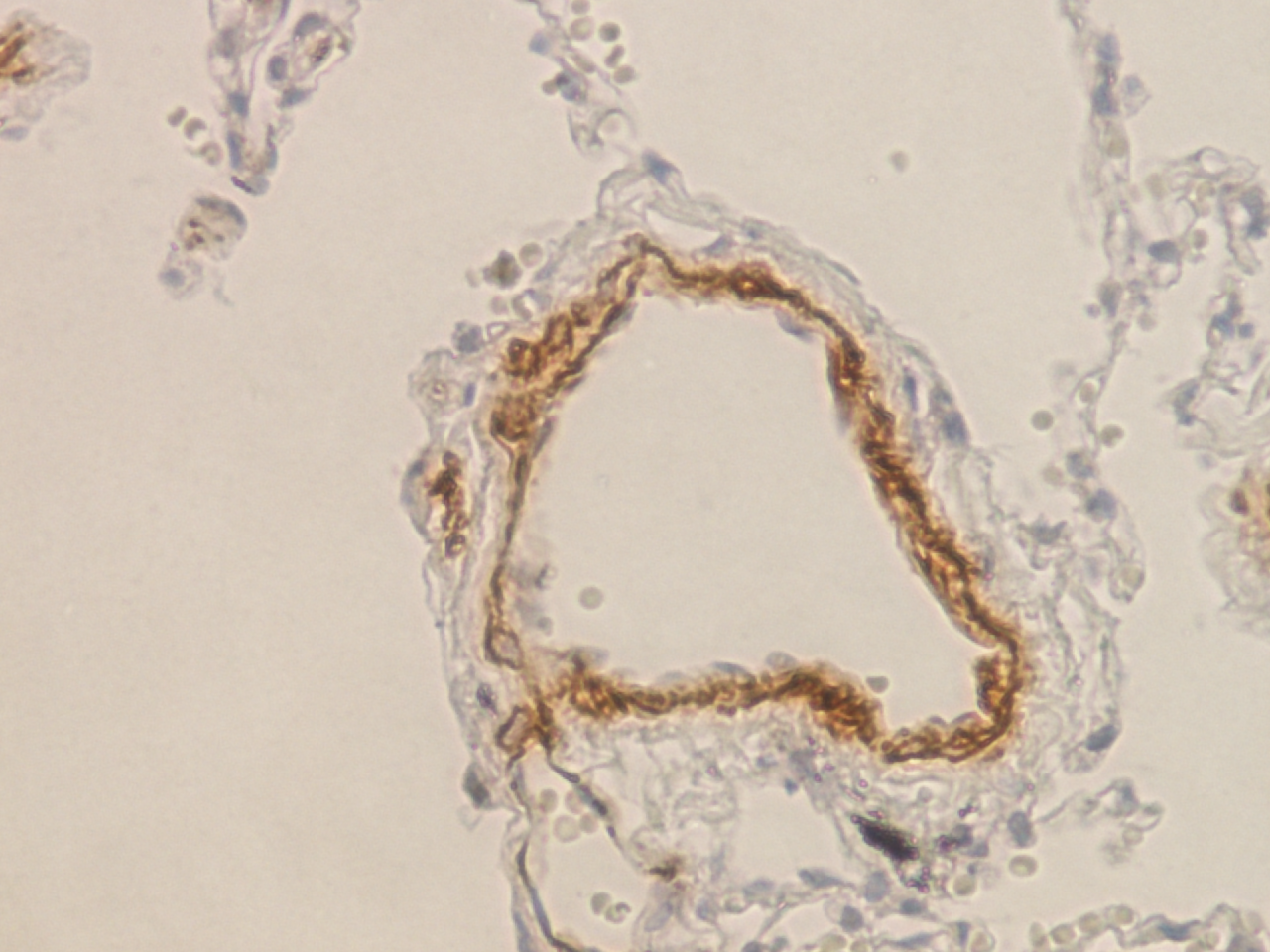

Supplement: S1 File — (ZIP) [file pone.0308871.s001.zip › TERT in Fig 2A/Human Con5.tif]

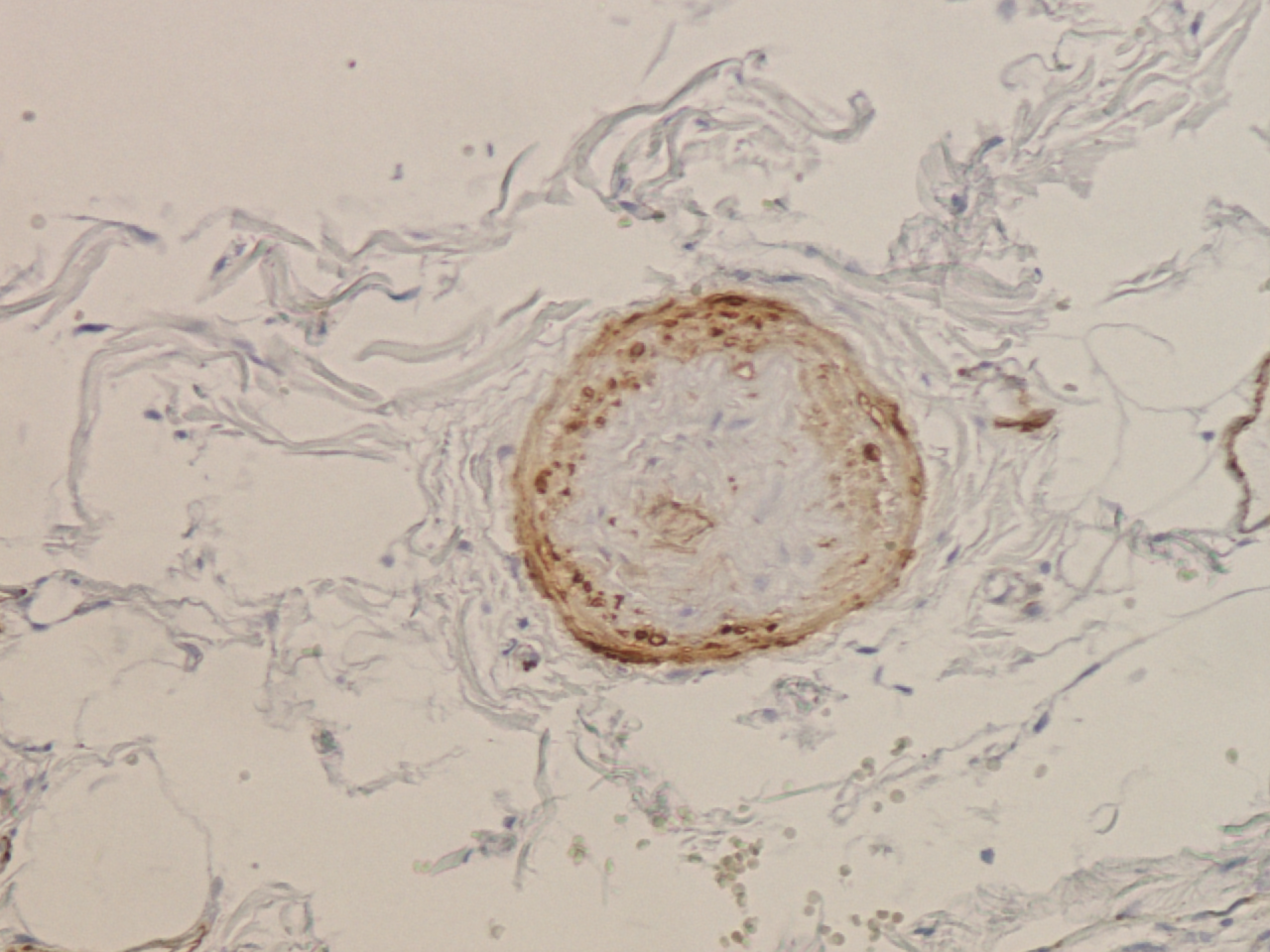

Supplement: S1 File — (ZIP) [file pone.0308871.s001.zip › TERT in Fig 2A/Human PH1.tif]

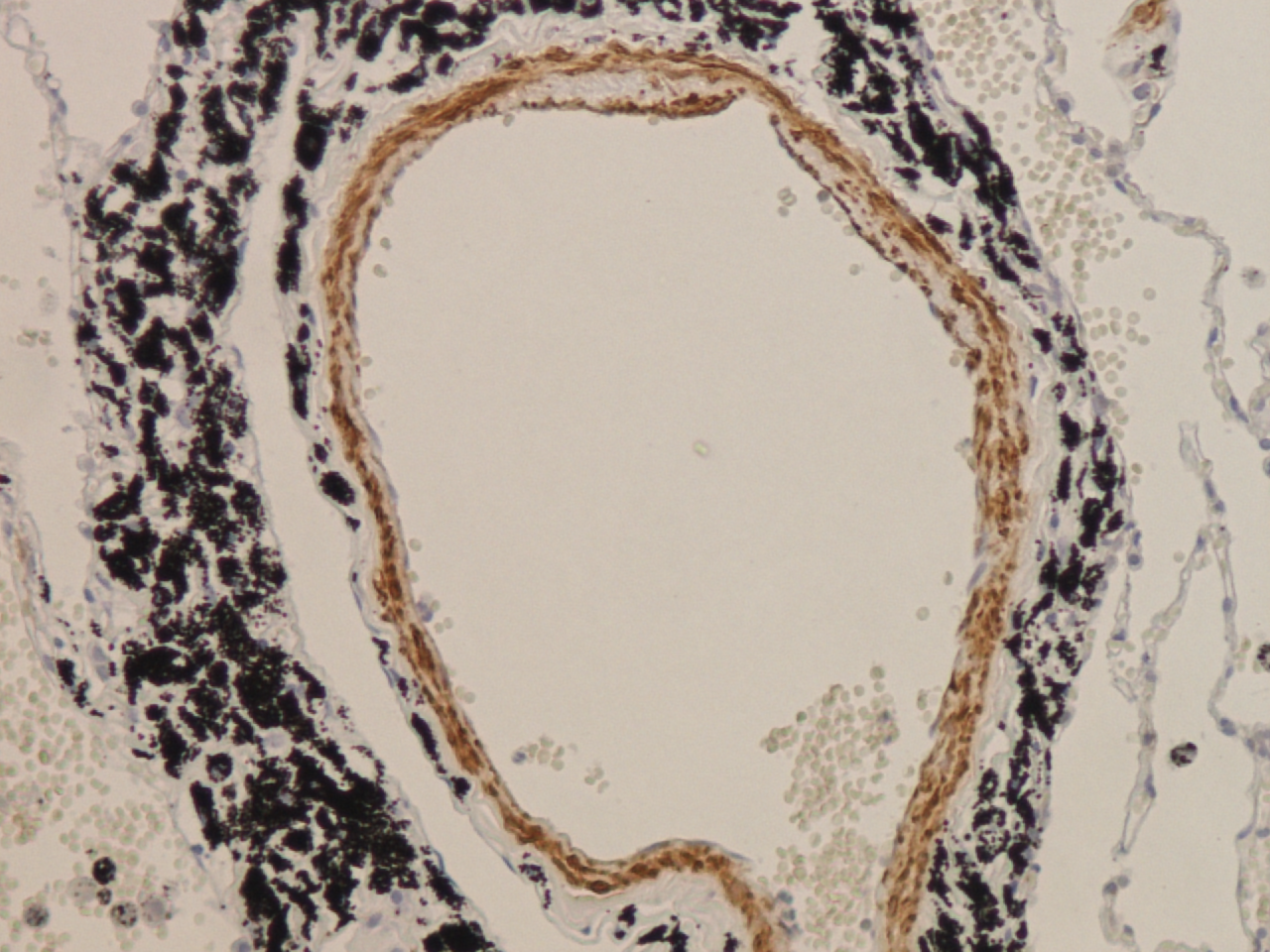

Supplement: S1 File — (ZIP) [file pone.0308871.s001.zip › TERT in Fig 2A/Human Con4.tif]

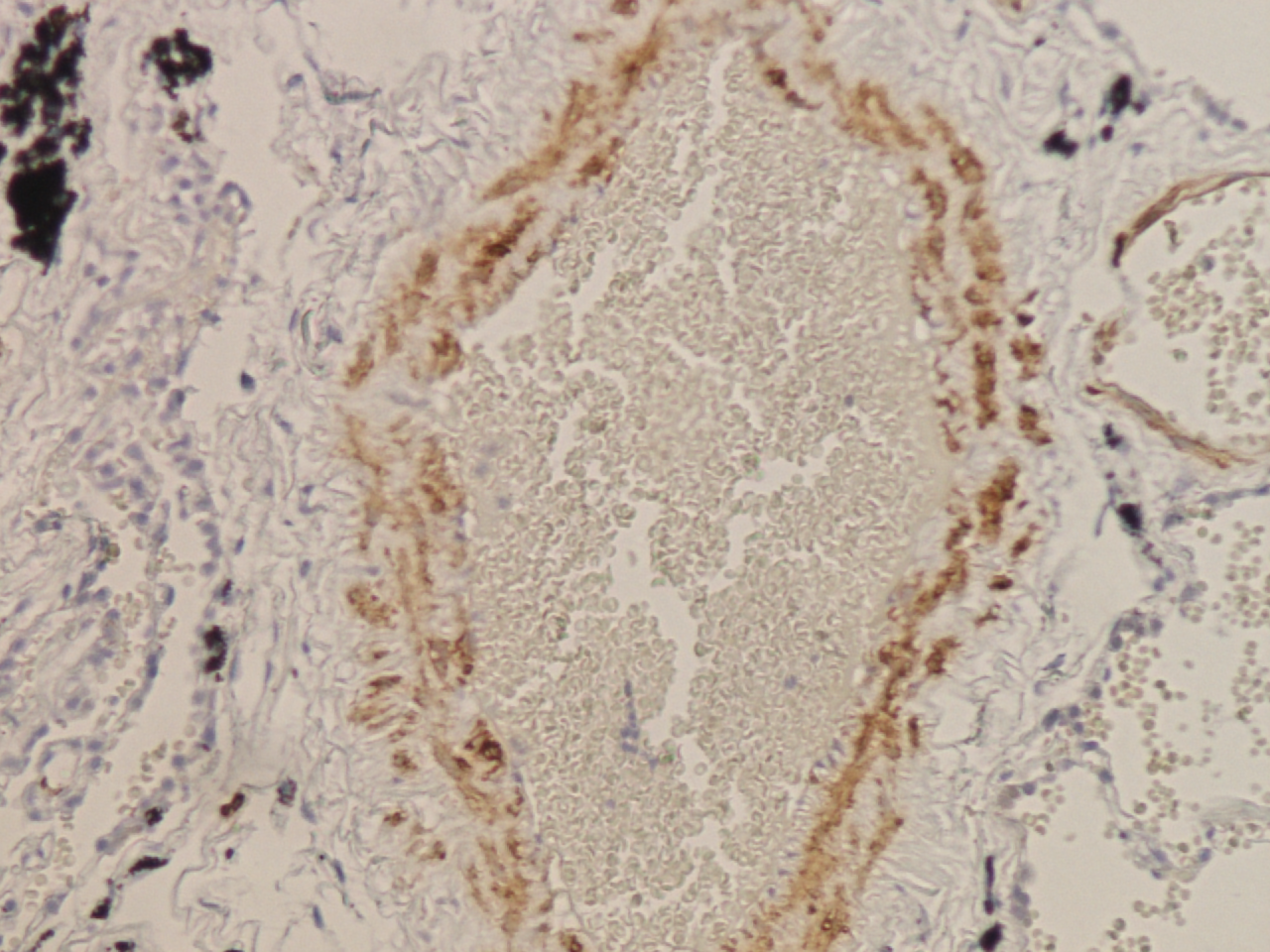

Supplement: S1 File — (ZIP) [file pone.0308871.s001.zip › TERT in Fig 2A/Human PH3.tif]

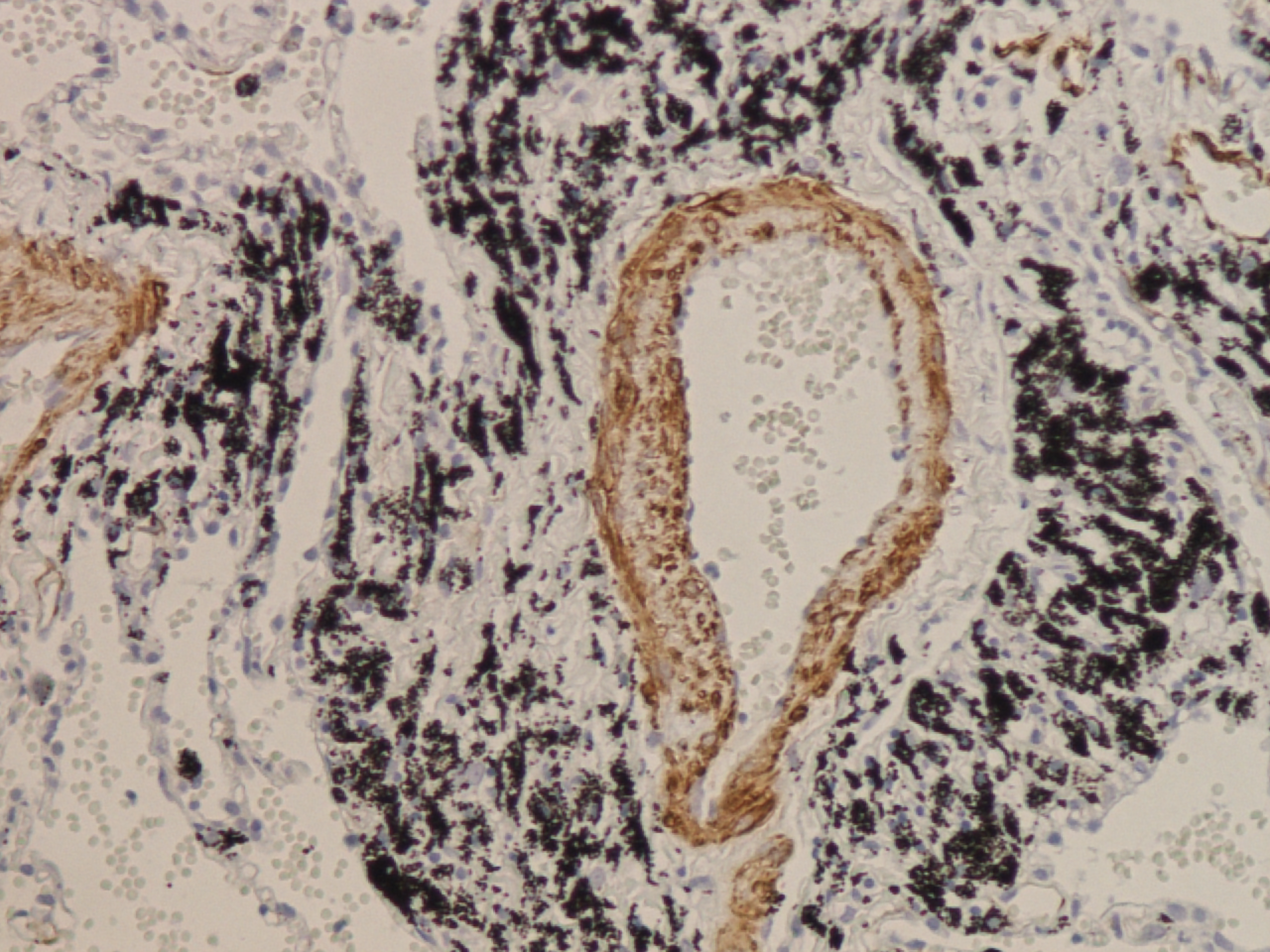

Supplement: S1 File — (ZIP) [file pone.0308871.s001.zip › TERT in Fig 2A/Human PH2.tif]

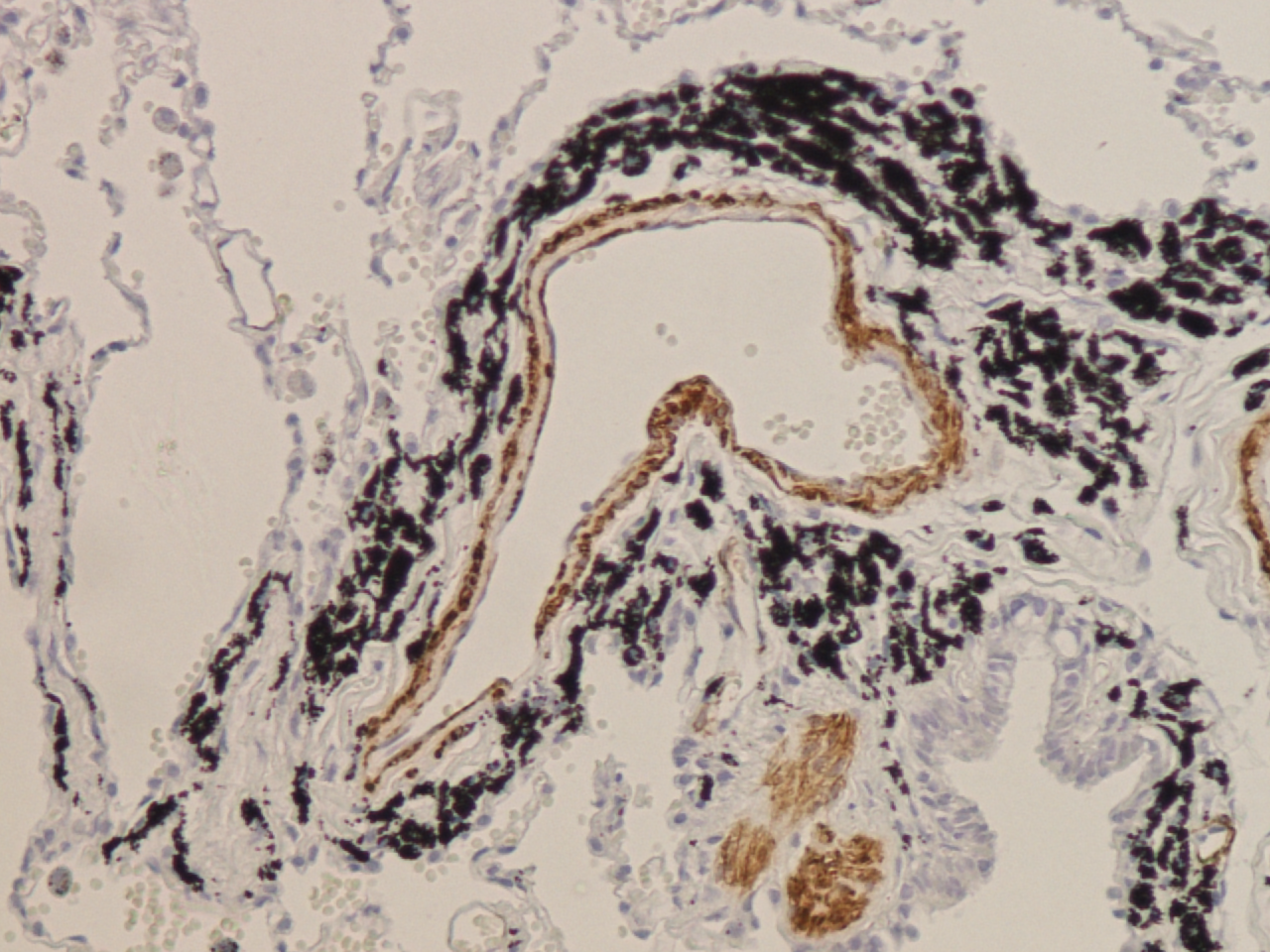

Supplement: S1 File — (ZIP) [file pone.0308871.s001.zip › TERT in Fig 2A/Human Con3.tif]

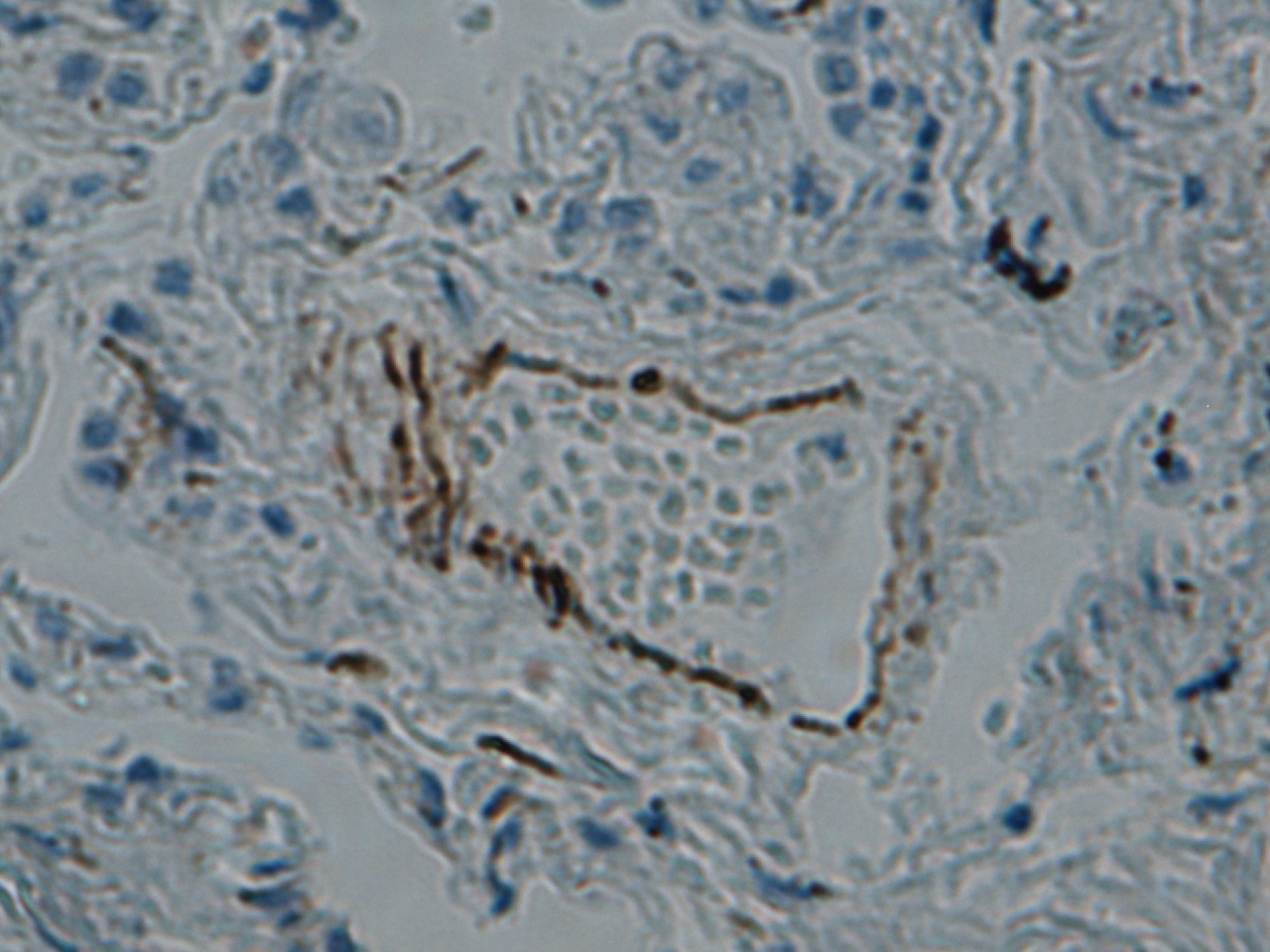

Supplement: S1 File — (ZIP) [file pone.0308871.s001.zip › TERT in Fig 2A/Human Con2.tif]

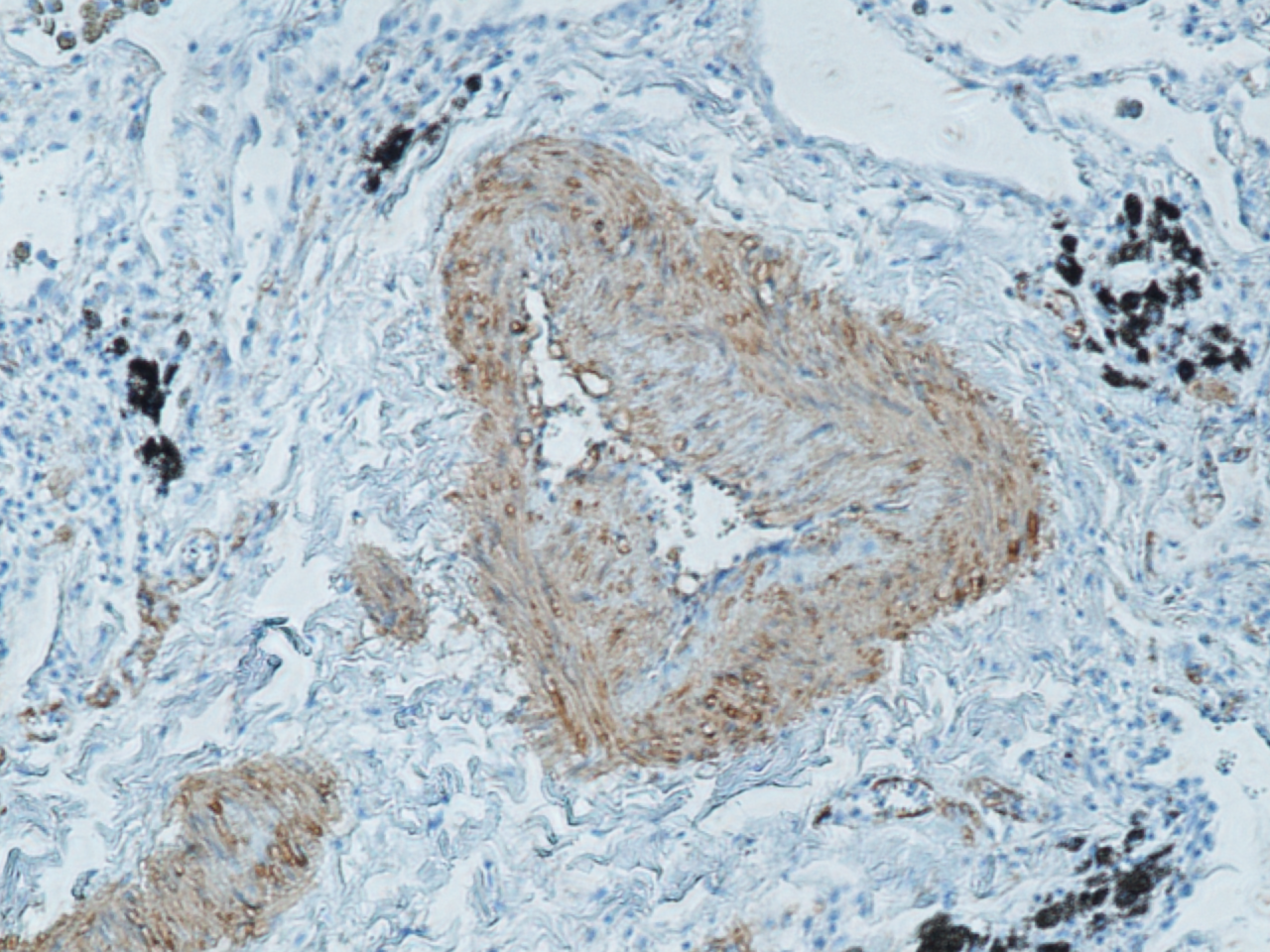

Supplement: S1 File — (ZIP) [file pone.0308871.s001.zip › TERT in Fig 2A/Human PH5.tif]

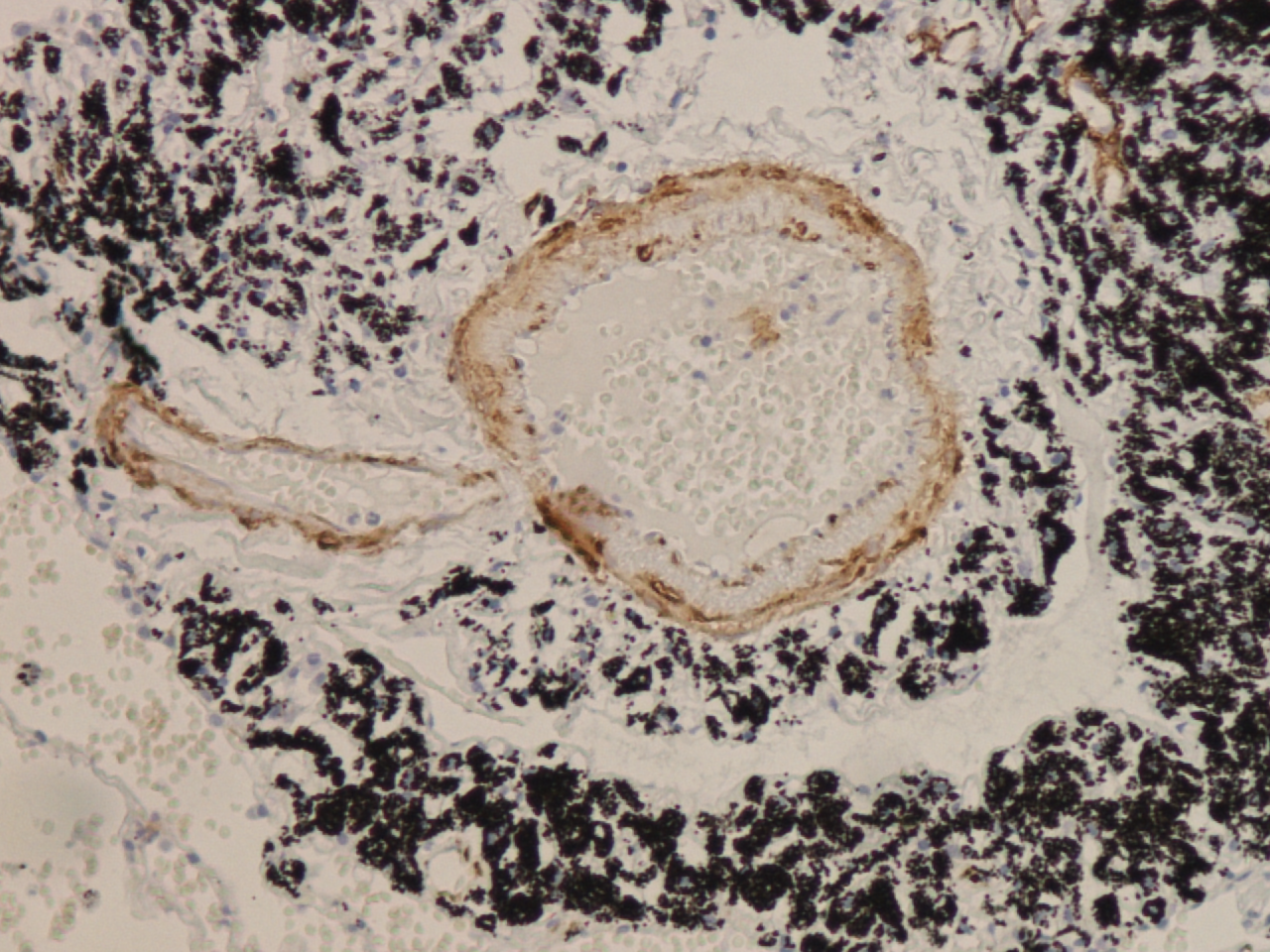

Supplement: S1 File — (ZIP) [file pone.0308871.s001.zip › TERT in Fig 2A/Human PH4.tif]

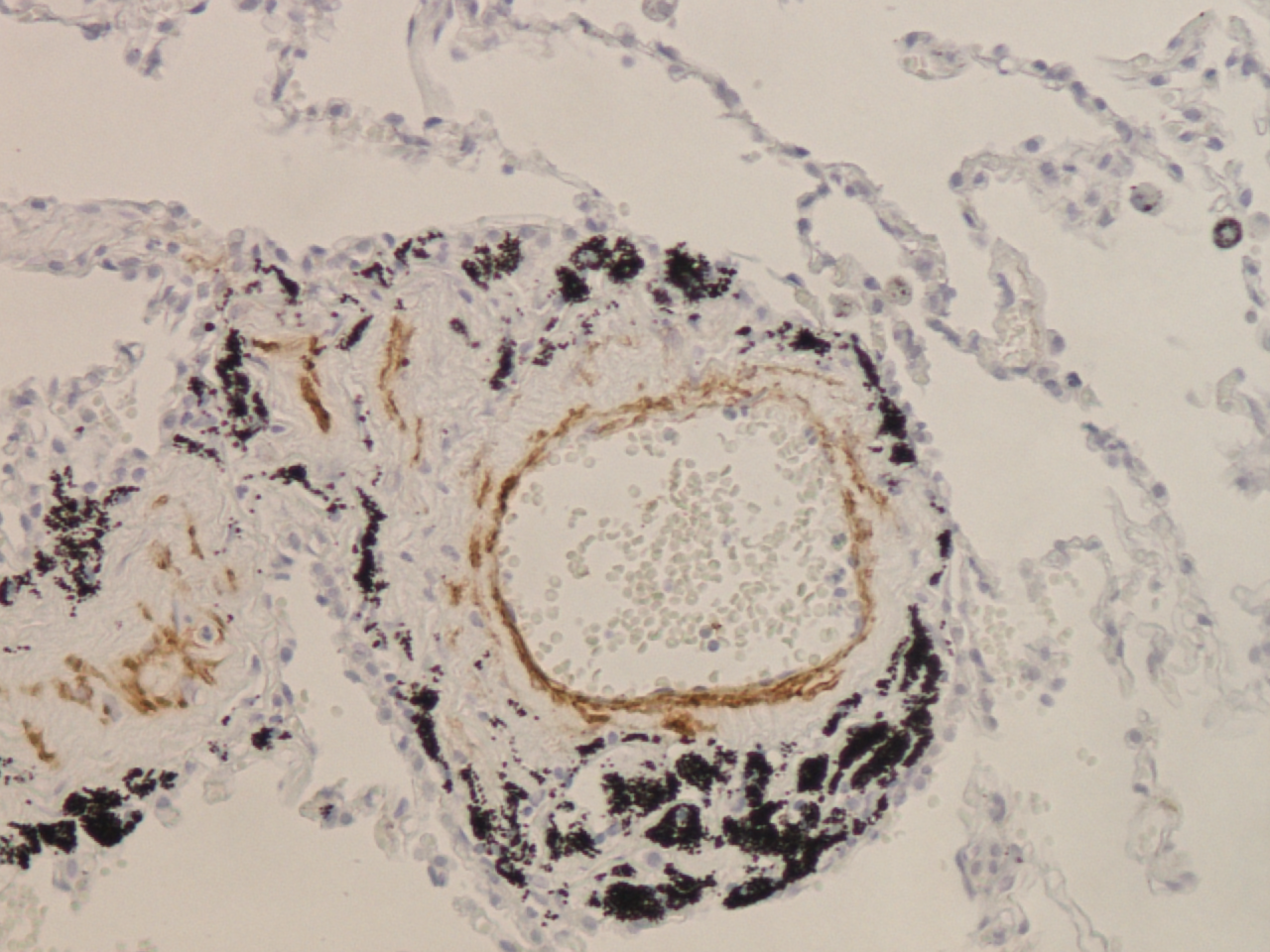

Supplement: S1 File — (ZIP) [file pone.0308871.s001.zip › TERT in Fig 2A/Human Con1.tif]

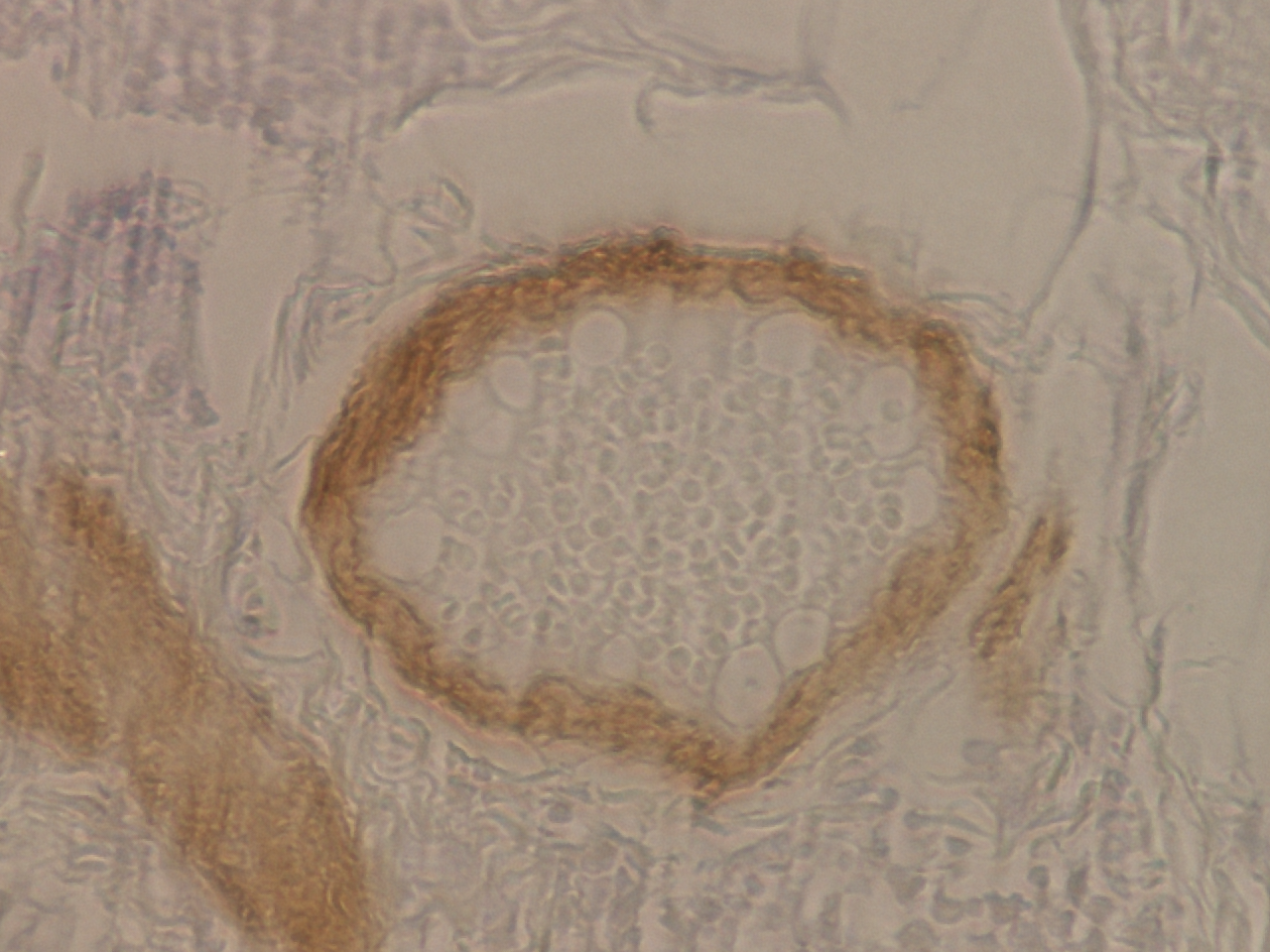

Supplement: S1 File — (ZIP) [file pone.0308871.s001.zip › TERT in Fig 2A/Rat-H+A2.tif]

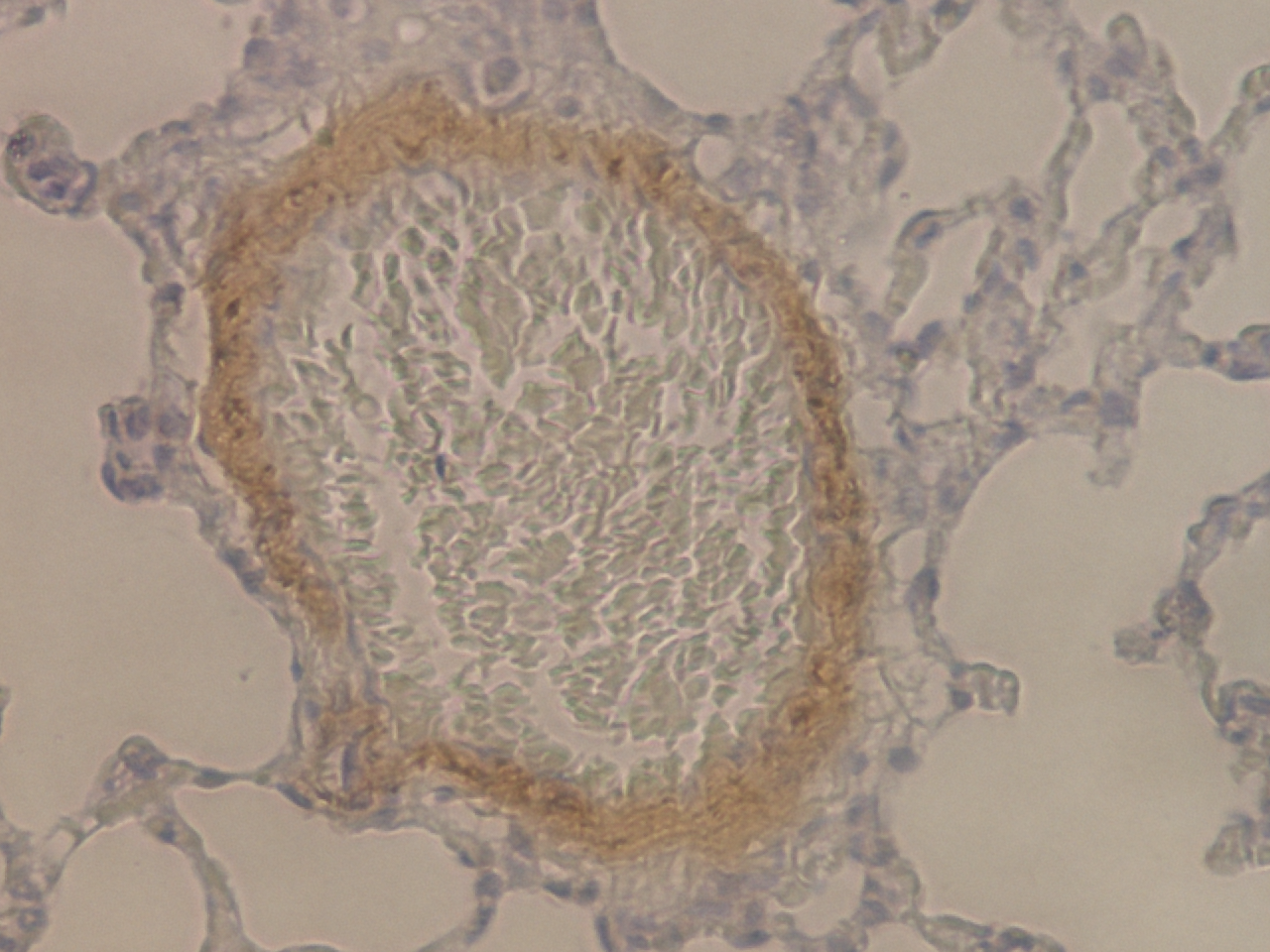

Supplement: S1 File — (ZIP) [file pone.0308871.s001.zip › TERT in Fig 2A/Rat-Hyp6.tif]

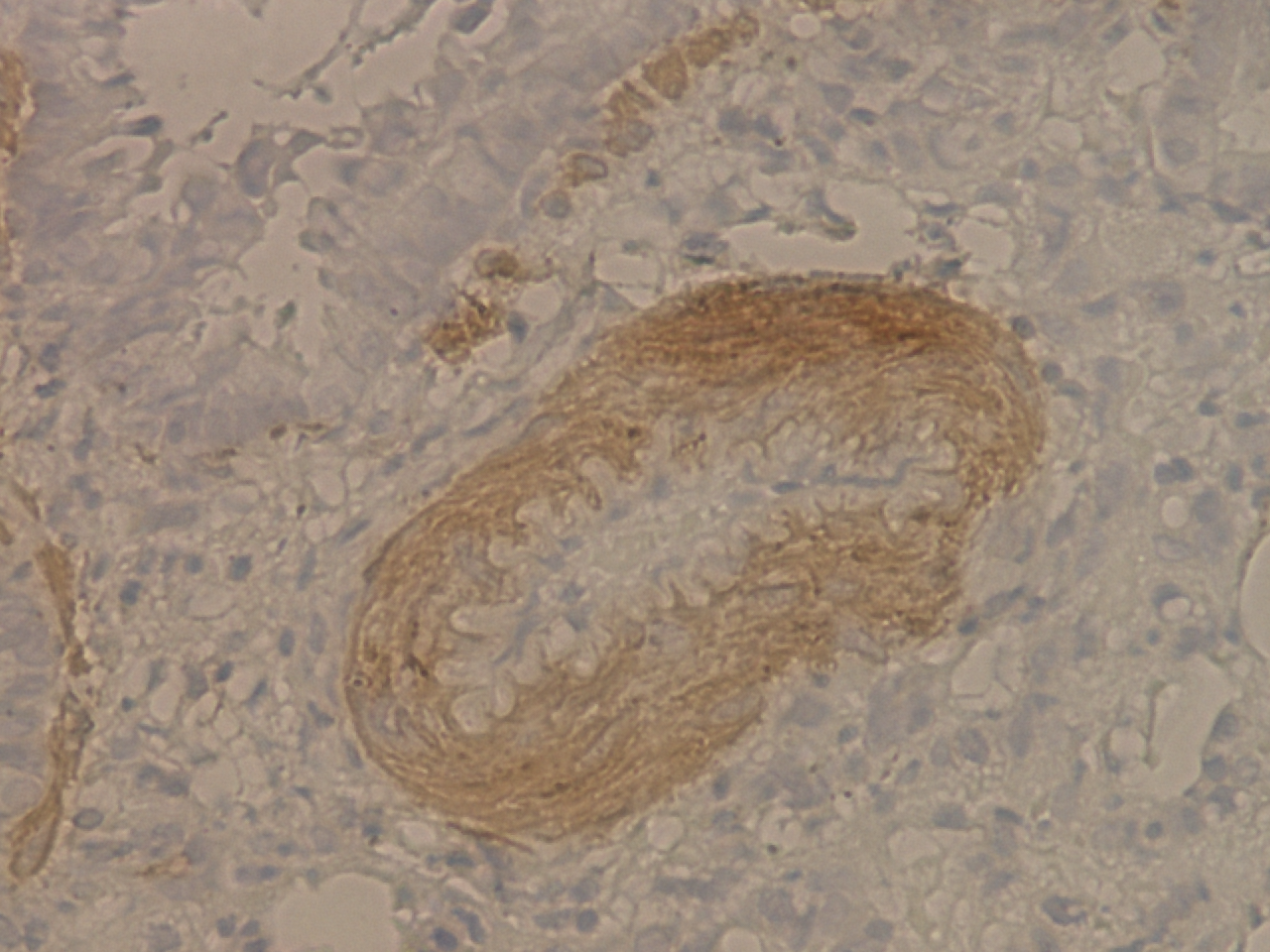

Supplement: S1 File — (ZIP) [file pone.0308871.s001.zip › TERT in Fig 2A/Rat-MCT2.tif]

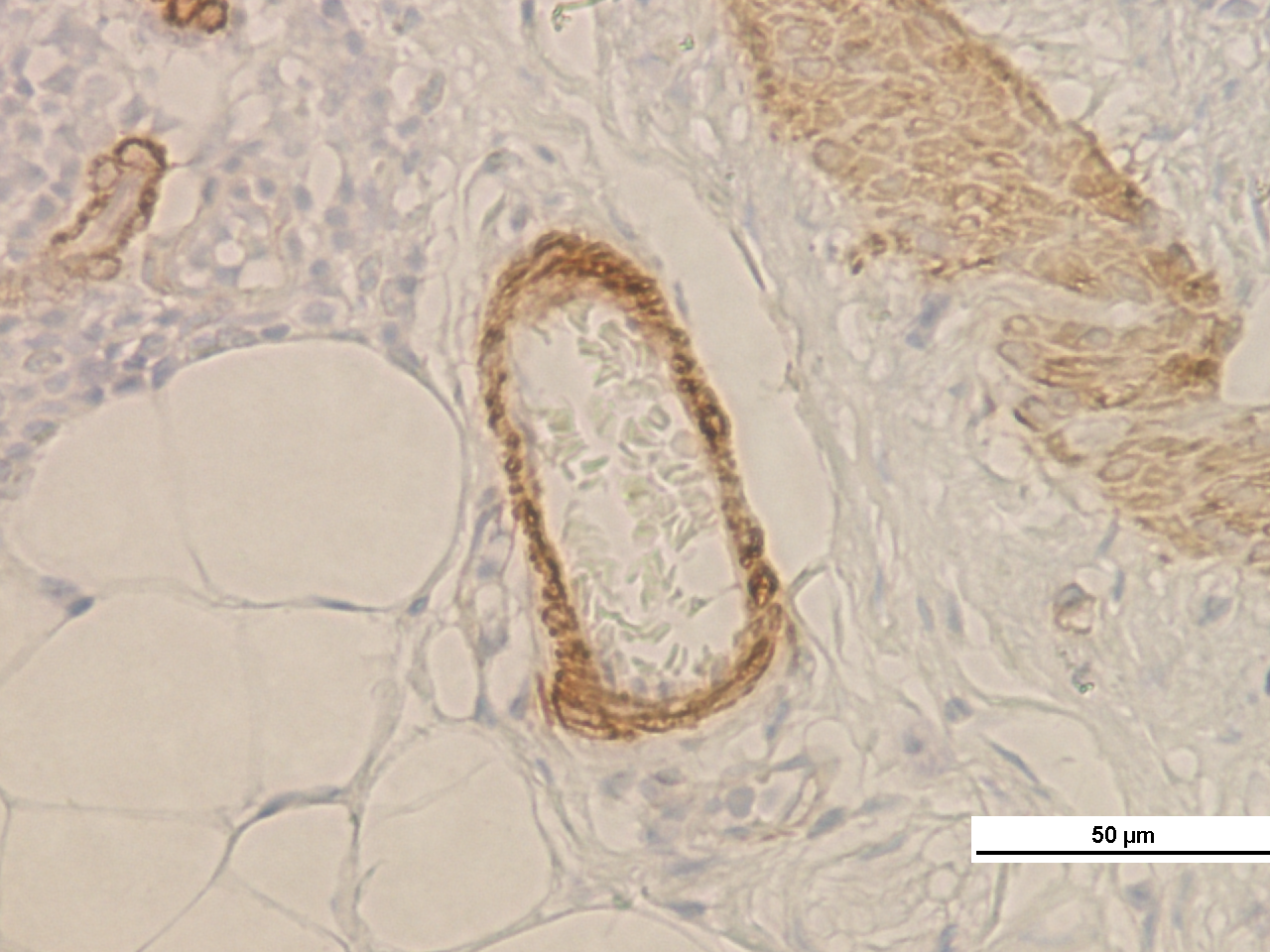

Supplement: S1 File — (ZIP) [file pone.0308871.s001.zip › TERT in Fig 2A/Rat-Con4.tif]

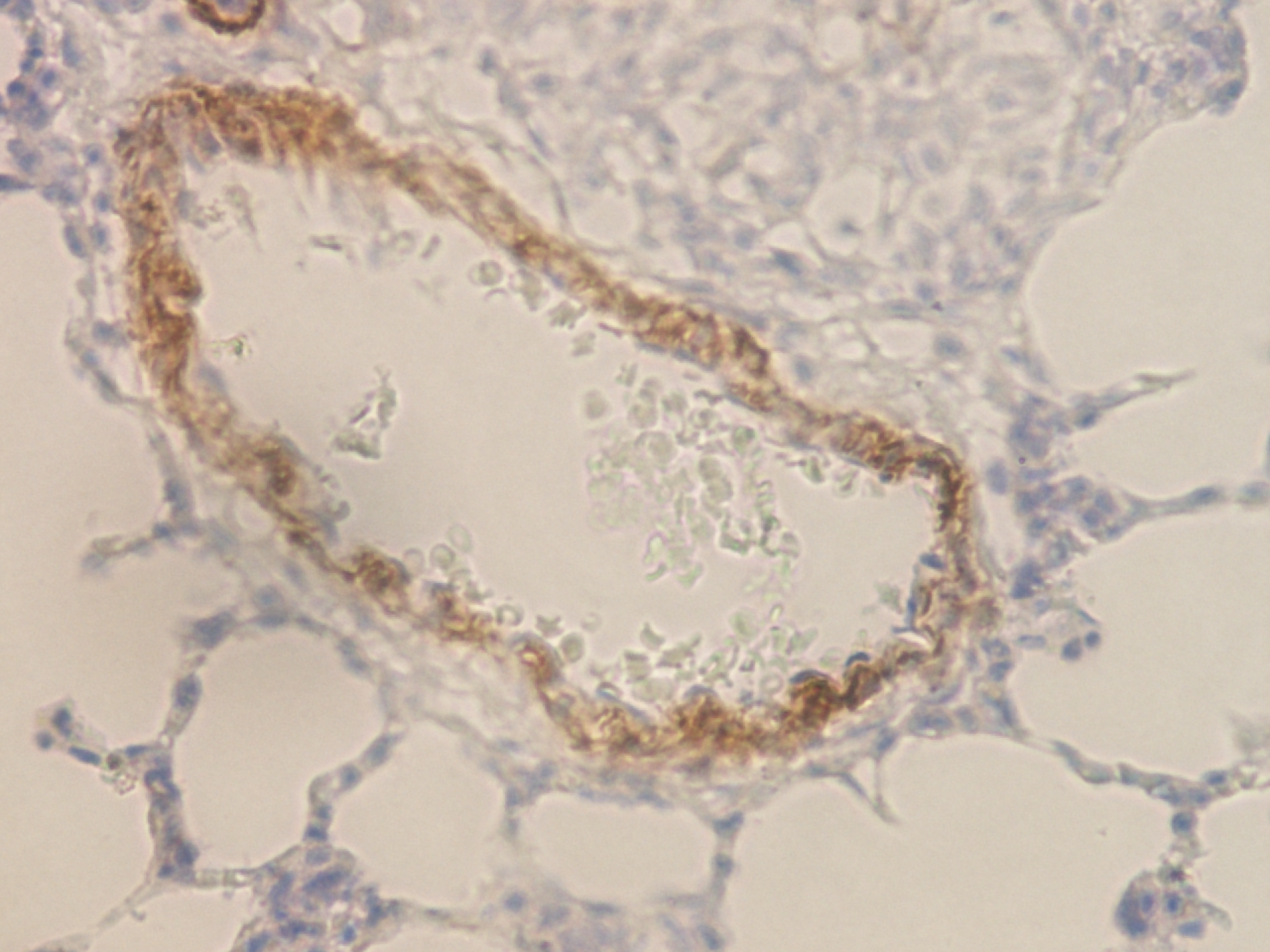

Supplement: S1 File — (ZIP) [file pone.0308871.s001.zip › TERT in Fig 2A/Rat-Con5.tif]

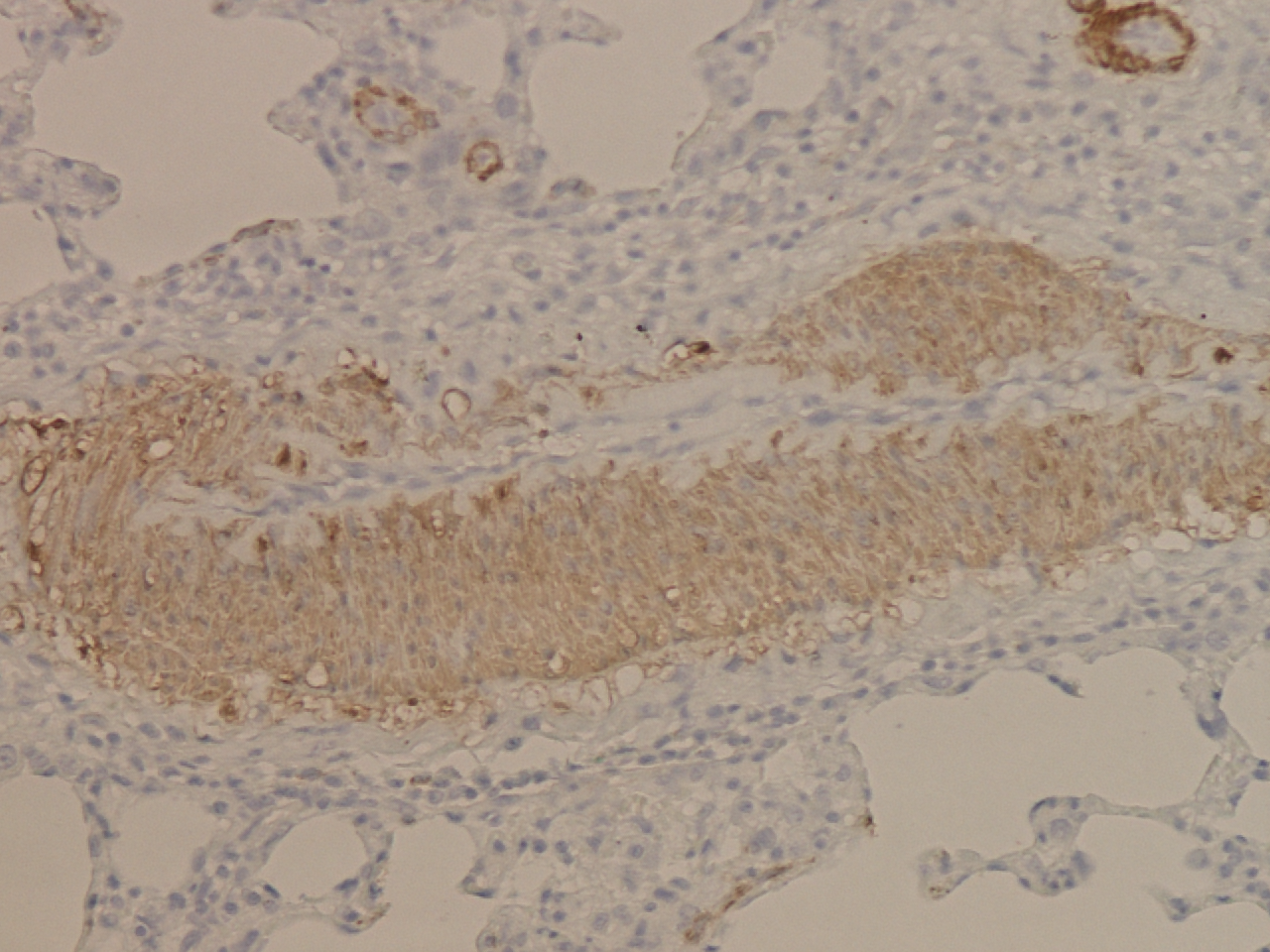

Supplement: S1 File — (ZIP) [file pone.0308871.s001.zip › TERT in Fig 2A/Rat-MCT3.tif]

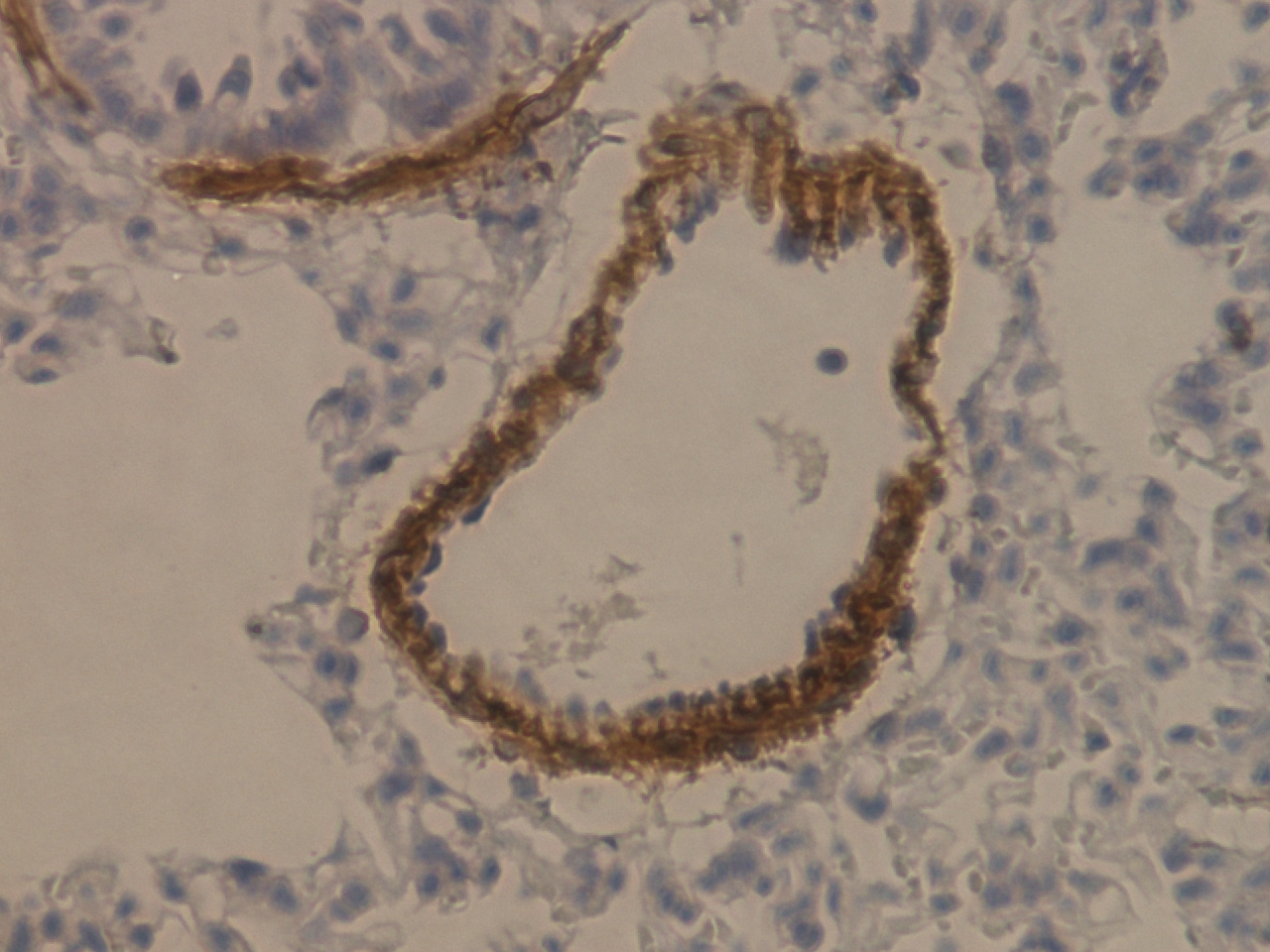

Supplement: S1 File — (ZIP) [file pone.0308871.s001.zip › TERT in Fig 2A/Rat-H+A3.tif]

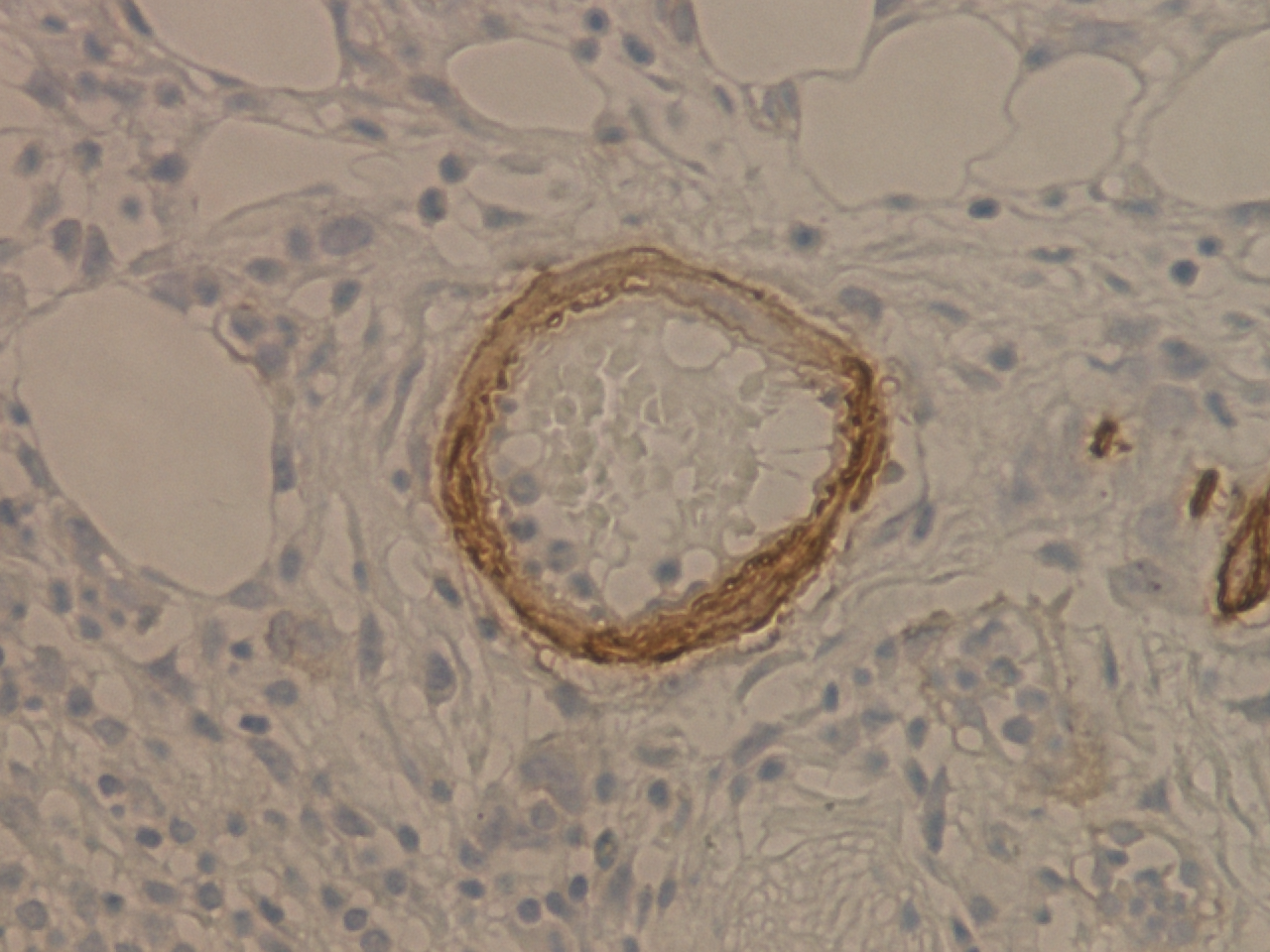

Supplement: S1 File — (ZIP) [file pone.0308871.s001.zip › TERT in Fig 2A/Rat-H+A1.tif]

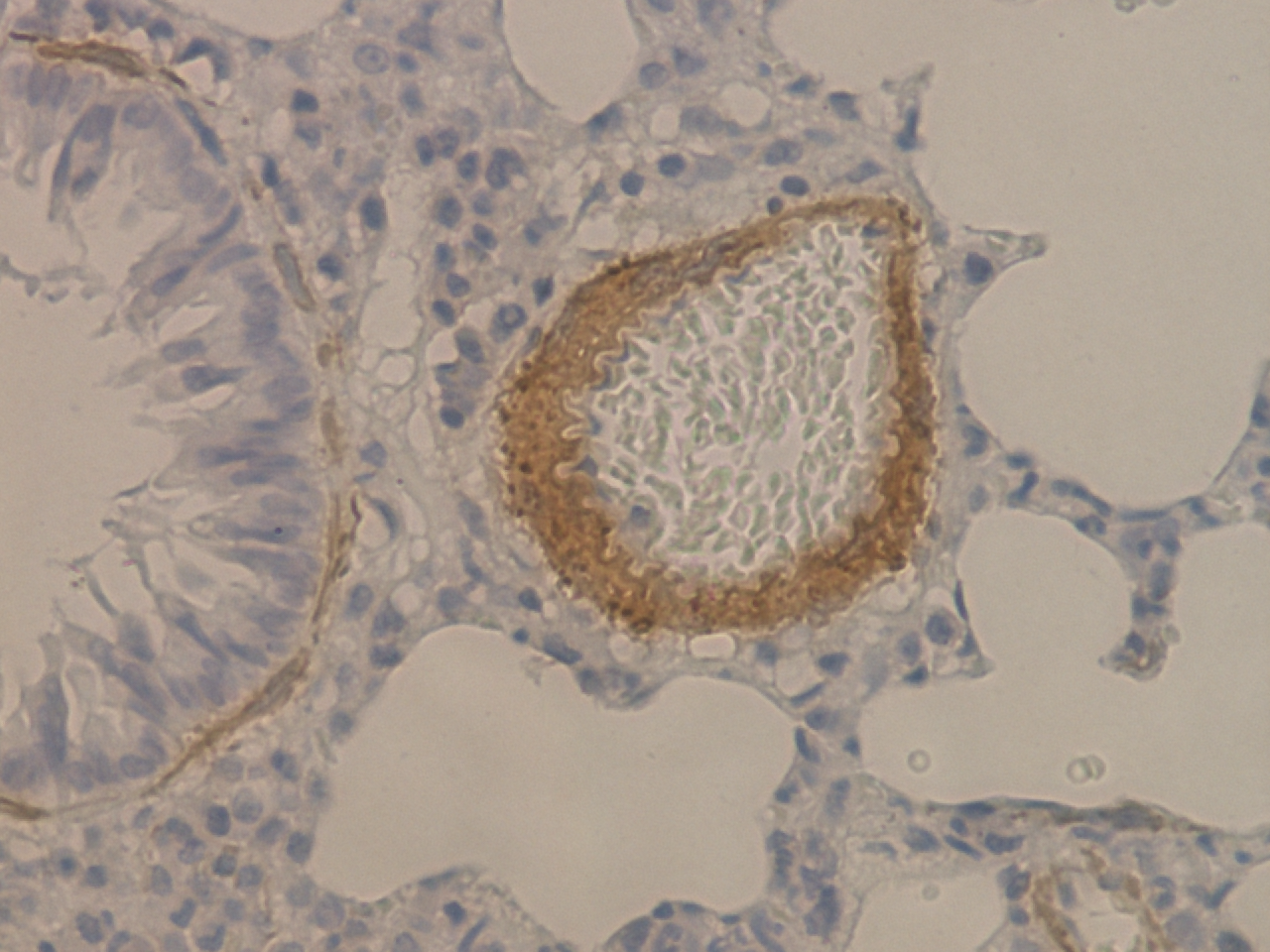

Supplement: S1 File — (ZIP) [file pone.0308871.s001.zip › TERT in Fig 2A/Rat-Hyp5.tif]

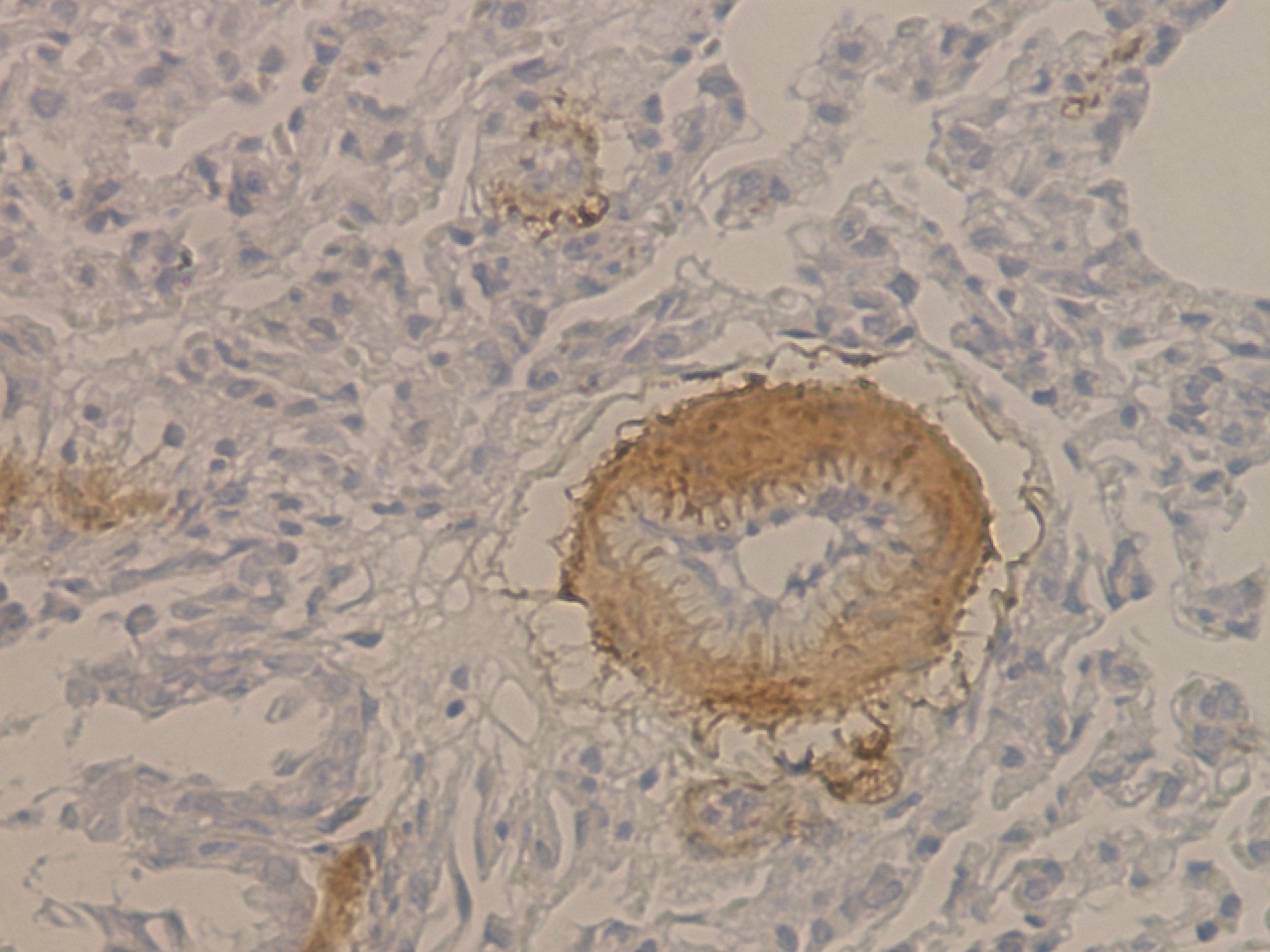

Supplement: S1 File — (ZIP) [file pone.0308871.s001.zip › TERT in Fig 2A/Rat-MCT1.tif]

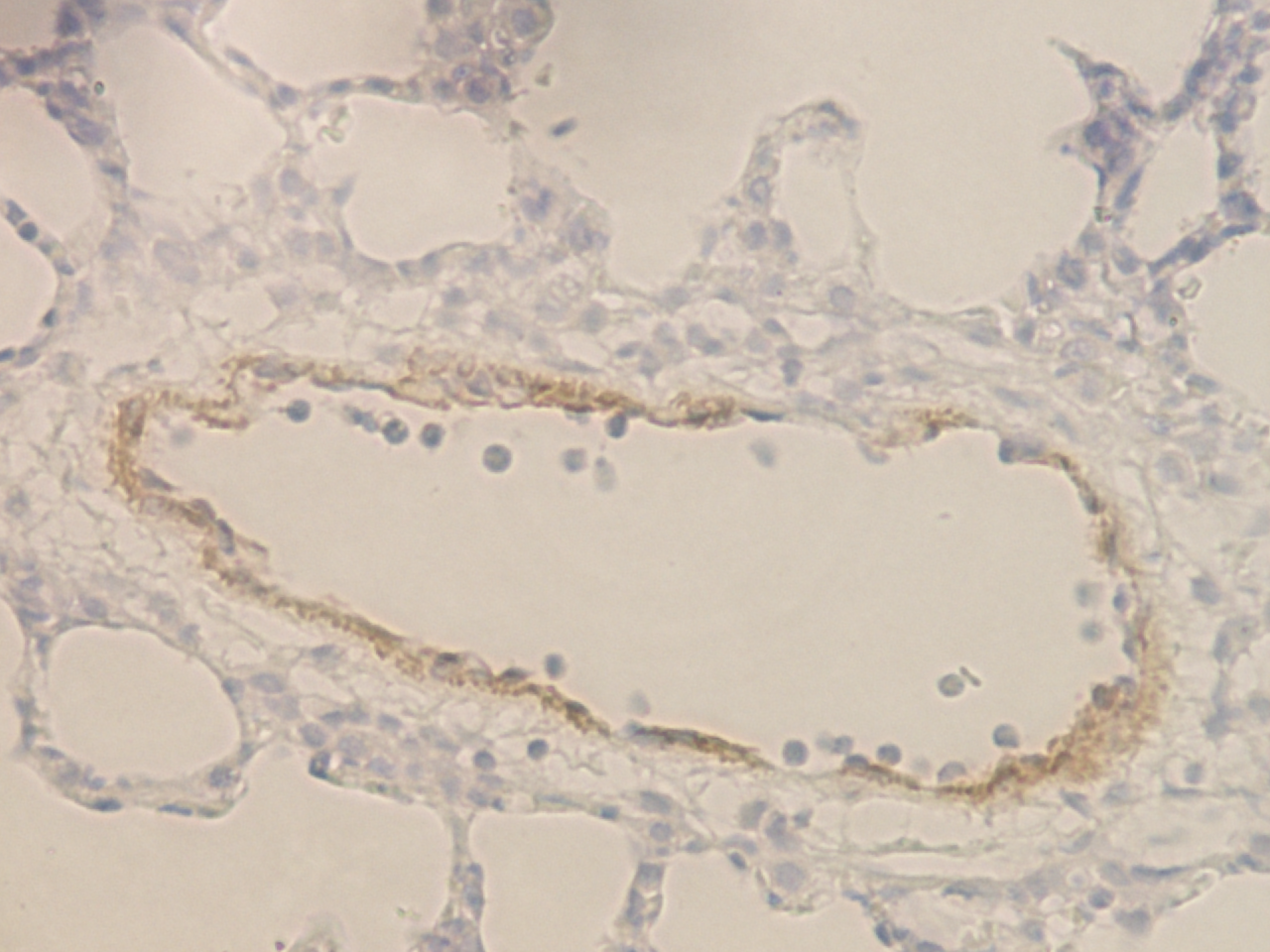

Supplement: S1 File — (ZIP) [file pone.0308871.s001.zip › TERT in Fig 2A/Rat-Con6.tif]

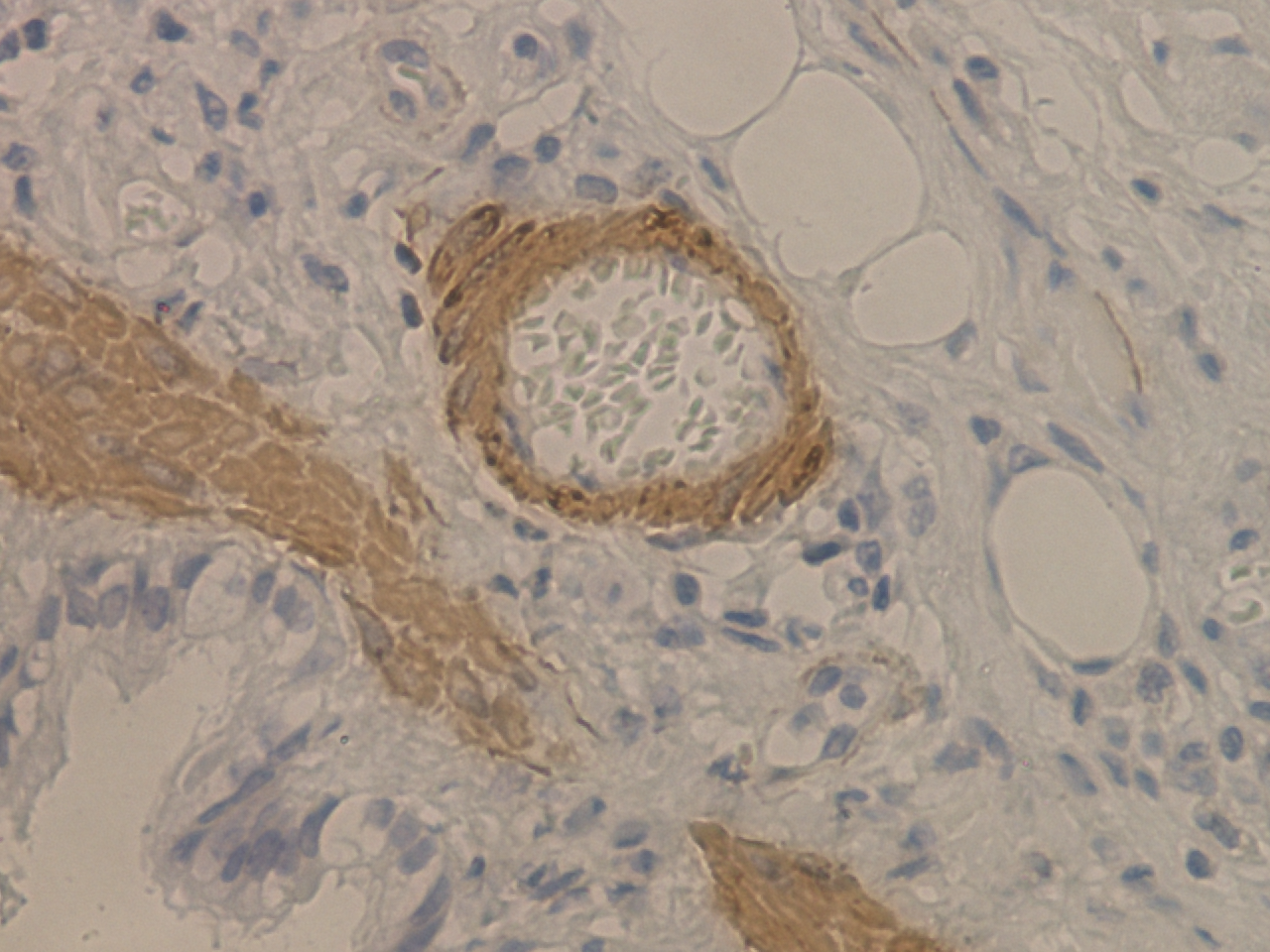

Supplement: S1 File — (ZIP) [file pone.0308871.s001.zip › TERT in Fig 2A/Rat-Hyp4.tif]

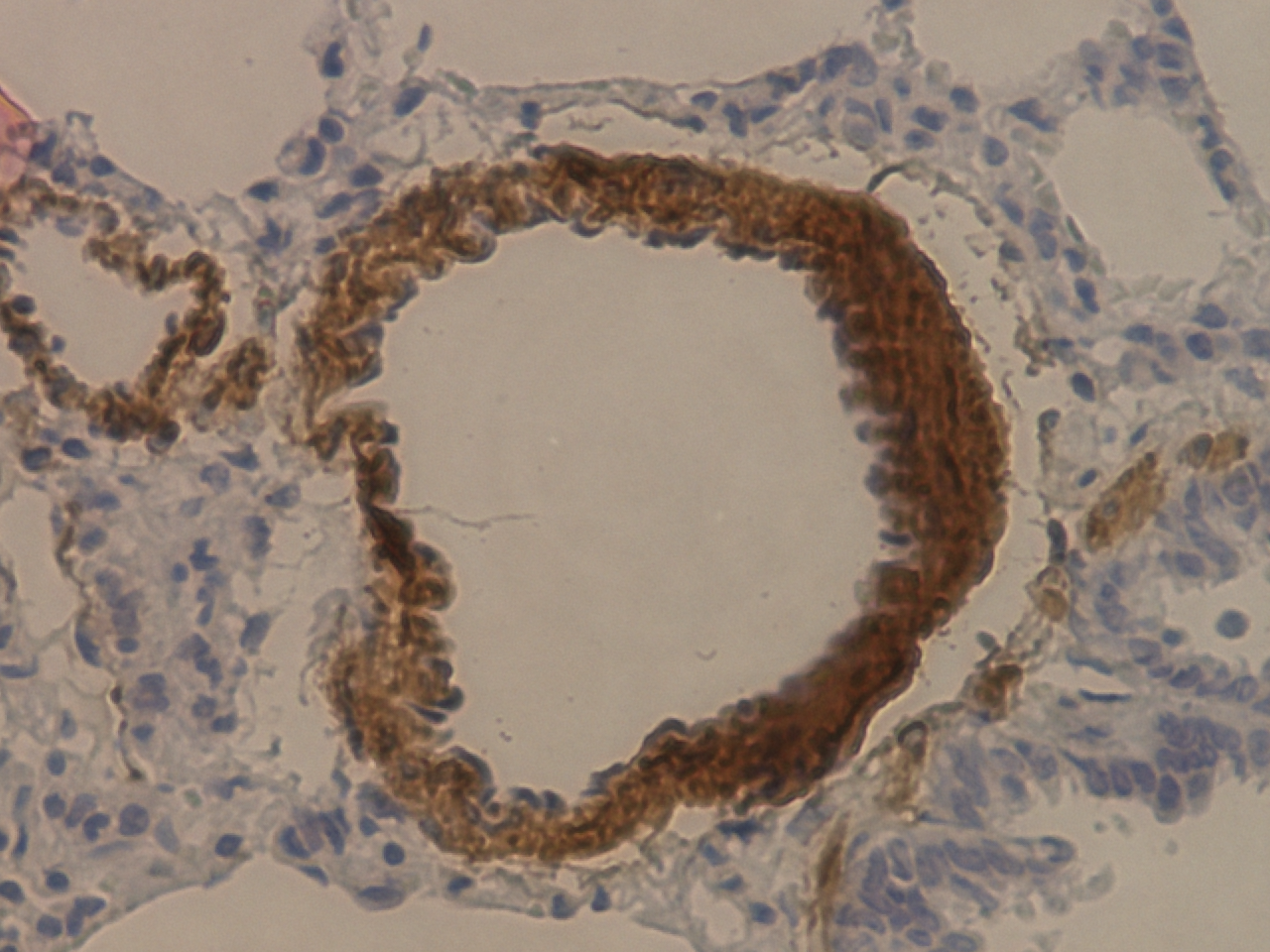

Supplement: S1 File — (ZIP) [file pone.0308871.s001.zip › TERT in Fig 2A/Rat-H+A4.tif]

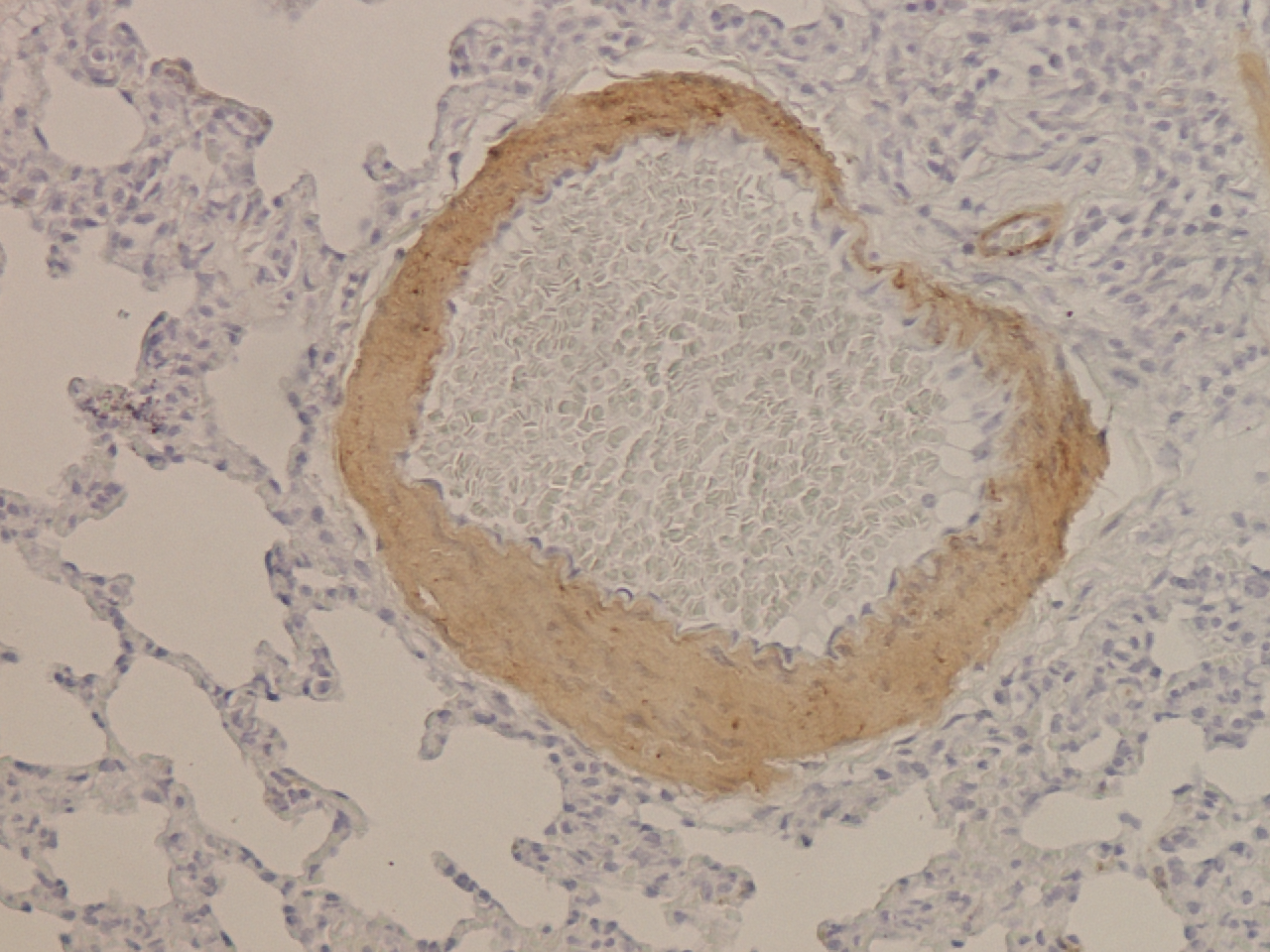

Supplement: S1 File — (ZIP) [file pone.0308871.s001.zip › TERT in Fig 2A/Rat-MCT4.tif]

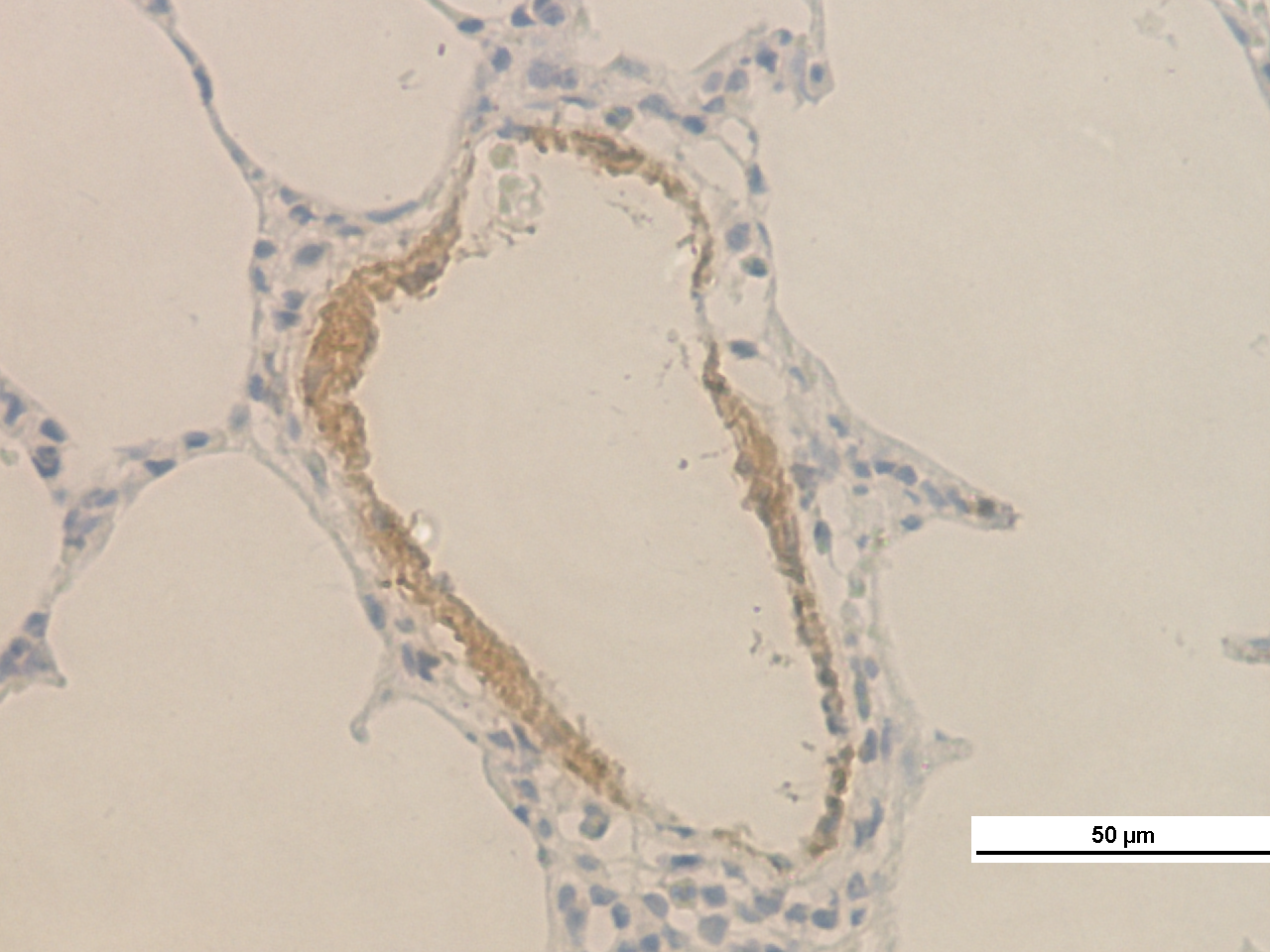

Supplement: S1 File — (ZIP) [file pone.0308871.s001.zip › TERT in Fig 2A/Rat-Con2.tif]

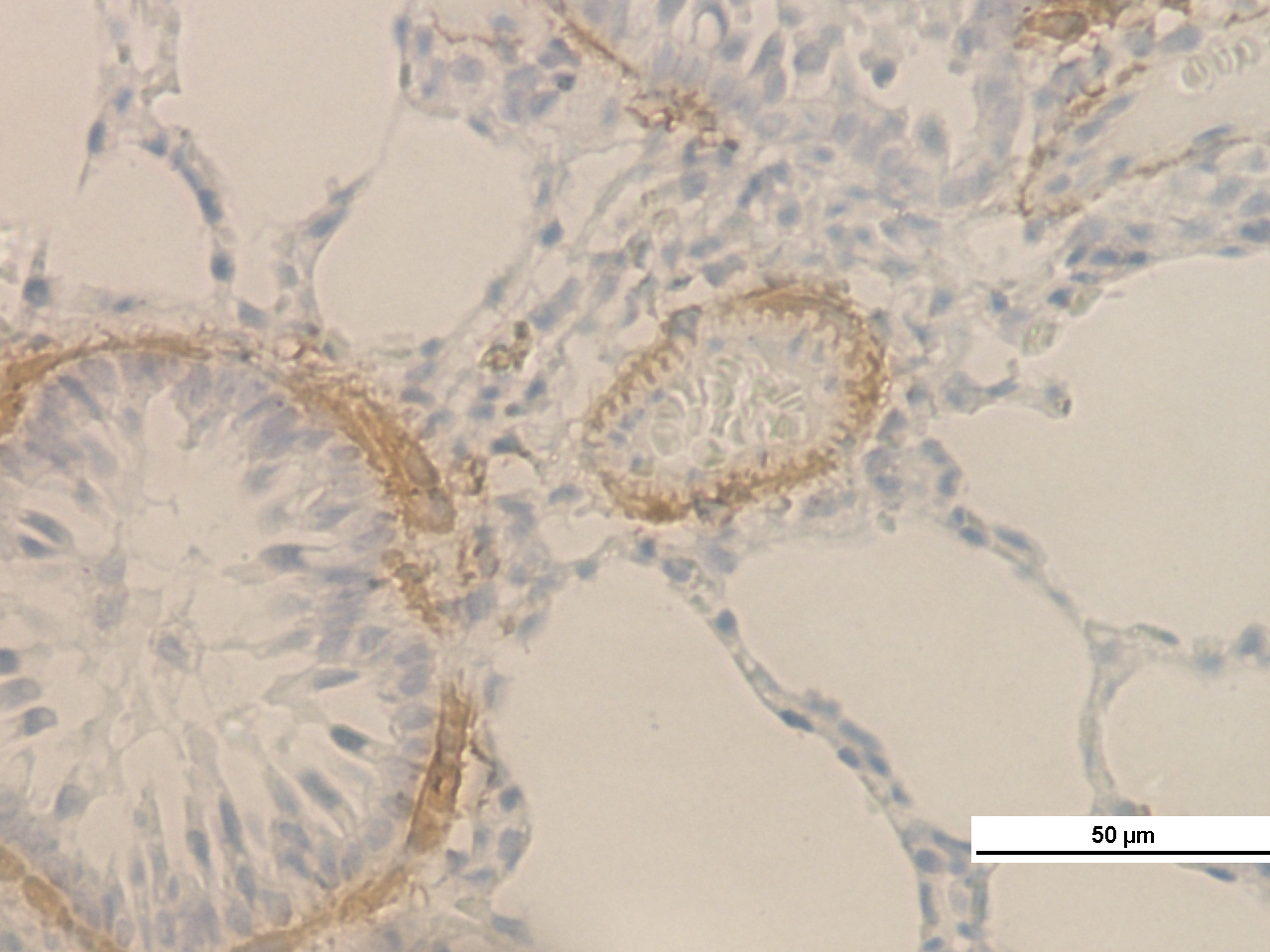

Supplement: S1 File — (ZIP) [file pone.0308871.s001.zip › TERT in Fig 2A/Rat-Con3.tif]

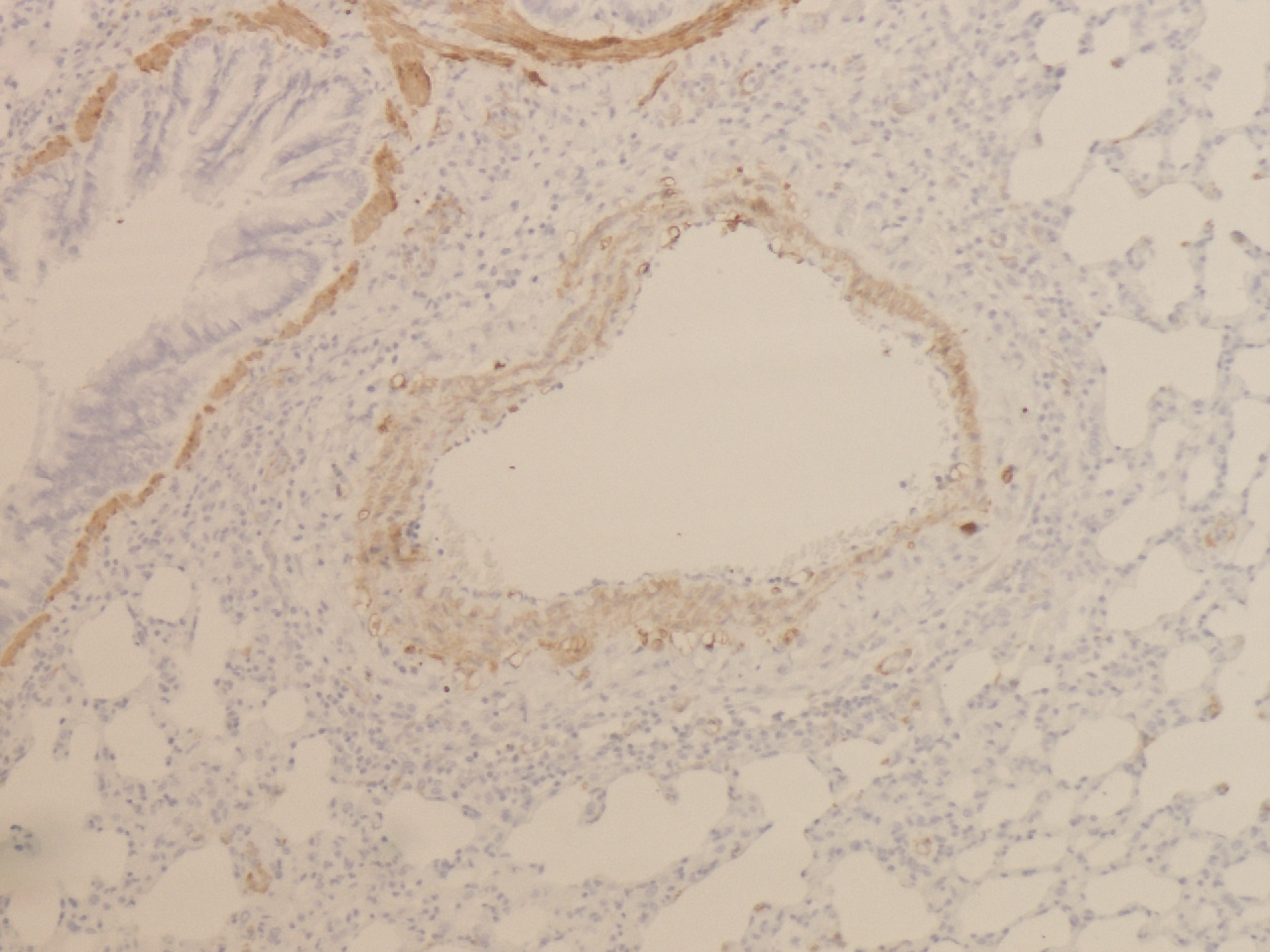

Supplement: S1 File — (ZIP) [file pone.0308871.s001.zip › TERT in Fig 2A/Rat-MCT5.tif]

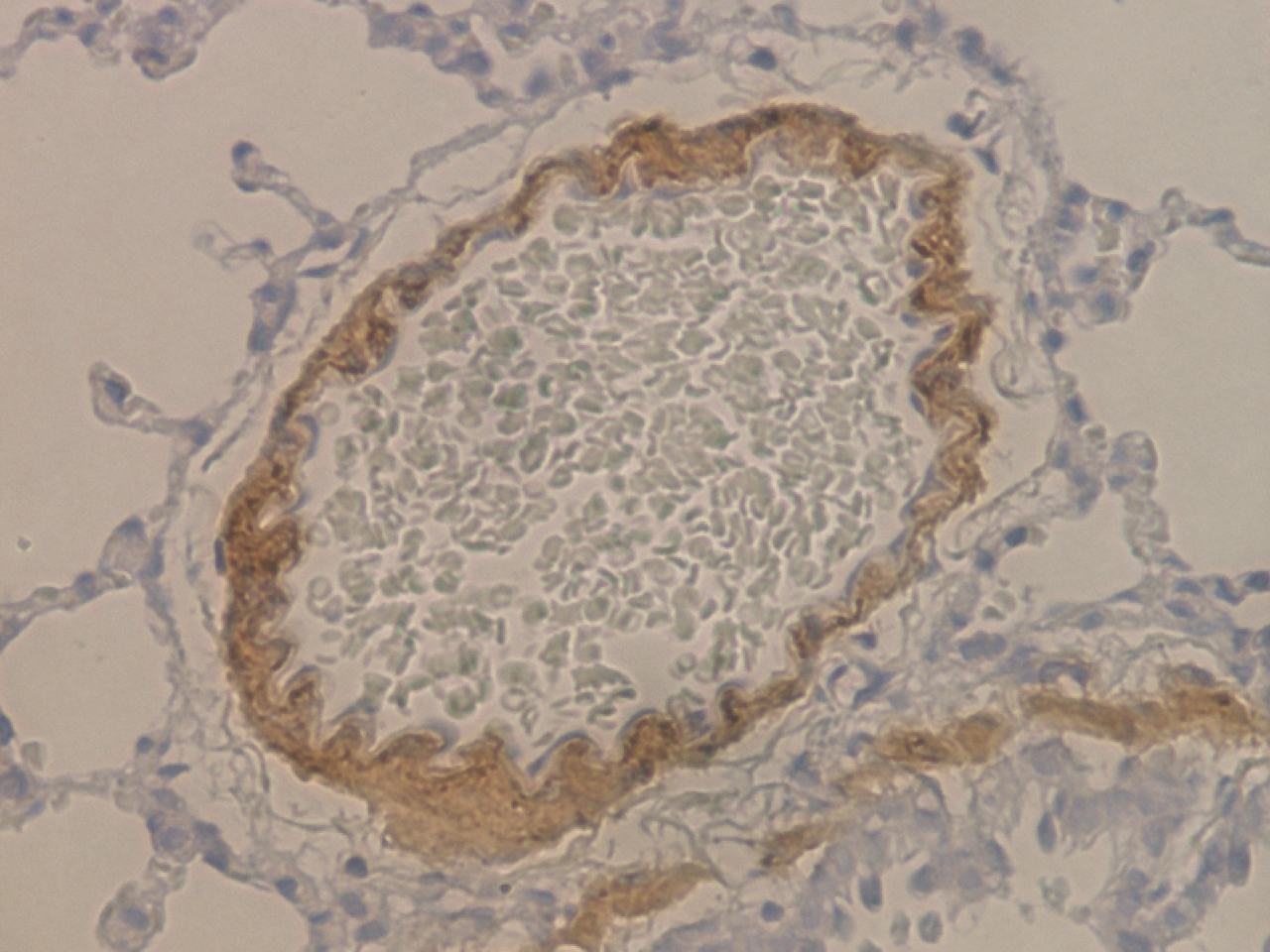

Supplement: S1 File — (ZIP) [file pone.0308871.s001.zip › TERT in Fig 2A/Rat-H+A5.tif]

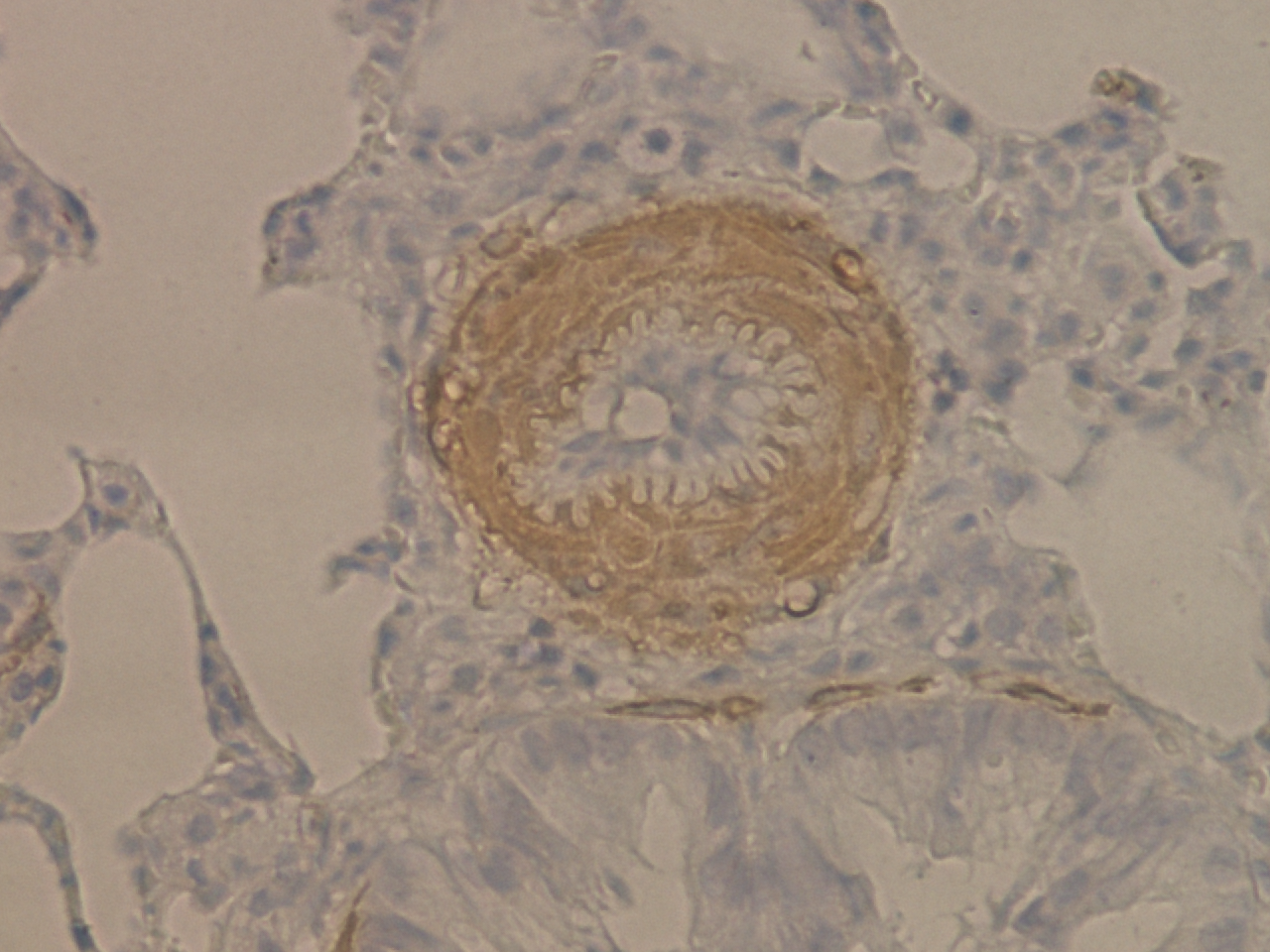

Supplement: S1 File — (ZIP) [file pone.0308871.s001.zip › TERT in Fig 2A/Rat-Hyp1.tif]

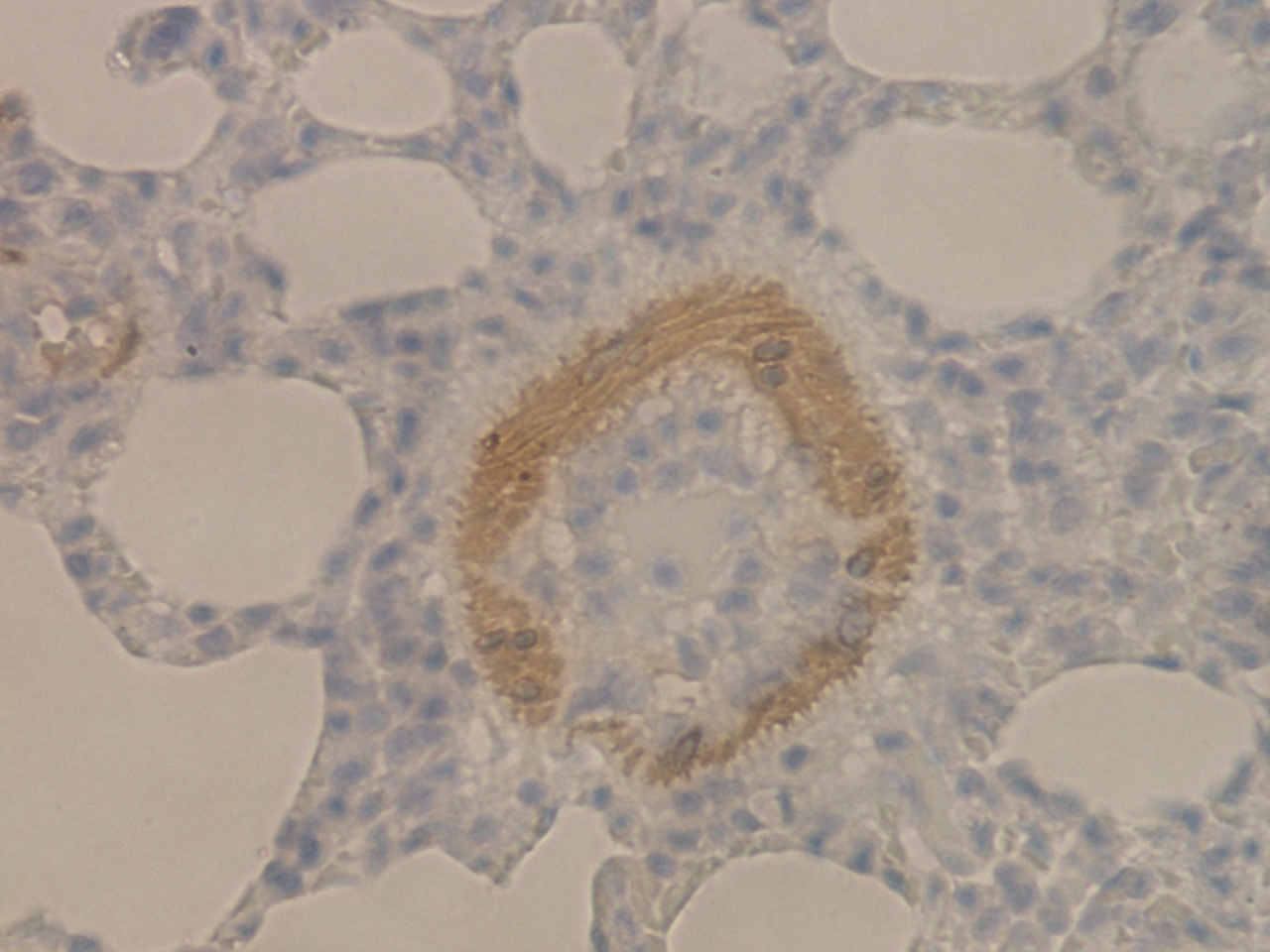

Supplement: S1 File — (ZIP) [file pone.0308871.s001.zip › TERT in Fig 2A/Rat-Hyp3.tif]

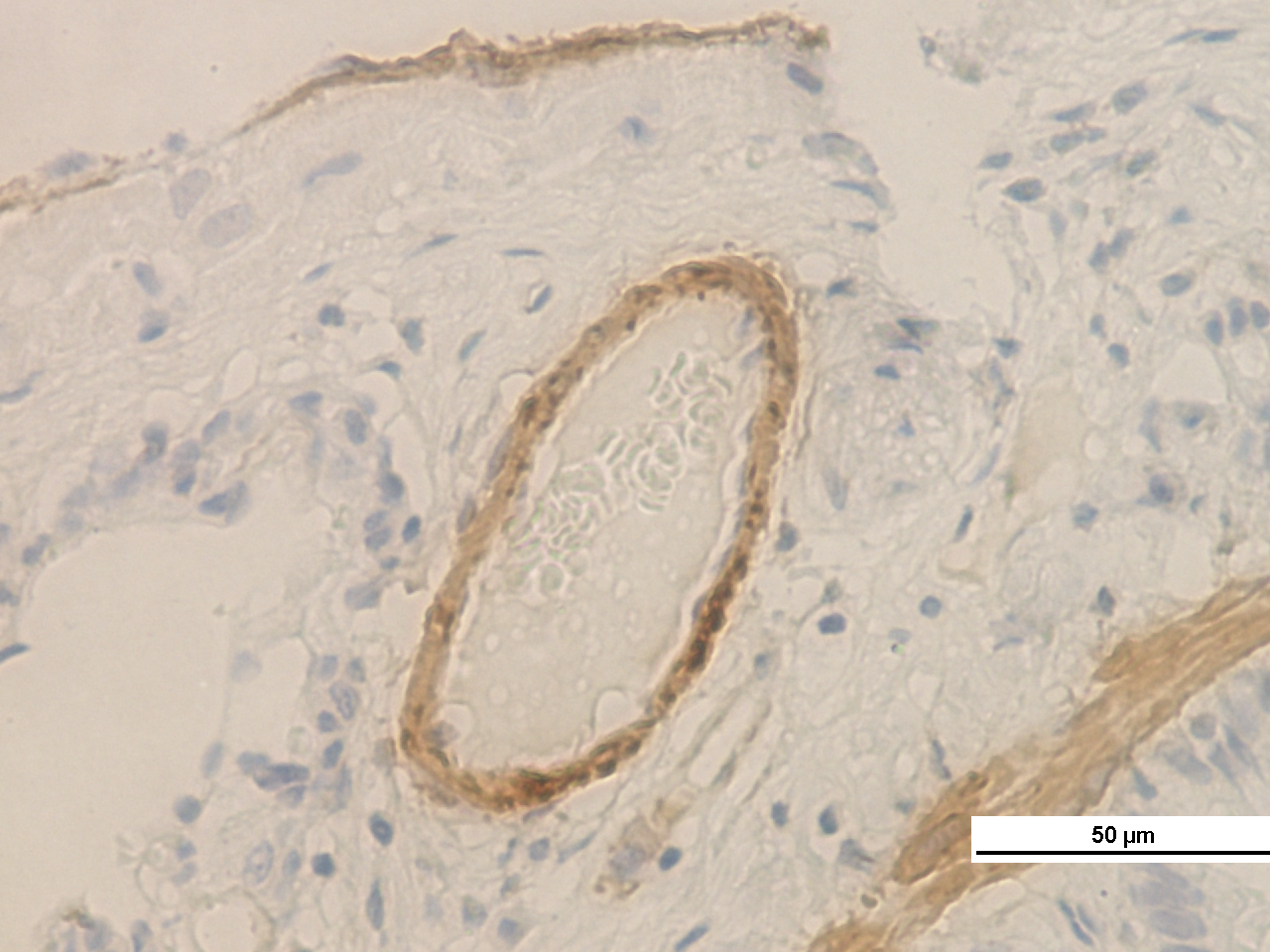

Supplement: S1 File — (ZIP) [file pone.0308871.s001.zip › TERT in Fig 2A/Rat-Con1.tif]

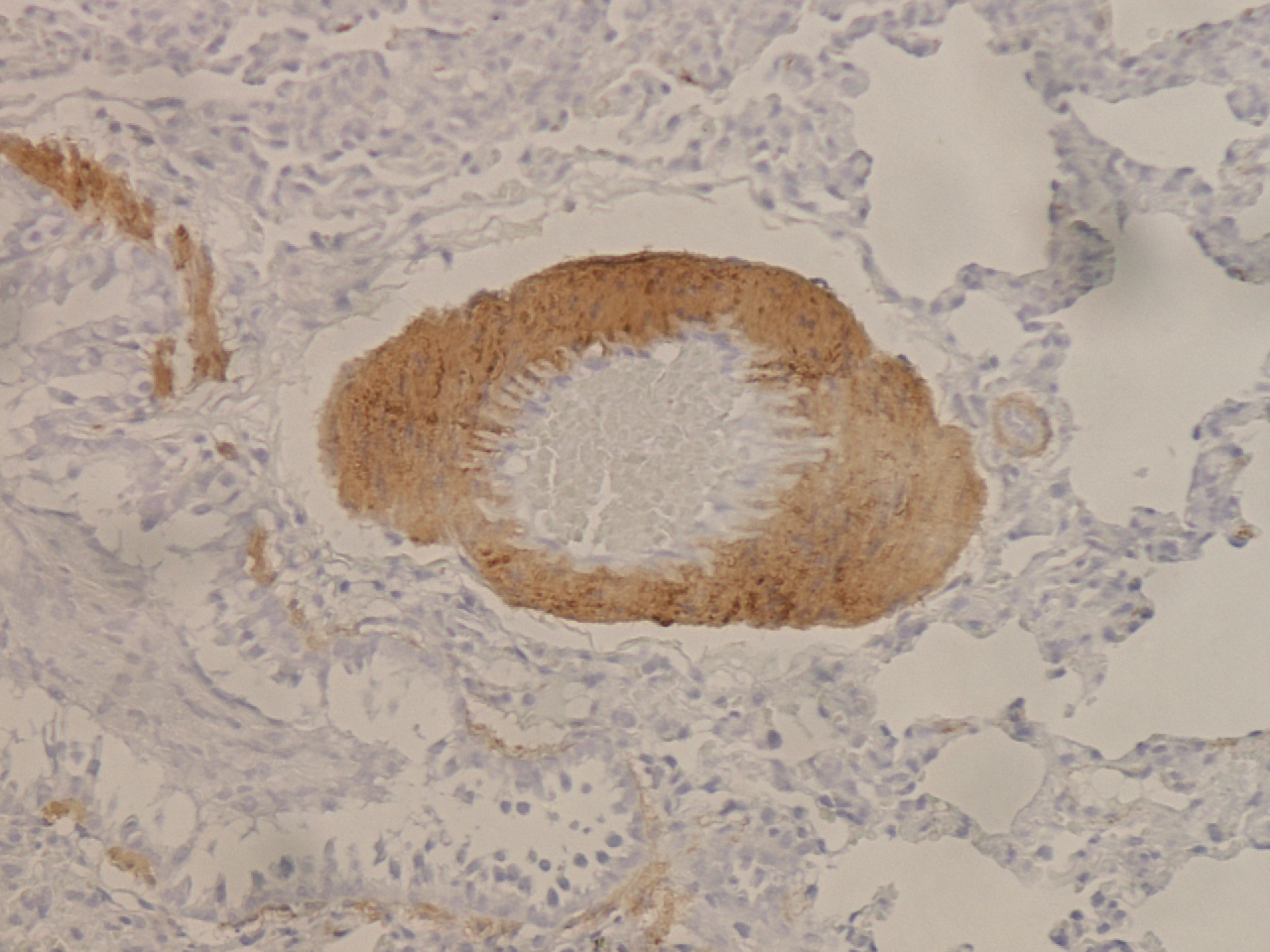

Supplement: S1 File — (ZIP) [file pone.0308871.s001.zip › TERT in Fig 2A/Rat-MCT6.tif]

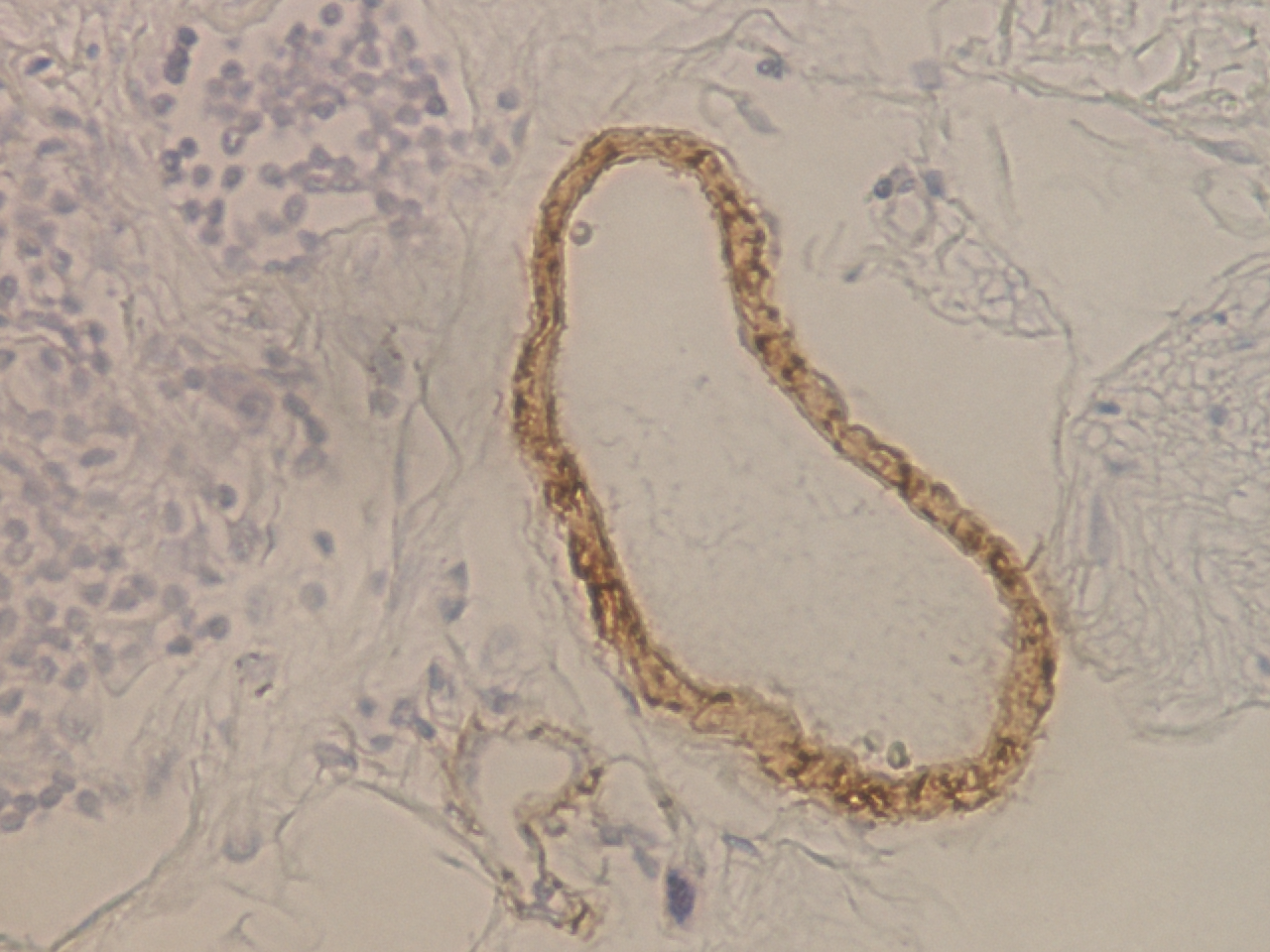

Supplement: S1 File — (ZIP) [file pone.0308871.s001.zip › TERT in Fig 2A/Rat-H+A6.tif]

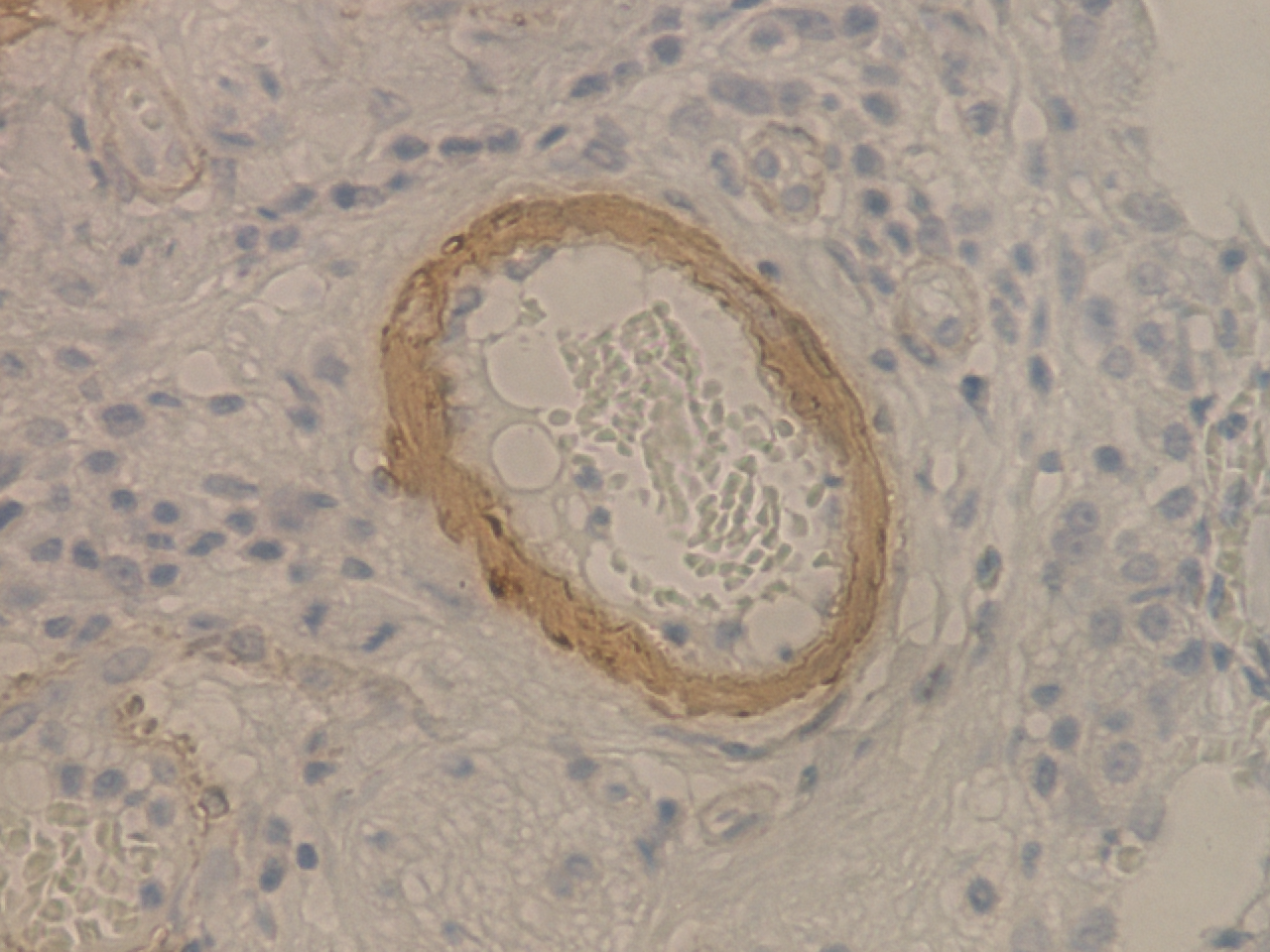

Supplement: S1 File — (ZIP) [file pone.0308871.s001.zip › TERT in Fig 2A/Rat-Hyp2.tif]
